# Modeling Congenital Adrenal Hyperplasia and Testing Interventions for Adrenal Insufficiency Using Donor-Specific Reprogrammed Cells

**DOI:** 10.1016/j.celrep.2018.01.003

**Published:** 2018-01-30

**Authors:** Gerard Ruiz-Babot, Mariya Balyura, Irene Hadjidemetriou, Sharon J. Ajodha, David R. Taylor, Lea Ghataore, Norman F. Taylor, Undine Schubert, Christian G. Ziegler, Helen L. Storr, Maralyn R. Druce, Evelien F. Gevers, William M. Drake, Umasuthan Srirangalingam, Gerard S. Conway, Peter J. King, Louise A. Metherell, Stefan R. Bornstein, Leonardo Guasti

**Affiliations:** 1 Centre for Endocrinology, William Harvey Research Institute, Barts and the London School of Medicine and Dentistry, Queen Mary University of London, EC1M 6BQ London, UK; 2 University Hospital Carl Gustav Carus, Department of Medicine III, Technische Universität Dresden, 01307 Dresden, Germany; 3 Department of Clinical Biochemistry, King’s College Hospital NHS Foundation Trust, Denmark Hill, SE5 9RS London, UK; 4 Department of Endocrinology, University College London Hospitals, NW1 2PG London, UK; 5 Paul Langerhans Institute Dresden of Helmholtz Centre Munich at University Clinic Carl Gustav Carus of TU Dresden Faculty of Medicine, Technische Universität Dresden, DZD-German Centre for Diabetes Research, 01307 Dresden, Germany; 6 Center for Regenerative Therapies, Technische Universität Dresden, 01307 Dresden, Germany; 7 Diabetes and Nutritional Sciences Division, King’s College London, WC2R 2LS London, UK

**Keywords:** steroidogenic cells, adrenal cortex, steroidogenic factor 1, NR5A1, reprogramming, urine-derived stem cells, adrenal insufficiency, congenital adrenal hyperplasia, disease modeling, transplantation

## Abstract

Adrenal insufficiency is managed by hormone replacement therapy, which is far from optimal; the ability to generate functional steroidogenic cells would offer a unique opportunity for a curative approach to restoring the complex feedback regulation of the hypothalamic-pituitary-adrenal axis. Here, we generated human induced steroidogenic cells (hiSCs) from fibroblasts, blood-, and urine-derived cells through forced expression of steroidogenic factor-1 and activation of the PKA and LHRH pathways. hiSCs had ultrastructural features resembling steroid-secreting cells, expressed steroidogenic enzymes, and secreted steroid hormones in response to stimuli. hiSCs were viable when transplanted into the mouse kidney capsule and intra-adrenal. Importantly, the hypocortisolism of hiSCs derived from patients with adrenal insufficiency due to congenital adrenal hyperplasia was rescued by expressing the wild-type version of the defective disease-causing enzymes. Our study provides an effective tool with many potential applications for studying adrenal pathobiology in a personalized manner and opens venues for the development of precision therapies.

## Highlights

• Induced steroidogenic cells (hiSCs) can be derived from urine, skin, and blood cells
• hiSCs express steroidogenic enzymes and secrete cortisol in a stimulus-dependent manner
• hiSCs are viable *in vivo* after intra-adrenal or kidney capsule transplantation
• hiSCs can be used to model adrenal disorders and potentially for cell-based therapies

## Graphical Abstract

**Figure:**
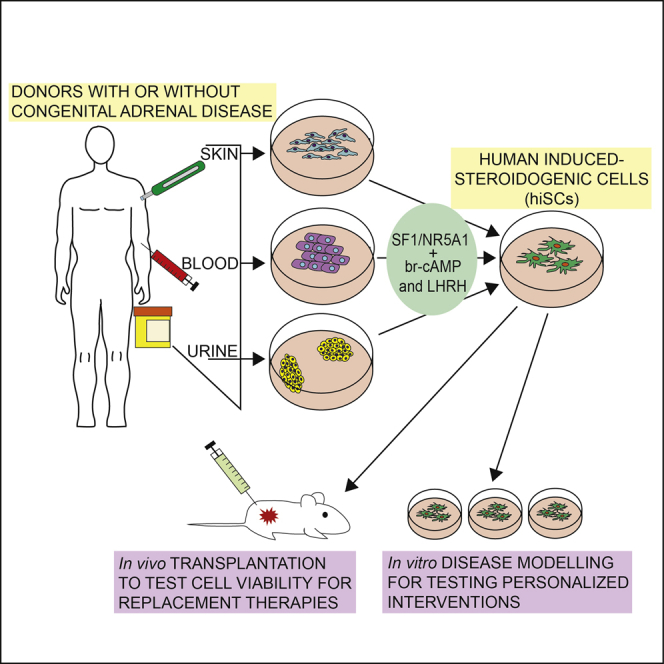

## Teaser

Ruiz-Babot et al. generate functional human steroidogenic cells (hiSCs), which are responsive to both pharmacological and physiological stimuli. Moreover, the hypocortisolism in hiSCs derived from patients with congenital adrenal hyperplasia is restored to normal through the incorporation of the wild-type version of the defective disease-causing enzymes.

## Introduction

The adrenal cortex is a major steroid-producing organ, secreting glucocorticoids under the control of adrenocorticotropic hormone (ACTH), secreted by the anterior pituitary gland, and mineralocorticoids under the control of the renin-angiotensin system. Glucocorticoids affect carbohydrate metabolism and mediate the mammalian stress response, and mineralocorticoids control blood volume and salt homeostasis. Primary or secondary adrenal insufficiency (AI) results from adrenal failure or impairment of the hypothalamic-pituitary axis, respectively. In both cases, the cortex fails to secrete sufficient amounts of glucocorticoids and adrenal androgens, but in primary AI, the clinical consequences of aldosterone deficiency make this a more lethal condition. The most frequent cause of primary AI is autosomal recessive congenital adrenal hyperplasia (CAH), which results from defects in enzymes involved in steroid biosynthesis (Merke and Bornstein, 2005). Patients with AI need life-long management with exogenous steroids: this can be challenging, because no drug suitably mimics the diurnal pattern of cortisol, and objective variables measuring the quality of replacement therapy are lacking. Fine-tuning of replacement therapy leaves only a narrow margin for improvement: under-replacement can result in severe impairment of well-being and incipient crisis, whereas even subtle, chronic over-replacement has the potential to contribute to excess morbidity, including obesity, osteoporosis, hypertension, and impaired glucose tolerance. Therefore, better treatment solutions are urgently needed (Bornstein et al., 2016).

The ability to generate donor-specific and functional adrenocortical-like cells would facilitate: (1) the next generation of cell-based treatments for AI; (2) the modeling of adrenal-specific diseases; and (3) the testing of personalized interventions on cells derived from patients.

Cells with an adrenocortical-like phenotype have never been obtained in a patient-specific manner; moreover, previous attempts have resulted in cells with very limited steroidogenic potential (Crawford et al., 1997) or have used lines of embryonic stem cells (Jadhav and Jameson, 2011, Yazawa et al., 2011), mesenchymal stem cells (Gondo et al., 2004, Gondo et al., 2008, Mazilu and McCabe, 2011a, Tanaka et al., 2007, Wei et al., 2012, Yazawa et al., 2006, Yazawa et al., 2009, Yazawa et al., 2010), and induced pluripotent stem cells (iPSCs) (Sonoyama et al., 2012). The prerequisite of all these studies was the forced expression of steroidogenic factor-1 (SF1), a master regulator of adrenogonadal development and function encoded by nuclear receptor subfamily 5, group A, member 1 (*NR5A1*). SF1 is a true effector of cell fate as it initiates a genetic program driving embryonic mesenchymal cells toward a steroidogenic phenotype/lineage (Schimmer and White, 2010), and SF1 mutations can result in adrenal insufficiency (Achermann et al., 2001). Other than SF1, additional transcription factors (TFs), such as wilms tumor 1 (WT1), CBP/p300-interacting transactivator 2 (CITED2), pre-B cell leukemia transcription factor 1 (PBX1), and dosage-sensitive sex reversal, adrenal hypoplasia critical region, on chromosome X, gene 1 (DAX1) have been shown to be key determinants of adrenal cortex development (Yates et al., 2013). Moreover, multiple pathways have been implicated in the fine-tuned regulation of adrenocortical development, zonation, and self-renewal (Lerario et al., 2017).

Here, we show an efficient protocol to reprogram, through stable expression of SF1 and activation of the protein kinase A (PKA) pathway and in the presence of luteinizing-hormone-releasing hormone (LHRH), easily accessible sources of cells from humans (blood, skin, and urine), resulting in human induced steroidogenic cells (hiSCs). These hiSCs expressed steroidogenic enzymes and secreted cortisol in a stimulus-dependent manner. hiSCs could be efficiently exploited to study donor-specific disease pathobiology and to test interventions, such as restoration of eucortisolism in hiSCs from CAH patients. Moreover, as a first step to assess whether hiSCs can be a viable option for the development of cell-based treatments for AI, we performed pilot *in vivo* experiments proving the viability of hiSCs after transplantation into the adrenal glands and under the kidney capsule of mice. These experiments pave the way for further testing of hiSCs in suitable rodent models of AI, such as double adrenalectomised rats (Balyura et al., 2015, Ruiz-Babot et al., 2015).

## Results

### Establishment of Human Primary Cultures from Different Cell Sources

Primary cultures of human urine-derived stem cells (USCs), late-outgrowth endothelial progenitor cells (L-EPCs), and fibroblasts were initially established from healthy donors (Figure S1). Because L-EPCs are phenotypically indistinguishable from bone-marrow-derived endothelial cells (BMECs) (Yoder et al., 2012), the latter were also used in our experiments.

### Generation of hiSCs by Direct Lineage Conversion

**Figure 1:**
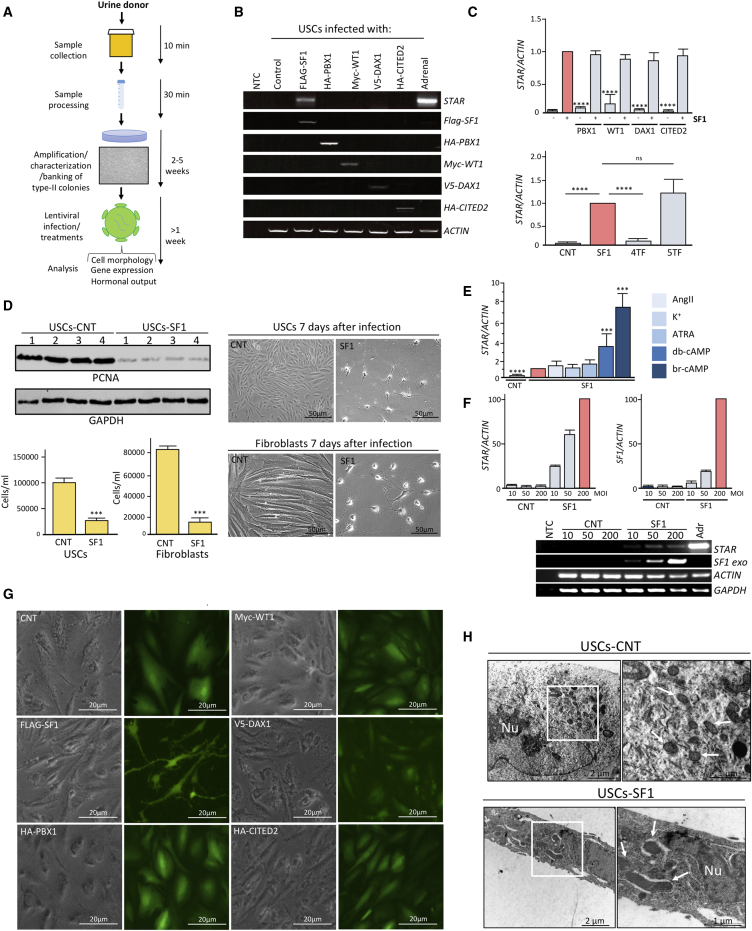
Conversion of Human Urine-Derived Stem Cells into Steroidogenic Cells (A) Schematic illustrating our strategy for urine collection, processing, and reprogramming. Urine-derived cells (USCs) were cultured in specific media, and type-II colonies amplified and characterized through flow cytometry. Then they were either banked or expanded for experiments. USCs were infected at passage two with either a lentivirus encoding a transcription factor (TF) within an IRES-GFP vector, a combination of TFs, or mock infected (MOI = 200). Cells were treated with 8-br-cAMP (100 μM) unless stated otherwise and kept in culture for at least eight days before analyses. (B) RT-PCR showing *STAR* expression on forced expression of each TF. The expression of exogenous *SF1*, *PBX1*, *WT1*, *DAX1*, and *CITED2* was assessed by RT-PCR using primers encompassing the coding- and vector- specific regions. Human adrenal cDNA was used as a positive control for endogenous *STAR* expression and, along with non-template control (NTC), as a negative control for exogenous TF expression. (C) qRT-PCR analyses of *STAR* expression on forced expression of SF1 with each TF (upper panel) and of SF1 with or without a combination of TFs (lower panel). (D) Western blot analyses of PCNA and GAPDH expression in hiSCs and mock-reprogrammed USCs from four independent donors eight days after reprogramming (top left panels); cell counting (bottom left panels) and representative images (right panels) of hiSCs obtained from USCs and fibroblasts versus mock-reprogrammed cells. Scale bars, 50 μm. (E) qRT-PCR analyses of *STAR* expression on forced expression of SF1 with or without the indicated treatments, started the day after infection for seven days. CNT, cells infected with empty control vector. (F) qRT-PCR (upper panel) and RT-PCR (lower panels) analyses of *STAR* and *SF1* expression after reprogramming USCs at different MOI of SF1 or empty control lentiviral vector (CNT). (G) Morphological changes on SF1 overexpression in USCs eight days post-infection. Scale bars, 20 μm. (H) Electron microscopy images of USCs and USCs eight days after reprogramming. Arrows point to mitochondria. Nu, nucleus. Scale bars, 2 μm (left panels) and 1 μm (right panels). Data in (C)–(F) are represented as mean ± SEM, n ≥ 3. See also Figures S1 and S2.

Lentiviral vectors encoding SF1 and other TFs (PBX1, DAX1, WT1, and CITED2) were used to infect human primary cells. The vectors co-express GFP bicistronically and contain a mammalian resistance cassette, which was used for selection (Figure S2A). Cells were transduced according to the schematic in Figure 1A and as reported in the Experimental Procedures. The expression of the steroidogenic acute regulatory protein (*STAR*) was used as a readout for initial experiments; STAR mediates the transfer of cholesterol from the outer to the inner mitochondrial membrane, where it is cleaved to pregnenolone and is therefore indispensable and rate limiting for the synthesis of steroids. Transduction of SF1, indeed, induced the expression of *STAR* (Figure 1B).

Other TFs are involved in adrenocortical development and self-renewal, chiefly PBX1, DAX1, WT1, and CITED2 (Yates et al., 2013). RT-PCR analyses showed that *PBX1*, *DAX1*, and *CITED2*, but not *SF1,* were expressed at the mRNA level in the four cell types before reprogramming, whereas *WT1* was expressed in L-EPCs and BMECs (Figure S2B). Unlike SF1, forced expression of the other TFs either alone (Figure 1B) or in combination did not induce *STAR* expression and neither did they enhance the effect of SF1 alone (Figure 1C). Lentiviral delivery of SF1 did not significantly alter the endogenous expression levels of *PBX1*, *WT1*, *DAX1*, and *CITED2*; however, it reduced the expression of WT1 in L-EPCs (Figure S2B). These data demonstrate that SF1 alone was able to induce *STAR* expression in human cells. Interestingly, on forced expression of SF1, cells (irrespective of the source) had a lower proliferation rate, as assessed by the expression of proliferating cell nuclear antigen (PCNA) and direct cell counting (Figure 1D), and became proliferation arrested three to five days after infection. Cells with this seemingly terminal differentiation phenotype have been maintained for at least two months in culture without loss of viability.

Several treatments were then tested to assess which, if any, would consistently induce an incremental change in the expression of *STAR*. Activators of PKA, such as dibutyryl cyclic AMP (db-cAMP) or 8-bromo-cyclic AMP (8-br-cAMP), were the most potent inducers of *STAR* expression, whereas angiotensin II (AngII), potassium (K^+^), and all-trans retinoic acid (ATRA) had no effect (Figure 1E). Based on these results, 8-br-cAMP (100 μM) was selected to be included in the reprogramming protocol on day two after SF1 transduction.

We next evaluated the dose of SF1 delivered after lentiviral infection and its effect on *STAR* expression: SF1-expressing cells, but not controls, increased *STAR* levels in a multiplicity of infection (MOI)-dependent manner (Figure 1F). Based on these results, an MOI of 200 was used in subsequent experiments.

Morphologically, all cell types similarly underwent a dramatic change of shape 48–72 hr post-transduction, acquiring an irregular stellate morphology with concomitant reduction of the cytoplasmic volume (Figure 1G; Figure S2D; Movie S1). Despite the neuronal-like morphology, absence of mRNA expression of the neuroectoderm marker paired box gene 6 (*PAX6*) (Zhang et al., 2010) and the dopaminergic neuronal marker tyrosine hydroxylase (*TH*) (Lewis et al., 1993) ruled out that these cells could be neuronal-like functionally (Figure S2C).

Electron microscopy images showed larger mitochondria with a densely packed inner mitochondrial membrane in USCs-hiSCs in comparison with controls (Figure 1H). In addition, reprogrammed cells had an increased expression of the mitochondrial import receptor subunit translocase of outer mitochondrial membrane 20 (TOM20) compared with controls, as assessed by western blotting (Figure S2E). Finally, qRT-PCR analyses showed that reprogrammed cells also had enhanced expression of mitochondrial ribosomal RNA 12S (Figure S2F). Together, these data suggest that reprogrammed cells potentially are endowed with higher metabolic activity typical of steroidogenic cells.

### hiSCs Possess Gene Expression Patterns and Functions Specific for Steroidogenic Cells

**Figure 2:**
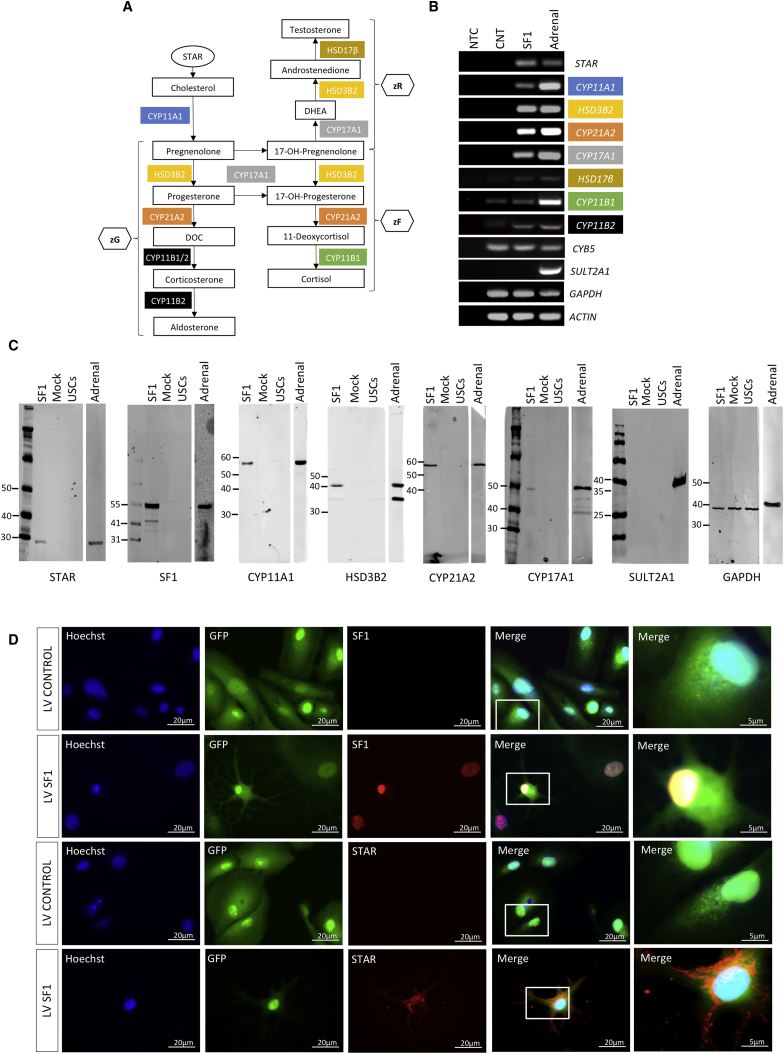
Gene Expression Profile of Reprogrammed USCs (A) Steroidogenic pathway in the adrenal cortex leading to the production of cortisol and aldosterone. (B) RT-PCR expression analyses of *STAR*, steroidogenic enzymes, *SULT2A1*, *GAPDH*, and *ACTIN* in cells infected with SF1 or control (CNT) lentiviruses after eight days. Human adrenal cDNA was used as a positive control. All cells were treated with 8-br-cAMP. NTC, no template control. (C) Western blot analyses of STAR, SF1, steroidogenic enzymes, SULT2A1, and GAPDH expression in USCs and in USCs infected with SF1 or control lentiviruses (mock) after eight days. Human adrenal lysate was used as a positive control. All cells were treated with 8-br-cAMP. (D) Immunostaining of SF1 (upper panels) and STAR (lower panels) in mock-reprogrammed and reprogrammed USCs. Scale bars, 20 μm; insets, 5 μm. Data in (B) are represented as mean ± SEM, n ≥ 3. See also Figure S3.

After cholesterol transfer, steroid hormones are produced after a cascade involving steroidogenic enzymes and intermediate metabolites (Figure 2A). We next assessed the expression of steroidogenic enzymes by RT-PCR eight days after reprogramming USCs. As shown in Figure 2B, we detected *de novo* expression or upregulation of all steroidogenic enzymes analyzed in hiSCs in comparison to cells infected with control lentiviruses. We found expression of cytochrome B5 (*CYB5*) in both control and reprogrammed cells, while we could not detect expression of sulfotransferase 2A1 (*SULT2A1*), which is in agreement with the absence of DHEA-S secretion (see below). Fibroblasts, L-EPCs, and BMECs showed an identical pattern of steroidogenic enzyme induction (Figure S3A). Concomitant expression of SF1, PBX1, WT1, DAX1, and CITED2 (five TFs) enhanced *CYP17A1* and *HSD3B2* expression, whereas they decreased *CYP21A2* expression and did not change *CYP11A1* expression. Four TFs without SF1 had a negligible effect on the expression of steroidogenic enzymes (Figure S3B). Moreover, the expression of steroidogenic enzymes was enhanced by 8-br-cAMP (Figure S3C) as was the expression of *STAR*. Reprogrammed cells did not express the testis marker doublesex and mab-3-related transcription factor 1 (*DMRT1*) and expressed the ovary marker estrogen receptor alpha (*ESR1*) at very low levels (*ESR1* is also expressed in the adrenal gland, although at a lower level compared with the ovaries) (Figure S3D). Moreover, luteinizing hormone receptor (*LHR*) was undetectable in control USCs and was expressed at a very low level in reprogrammed cells compared with the adrenal gland (Figure S3D). These data may suggest a preponderance of adrenocortical-like cells versus a gonadal-like phenotype in reprogrammed cells.

Analyses of hiSCs at the protein level (where specific antibodies were available) confirmed *de novo* expression of STAR, exogenous SF1, steroidogenic enzymes, as well as the absence of SULT2A1, both by western blot (Figure 2C) and immunocytochemistry (Figure 2D; Figure S3E). Differentiation of several independent USC colonies from the same donor or from different donors (either healthy or with congenital adrenal disease) showed similar reprogramming efficiencies as assessed by the expression of STAR protein (Figure S3F), *STAR* mRNA expression, as well as expression of steroidogenic enzymes (data not shown).

**Figure 3:**
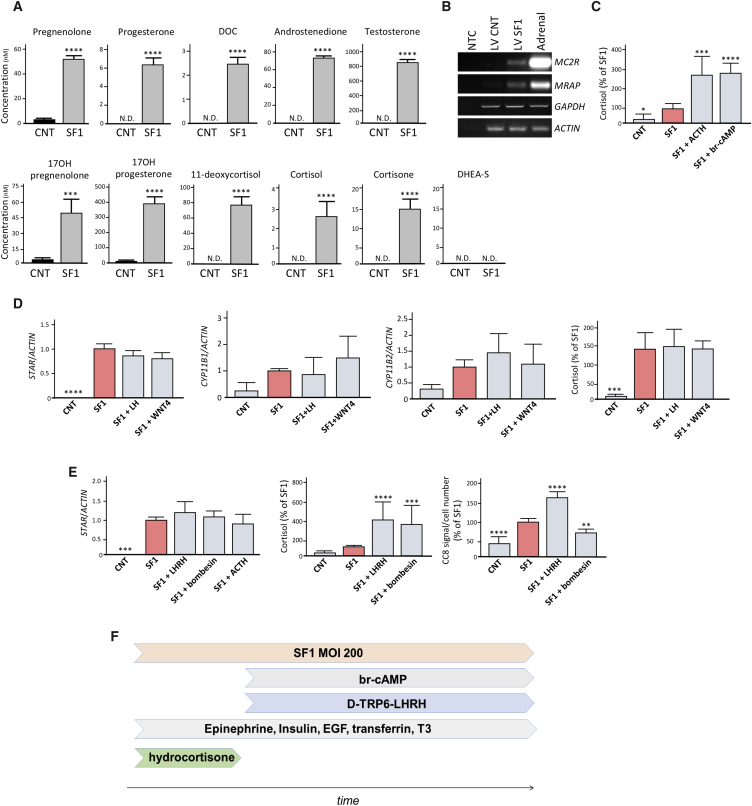
Hormone Production in Reprogrammed USCs (A) LC-MS/MS analyses of steroid in the media of reprogrammed versus control USCs after eight days. N.D., non-detected. (B) RT-PCR showing *MC2R* and *MRAP* expression in controls and reprogrammed USCs after eight days. (C) Cortisol production in control and hiSCs treated with 1 μM ACTH or 100 μM 8-br-cAMP for eight days. (D) qRT-PCR analyses of *STAR*, *CYP11B1*, and *CYP11B2* expression in controls and reprogrammed USCs treated with LH and WNT4 for eight days. Cortisol secretion is reported in the right panel. (E) qRT-PCR analyses of *STAR* expression in USCs treated with LHRH, bombesin, and ACTH. Effect of [D-Trp6]-LHRH and bombesin on cortisol production (left) and in cell viability using CC8 assay. (F) Schematic illustrating the final protocol employed to generate hiSCs. Data in (A) and (C)–(E) are represented as mean ± SEM, n ≥ 3. See also Figure S4.

We next assessed whether urine-derived hiSCs were hormone producing by analyzing cell supernatants of cells eight days after reprogramming using liquid chromatography-tandem mass spectrometry (LC-MS/MS). As shown in Figure 3A, a very significant increase or *de novo* production of steroid hormones and precursors was observed in the medium in hiSCs versus control cells. Dehydroepiandrosterone sulfate (DHEA-S) was not detected, which is in keeping with the absence of SULT2A1 (Figures 2B and 2C). hiSCs had a lower and, despite several interventions, irreversible (Figure S4) cortisol:cortisone ratio compared with human serum or medium from adrenocortical carcinoma H295R cells (Vogeser et al., 2001, Xing et al., 2011).

Overall, these results showed that hiSCs secrete a full repertoire of adrenocortical hormones, irrespective of the cell source, as hiSCs from fibroblasts and L-EPCs had a similar steroid profile (data not shown).

ACTH is the main stimulator of cortisol release in adult adrenal glands through binding to its receptor MC2R, leading to an activation of PKA signaling (Ruggiero and Lalli, 2016). Gene expression analyses for *MC2R* and its accessory protein *MRAP* detected both transcripts in hiSCs; these transcripts were absent in controls (Figure 3B). Cortisol was undetectable in primary cultures before reprogramming or in mock-reprogrammed cells and was produced at low levels in untreated hiSCs; however, a significant and comparable increase was detected after stimulation with ACTH or 8-br-cAMP (Figure 3C). These results demonstrated the functionality of hiSCs in regards to their responsiveness to external stimuli, both physiological (ACTH) and pharmacological (8-br-cAMP).

Recently, β-catenin activation has been shown to promote a zona glomerulosa phenotype; moreover, the action of β-catenin was counteracted by PKA activation, which instead promoted a zona fasciculata phenotype (Drelon et al., 2016). To assess whether the activation of β-catenin had any effect on the expression of zonal-specific markers, reprogrammed cells were treated with recombinant WNT4 (alongside recombinant LH). We found no significant changes in the expression of *CYP11B1*, *CYP11B2*, and *STAR* or on cortisol secretion (Figure 3D). Finally, it has been reported that the LHRH analog [D-Trp6]-LHRH (Balyura et al., 2015) and bombesin (Malendowicz et al., 1995) acutely stimulate adrenal glucocorticoid release. Both LHRH and bombesin, while not affecting *STAR* mRNA levels (Figure 3E), significantly increased cortisol production in hiSCs; however, only LHRH significantly increased the metabolic activity and lifespan of hiSCs in culture (Figure 3E).

Therefore, in our final reprogramming protocol to generate hiSCs, schematized in Figure 3F, [D-Trp6]-LHRH was included at a concentration of 1 μM starting from day two after SF1 infection.

### hiSCs Are Viable When Transplanted into the Mouse Adrenal Gland or Kidney Capsule

**Figure 4:**
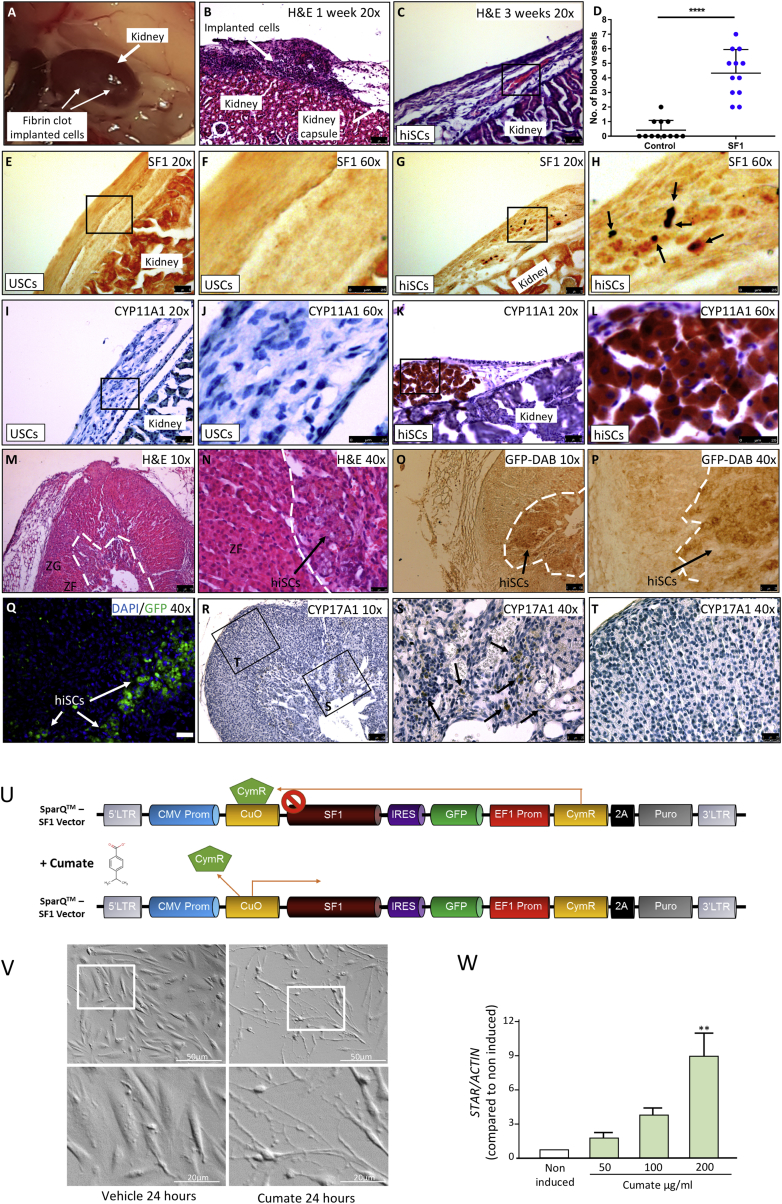
Transplantation of hiSCs into Mouse Kidney and Adrenal Gland (A) hiSCs implanted directly under the kidney capsule as a fibrin clot. (B–D) H&E analyses of cells directly implanted under the kidney capsule as a fibrin clot. Blood vessels were observed to develop in explants after three weeks when hiSCs were implanted directly under the kidney capsule as a fibrin clot (C, count in D). (E–H) Expression of SF1 in controls (E and F) and hiSCs (G and H) implanted directly under the kidney capsule. (I–L) Expression of CYP11A1 in controls (I and J) and hiSCs (K and L) implanted directly under the kidney capsule. (M and N) H&E analyses of a mouse adrenal gland transplanted for one week with USCs infected with SF1 24 hr earlier at lower (M) and higher (N) magnification. hiSCs can be observed at the cortex/medulla boundary (arrow in N). (G–T) Cells transplanted into the adrenal can be visualized by the expression of GFP with immunohistochemistry (O and P), GFP indirect immunofluorescence (Q), or by their expression of CYP17A1 via immunohistochemistry (R–T). Arrows in (O)–(Q) and (S) point to transplanted cells. CYP17A1 is absent in mouse adrenal cortex (R and T). ZG, zona glomerulosa; ZF, zona fasciculata. Scale bars: 50 μm in (B), (C), (E), (G), (I), and (K); 25 μm in (F), (H), (J), (L), (N), (P), (Q), (S), (T); and 100 μm (M), (O), (R). (U–W) Development of an inducible system aimed at generating unlimited amounts of hiSCs. (U) Schematic of the cumate vector generated to infect USCs. In repressed configuration, CymR repressor strongly binds to the cumate operator site (CuO), downstream of the CMV5 promoter. When cumate is present, CymR is released, which enables transgene expression. (V) USCs were infected with the cumate vector, selected with puromycin, and then treated with cumate or vehicle. Cells underwent similar morphologic changes to those observed in Figure 1G. Scale bars, 50 μm; inset, 20 μm. (W) qRT-PCR analyses of *STAR* expression after seven days of treatment with increasing concentration of cumate, showing induction dose dependently. Data in (W) are represented as mean ± SEM, n = 3. See also Figure S5.

To test cell viability *in vivo*, hiSCs from USCs were implanted in mice using three different procedures, schematized in Figure S5A: first, via ectopic implantation under the kidney capsule as free cells with fibrinogen/thrombin mixture; second, as orthotopic (intra-adrenal) implants with fibrinogen/thrombin mixture; and third, via ectopic implantation under the kidney capsule of cells embedded into alginate. No signs of necrosis or apoptosis were observed when cells were directly implanted under the kidney capsule and analyzed after one and three weeks (Figures 4A–4C). In implanted cells, nuclear SF1 staining and expression of steroidogenic enzymes, such as CYP11A1, were observed in hiSCs, but not in control cells (Figures 4E–4L). Interestingly, at three weeks post-transplantation, blood vessels could be detected in hiSC, but not control, xenografts (Figure 4C, quantification in Figure 4D), suggesting neovascularization.

To assess differentiation *in vivo*, cells were implanted orthotopically 24 hr post-infection, at a time when steroidogenic enzymes are not expressed at either the mRNA or the protein level (data not shown); 8-br-cAMP and LHRH were also omitted. Histological analyses of adrenal gland explants after one week, showed no signs of fibrosis or inflammation (Figures 4M and 4N). To localize transplanted cells in the context of host adrenocortical cells, sections were immunostained with a GFP antibody (Figures 4O–4Q); the steroidogenic potential and *in vivo* differentiation of hiSCs was further assessed by their expression of CYP17A1, an enzyme that is epigenetically silenced in adult mice (Missaghian et al., 2009); we observed CYP17A1 staining in transplanted cells, but not in mouse adrenocortical cells (Figures 4R–4T).

hiSCs embedded in alginate and cultured *in vitro* had the same gene expression profile and hormonal secretion as monolayer hiSCs (not shown); however, histological analyses of explants (after one week and three weeks) showed features of anuclear necrosis in most transplanted cells with evident signs of karyolysis (nuclear fading) and karyorrhexis (nuclear fragmentation) (Figure S5B). The same was observed in alginate slabs embedded with control cells (data not shown). These experiments demonstrate the viability of hiSCs in two out of three experimental settings and pave the way for transplantation experiments on a larger scale to assess hiSCs function in murine models of AI, in which a much greater number of reprogrammed cells are needed. However, hiSCs generated from all cell sources were proliferation arrested (Figure 1D), and although we have been unable to find factors promoting proliferation concomitantly with the maintenance of a functional steroidogenic phenotype, a cumate-inducible system was successfully developed, allowing us to generate expandable populations of hiSCs (Figures 4U–4W).

### hiSCs from CAH Patients Show Impaired Steroidogenesis, and Their Hypocortisolism Can Be Reversed through Restoration of Enzymatic Function

**Figure 5:**
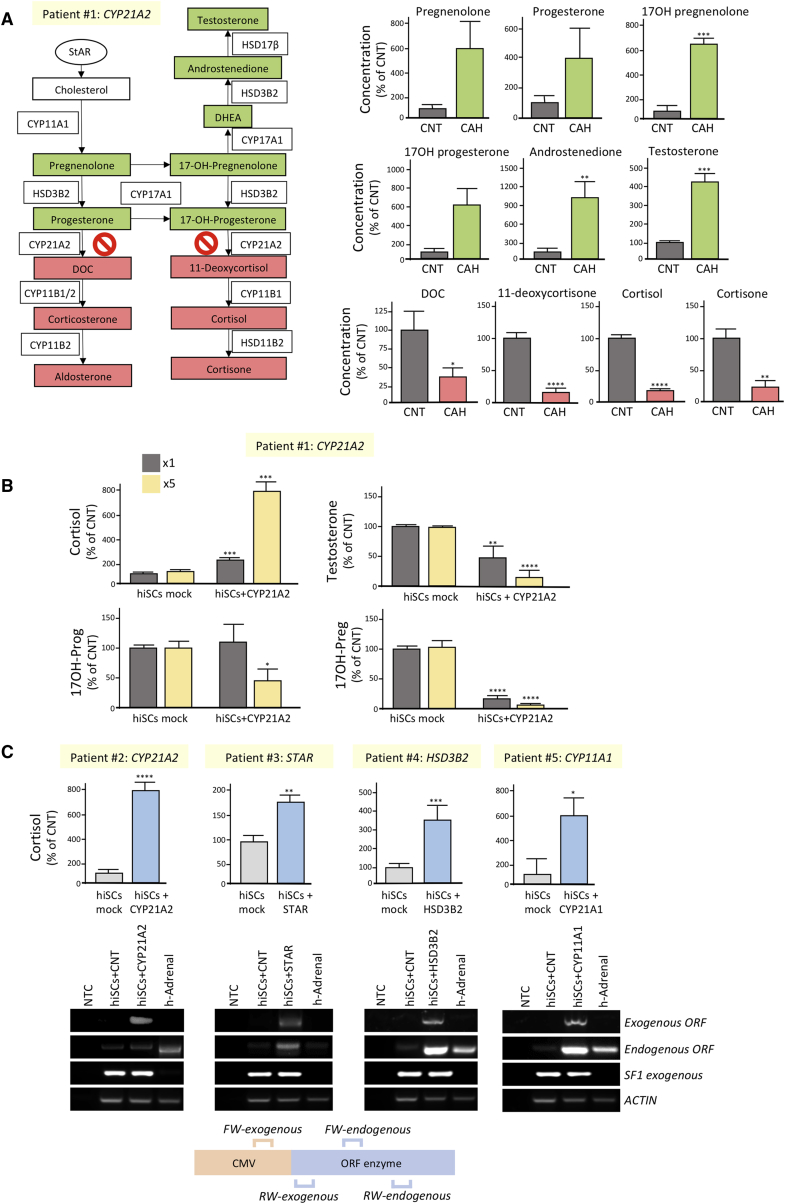
Characterization of Urine-Derived hiSCs Established from Patients with CAH (A) Comparison of steroidogenic profile of hiSCs established from patient #1 with *CYP21A2* mutation (CAH) versus healthy donors (CNT). The diagram on the left shows the steroidogenic pathway with increased metabolites in patient #1 highlighted in green and decreased ones in red. (B) Comparison of cortisol, 17-hydroxyprogesterone, 17-hydroxypregnenolone, and testosterone levels of hiSCs derived from patient #1 with or without restoration of the wild-type form 21-OH. Cells were infected with two increasing amounts of lentiviral particles (×1 and ×5). (C) Comparison of cortisol levels of hiSCs derived from patients (#2–#5) with several forms of CAH with or without restoration of the wild-type form of the corresponding steroidogenic enzymes. RT-PCR analyses using primers encompassing coding- and vector-specific regions confirmed the expression of the exogenous enzymes (lower panels). See also Table 1. Data are represented as mean ± SEM, n ≥ 3.

Currently available immortalized cell lines (mainly obtained from adrenocortical carcinomas) express certain steroidogenic enzymes at low levels, produce an incomplete steroid profile, and have genetic backgrounds specific for the donors they were derived from. Although animal models of CAH have been generated (Bornstein et al., 1999, Mullins et al., 2009), it has not been possible to design disease models involving human steroidogenic cells. For this reason, we evaluated the steroid profile of hiSCs obtained from patients with CAH (Table 1). CAH due to 21-hydroxylase deficiency (21-OH) is a common autosomal recessive disorder caused by defects in the *CYP21A2* gene. Patient #1 harbored one of the most common mutations (p.I172N), resulting in a simple virilizing form of CAH and a residual activity of the enzyme of 1%–10% (Hsu et al., 1996). LC-MS/MS steroid analyses of parallel differentiations of two independent USCs colonies obtained from patient #1 showed an accumulation of metabolites upstream of the 21-OH enzyme, including 17 hydroxyprogesterone, 17 hydroxypregnenolone, and adrenal androgens and a reduction of those downstream, including DOC and cortisol, as compared with healthy donor controls (Figure 5A). Lentiviral delivery of wild-type *CYP21A2* significantly increased cortisol levels in a dose-dependent manner concomitantly to a reduction of 17-hydroxyprogesterone, 17-hydroxypregnenolone, and testosterone (patient #1 in Figure 5B). Furthermore, rescue of cortisol hypo-secretion was successfully achieved in hiSCs established from other CAH patients with diverse genetic mutations (Table 1; Figure 5C). These results demonstrated that hiSCs derived from patients’ urine are also excellent experimental models for monogenic congenital adrenal disorders and are amenable to personalized interventions for treatment.

**Table 1:** Congenital Adrenal Hyperplasia Patients Enrolled in this Study

Patient/Donor Identification	Gene Affected	DNA	Protein
Patient #1	*CYP21A2*	c.515T > A	p.(Ile172Asn)
Patient #2	*CYP21A2*	c.955C > T	p.(Gln319Stop)
Patient #3	*STAR*	c.666delC	p.(Thr223Leufs^∗^98)
Patient #4	*HSD3B2*	NA	NA
Patient #5	*CYP11A1*	c.940G > A	p.(Glu314Lys)
		c.990G > A	

Patient #1 presents a simple virilizing form of CAH due to one of the most common mutations in CYP21A2 (p.I172N). Patient #2 harbors a premature stop codon in *CYP21A2* at glutamine 319 (p.Q319^∗^), which has been previously described as pathogenic. Patient #3 harbors a frameshift mutation in the STAR protein (p. Thr223fs), leading to disruption of the C-terminal START domain responsible for cholesterol binding and promotion of the translocation of cholesterol to the mitochondrial inner membrane. Patient #4 was diagnosed with HSD3B2 deficiency biochemically. Patient #5 is compound heterozygous for two putative splicing mutations in the *CYP11A1* gene, which result in exon 5 skipping and a subsequent frameshift (L.A.M., unpublished data).

## Discussion

Donor- and disease-specific steroidogenic cells as surrogates for disease modeling have been lacking up to now. More significantly, their use could be exploited for the development of cell-based treatment modalities for AI.

We have shown that the use of a single cell fate regulator (SF1/*NR5A1*), in conjunction with PKA and LHRH signaling, can stably reprogram human adult skin-, blood-, and urine-derived cells into hiSCs. Forced expression of other key TFs involved in adrenogonadal differentiation, alone or in combination, was not sufficient to induce hiSCs, nor did their expression with SF1 enhance reprogramming. However, given that there is endogenous expression of DAX1, PBX1, CITED2, and WT1 in non-reprogrammed cells, it is entirely possible that they participate in hiSC induction along with SF1, although the higher dosages delivered by our constructs did not improve reprogramming. Activation of the WNT pathway through WNT4 is associated with zona glomerulosa differentiation, which is prevented by PKA activation (Drelon et al., 2016). Treatment of hiSCs with recombinant WNT4 did not result in changes of zonal-specific markers nor cortisol secretion; it is possible that, in our experimental setting, forced-expression of SF1 bypasses key differentiation events occurring physiologically at the capsule/subcapsular region during the normal self-renewal/zonal specification of the gland.

Fibroblasts are the preferred cell substrate for reprogramming, but more recently, alternative cell types have also been used; USCs are highly expandable, have self-renewal capacity, paracrine properties, and multi-differentiation potential (Bharadwaj et al., 2013, Guan et al., 2014) and have been used as substrates for iPSC generation (Zhou et al., 2012). USC isolation is easy and efficient; we were able to establish cultures with 75% efficacy for one sample and 95% for a second sample, compared with 30% efficacy for L-EPCs (100% for fibroblasts). USCs, L-EPCs, and fibroblasts are all mesoderm derived, however, only USCs are thought to originate from the intermediate mesoderm, a precursor of the adrenocortical and nephritic primordium, which make them an ideal substrate to generate mesodermal tissues, such as adrenogonadal-like cells (Mazilu and McCabe, 2011b). Given the importance of SF1 dosage during adrenal versus gonadal specification (Bland et al., 2004, Val et al., 2007), further development of inducible and tunable systems to modulate the expression of SF1 would likely facilitate a more specific adrenocortical or gonadal induction during reprogramming. Gene expression analyses of adrenocortical and gonadal markers already suggested that our protocol might favor a more adrenocortical phenotype (Figure S3D).

The functionality of hiSCs makes them an unmatched tool to obtain surrogate adrenocortical cells for *in vitro* disease modeling. With this in mind, we have derived hiSCs from patients with CAH, showing an altered steroid profile. Importantly, the decrease in cortisol production in hiSCs derived from CAH patients was rescued on expression of the exogenous native forms, irrespective of the defective steroidogenic enzyme.

USCs-hiSCs were shown to be viable *in vivo* when transplanted into mouse adrenal gland tissue and successfully differentiate toward an adrenal-like lineage within the adrenal tissue itself, as cells were implanted 24 hr post-infection when steroidogenic enzymes are not expressed. Whether the surrounding adrenal tissue favors differentiation remains to be explored, as well as the long-term viability/functionality and fate of transplanted cells. hiSCs implanted under the mouse kidney capsule maintained the expression of steroidogenic enzymes and showed well-vascularized tissue at the site of transplantation after three weeks, suggesting viability of those cells *in vivo*. Remarkably, vascularization was not observed in control implants; this can be explained either by the intrinsic ability of SF1 to regulate adrenocortical vascular remodeling through the expression of angiopoietin-2 (Ferraz-de-Souza et al., 2011) or by the very significant upregulation of the potent pro-angiogenic tetraspartin CD9 in hiSCs detected in a proteomic array in all cell sources (Figure S6). CD9, also expressed in the normal adrenal, but with unknown function (Nakamura et al., 2001), exerts its angiogenic action via exosomes, where it is abundantly expressed (Andreu and Yáñez-Mó, 2014).

Sodium alginate has been used as a 3D scaffold for cell immobilization/delivery in tissue engineering (Bidarra et al., 2014) and might confer some immunoprotection (Llacua et al., 2016). However, hiSC viability was compromised on implantation; control cells were also negatively affected, suggesting cell-specific toxicity of alginate after grafting in our system (both hiSCs and control cells embedded in alginate were viable and functional *in vitro*).

Overall, these pilot transplantation experiments showed that viability of hiSCs is affected by the transplantation protocol used. Longer-term experiments with a yet-to-be determined amount of cells would allow for careful assessment of: (1) the number of cells needed to be able to detect cortisol (undetectable in our pilot experiments); (2) the time needed to become fully functional; (3) their viability long term; and (4) their rescuing potential in animal models of AI.

Novel approaches to treating AI are needed for a condition in which mortality rates continue to be 2-fold higher than the background population despite steroid replacement (Bergthorsdottir et al., 2006). Although newer steroid formulations (e.g., Plenadren and Chronocort) are being developed to mimic circadian rhythms more precisely, replacement remains inherently limited in its responsiveness to physiological need. This study paves the way for the development of alternative approaches to treat AI. For example, hiSCs could be implanted intra-adrenally or encapsulated using immunoisolating chambers, possibly after gene-editing in case of mono/oligogenic disorders, as previously used in beta-cell transplantation (Desai and Shea, 2017). To this end, USCs have recently been successfully gene edited in a model of human muscle disease (Kim et al., 2016). This will allow a curative cell replacement therapy for patients with AI.

## Experimental Procedures

Further details and an outline of resources used in this work can be found in Supplemental Experimental Procedures.

### Materials

All antibodies, plasmids, and primers used are listed in Supplemental Experimental Procedures.

### Cell Culture

Primary cultures of USCs, fibroblasts, and L-EPCs were isolated as previously described (Martin-Ramirez et al., 2012, Poliandri et al., 2017, Zhou et al., 2012). BMECs were a kind gift of Dr. Egle Solito (Schweitzer et al., 1997, Solito et al., 2000), and HEK293T lines were obtained from ATCC. SF1-inducible cell lines were generated using a SparQ cumate switch pCDH-CuO-MCS-IRES-GFP-EF1-CymR-T2A-Puro All-in-one inducible lentivector (Cambridge Bioscience, Ltd.) expressing SF1. The detailed protocols for the isolation of all cell types are provided in Supplemental Experimental Procedures.

### Lentiviral Production and Cell Reprogramming

Lentiviral particles were obtained as previously described (Cribbs et al., 2013) with minor modifications. 60,000 cells per well of a six-well plate were infected with lentiviral particles at a MOI of 200 with 8 μg/mL of polybrene (Millipore, TR-1003-G). The medium was replaced after 12 hr, and treatments with different molecules were started after two days. Cells were cultured for an additional 5–10 days before analysis. Drug concentrations are detailed in Supplemental Experimental Procedures.

### Gene Expression Analysis

See Supplemental Experimental Procedures for details.

### Immunoassays

Immunoblotting, immunocytochemistry, and immunohistochemistry were performed as described previously (Guasti et al., 2011, Rodríguez-Asiain et al., 2011). See Supplemental Experimental Procedures for details.

### Hormone Quantification

Hormone quantification was performed using an ELISA Kit (Abcam, ab154996) according to the manufacturer’s instructions and LC-MS/MS (Taylor et al., 2017). See Supplemental Experimental Procedures for details.

### Mitochondrial Morphology Studies

Electron microscopy was performed by the Nanovision Centre, Queen Mary University of London, using standardized procedures.

### Animal Experiments

All animal experiments were performed in strict accordance with protocols approved by the ethical board of Landesdirektion Sachsen, Germany (protocol no. DD24-5131/354/28). Female 8-week-old C57BL/6 mice were obtained from Charles River Laboratory. Two million cells were transplanted under the kidney capsule and 5 × 10^5^ cells concentrated in a total volume of 10 μL were pipetted directly into the adrenal gland.

See Supplemental Experimental Procedures for details.

### Collection of Urine Samples from Donors

This study has been performed under the ethical approval of the National Health Service, Research Ethic Committee (NHS REC; reference: 13/LO/0224). All patients involved were previously informed, and consent forms were obtained before analysis of samples.

### Protein Array of Human Reprogrammed Steroidogenic Cells

Protein array was performed by Sciomics, GmbH (Germany) using control and eight-day reprogrammed hiSCs. See Supplemental Experimental Procedures for details.

### Data Analysis

Figures and tables were generated using Microsoft Excel, ImageJ, and Adobe Photoshop.

### Statistical Analysis

Statistical analysis was performed using GraphPad Prism 7, and statistical significance was determined using one-way ANOVA followed by Dunnet’s multiple comparison test correction. A Student’s t test was performed when only two means were compared. Significance: not significant (ns) p > 0.05; ^∗^p < 0.05; ^∗∗^p < 0.01; ^∗∗∗^p < 0.001; ^∗∗∗∗^p < 0.0001.

## Data and Software Availability

The accession number for the protein array data reported in this article is Mendeley Data: https://doi.org/10.17632/x9rn9kcr3b.2.

## Supplemental Information

Document S1. Supplemental Experimental Procedures and Figures S1–S6

Movie S1. Morphologic Changes of USCs Post-SF1-Infection in a 48-hr Time-Lapse, Related to Figure 1

Document S2. Article plus Supplemental Information
