# Supplementary material for: Modeling Congenital Adrenal Hyperplasia and Testing Interventions for Adrenal Insufficiency Using Donor-Specific Reprogrammed Cells
Source: Cell Rep. 2018 Jan 30;22(5):1236–49. doi: 10.1016/j.celrep.2018.01.003 (PMC5809617; doi:10.1016/j.celrep.2018.01.003)
Supplement: Document S2. Article plus Supplemental Information [file mmc3.pdf]

Cell Reports

Article

Modeling Congenital Adrenal Hyperplasia and Testing Interventions for Adrenal Insufficiency Using Donor-Specific Reprogrammed Cells

Graphical Abstract

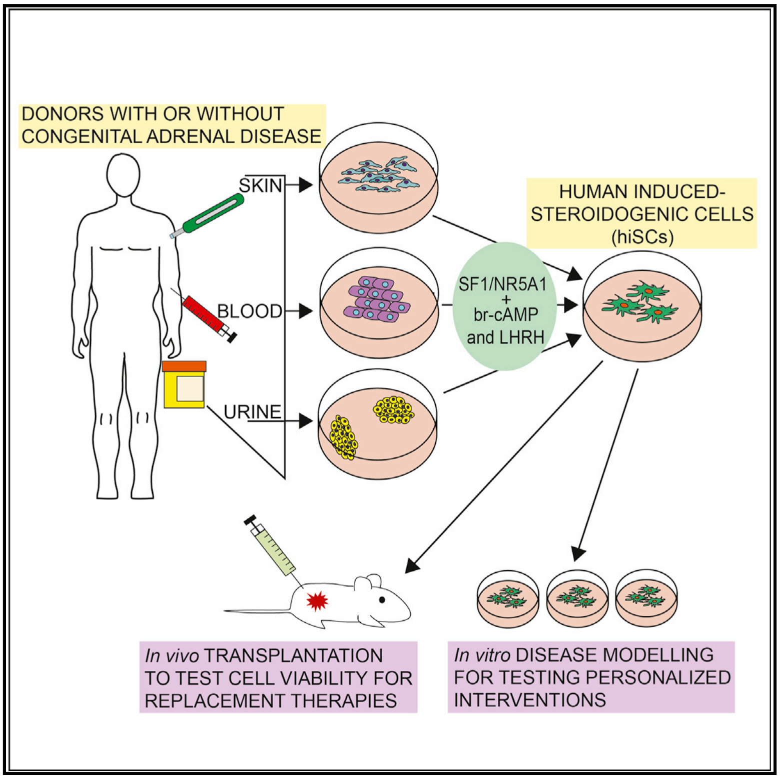

Highlights

- Induced steroidogenic cells (hiSCs) can be derived from urine, skin, and blood cells
- hiSCs express steroidogenic enzymes and secrete cortisol in a stimulus-dependent manner
- hiSCs are viable *in vivo* after intra-adrenal or kidney capsule transplantation
- hiSCs can be used to model adrenal disorders and potentially for cell-based therapies

Authors

Gerard Ruiz-Babot, Mariya Balyura,
Irene Hadjidemetriou, ...,
Louise A. Metherell, Stefan R. Bornstein,
Leonardo Guasti

Correspondence

l.guasti@qmul.ac.uk

In Brief

Ruiz-Babot et al. generate functional human steroidogenic cells (hiSCs), which are responsive to both pharmacological and physiological stimuli. Moreover, the hypocortisolism in hiSCs derived from patients with congenital adrenal hyperplasia is restored to normal through the incorporation of the wild-type version of the defective disease-causing enzymes.

Ruiz-Babot et al., 2018, Cell Reports *22*, 1236–1249
January 30, 2018 © 2018 The Authors.
https://doi.org/10.1016/j.celrep.2018.01.003

CellPress

OPEN ACCESS
CellPress

Cell Reports
Article

Modeling Congenital Adrenal Hyperplasia and Testing Interventions for Adrenal Insufficiency Using Donor-Specific Reprogrammed Cells

Gerard Ruiz-Babot,[1] Mariya Balyura,[2] Irene Hadjidemetriou,[1] Sharon J. Ajodha,[1] David R. Taylor,[3] Lea Ghataore,[3] Norman F. Taylor,[3] Undine Schubert,[2] Christian G. Ziegler,[2] Helen L. Storr,[1] Maralyn R. Druce,[1] Evelien F. Gevers,[1] William M. Drake,[1] Umasuthan Srirangalingam,[4] Gerard S. Conway,[4] Peter J. King,[1] Louise A. Metherell,[1] Stefan R. Bornstein,[2,5,6,7] and Leonardo Guasti[1,8,*]

[1]Centre for Endocrinology, William Harvey Research Institute, Barts and the London School of Medicine and Dentistry, Queen Mary University of London, EC1M 6BQ London, UK
[2]University Hospital Carl Gustav Carus, Department of Medicine III, Technische Universität Dresden, 01307 Dresden, Germany
[3]Department of Clinical Biochemistry, King's College Hospital NHS Foundation Trust, Denmark Hill, SE5 9RS London, UK
[4]Department of Endocrinology, University College London Hospitals, NW1 2PG London, UK
[5]Paul Langerhans Institute Dresden of Helmholtz Centre Munich at University Clinic Carl Gustav Carus of TU Dresden Faculty of Medicine, Technische Universität Dresden, DZD-German Centre for Diabetes Research, 01307 Dresden, Germany
[6]Center for Regenerative Therapies, Technische Universität Dresden, 01307 Dresden, Germany
[7]Diabetes and Nutritional Sciences Division, King's College London, WC2R 2LS London, UK
[8]Lead Contact
*Correspondence: l.guasti@qmul.ac.uk
https://doi.org/10.1016/j.celrep.2018.01.003

SUMMARY

Adrenal insufficiency is managed by hormone replacement therapy, which is far from optimal; the ability to generate functional steroidogenic cells would offer a unique opportunity for a curative approach to restoring the complex feedback regulation of the hypothalamic-pituitary-adrenal axis. Here, we generated human induced steroidogenic cells (hiSCs) from fibroblasts, blood-, and urine-derived cells through forced expression of steroidogenic factor-1 and activation of the PKA and LHRH pathways. hiSCs had ultrastructural features resembling steroid-secreting cells, expressed steroidogenic enzymes, and secreted steroid hormones in response to stimuli. hiSCs were viable when transplanted into the mouse kidney capsule and intra-adrenal. Importantly, the hypocortisolism of hiSCs derived from patients with adrenal insufficiency due to congenital adrenal hyperplasia was rescued by expressing the wild-type version of the defective disease-causing enzymes. Our study provides an effective tool with many potential applications for studying adrenal pathobiology in a personalized manner and opens venues for the development of precision therapies.

INTRODUCTION

The adrenal cortex is a major steroid-producing organ, secreting glucocorticoids under the control of adrenocorticotropic hormone (ACTH), secreted by the anterior pituitary gland, and mineralocorticoids under the control of the renin-angiotensin system. Glucocorticoids affect carbohydrate metabolism and mediate the mammalian stress response, and mineralocorticoids control blood volume and salt homeostasis. Primary or secondary adrenal insufficiency (AI) results from adrenal failure or impairment of the hypothalamic-pituitary axis, respectively. In both cases, the cortex fails to secrete sufficient amounts of glucocorticoids and adrenal androgens, but in primary AI, the clinical consequences of aldosterone deficiency make this a more lethal condition. The most frequent cause of primary AI is autosomal recessive congenital adrenal hyperplasia (CAH), which results from defects in enzymes involved in steroid biosynthesis (Merke and Bornstein, 2005). Patients with AI need life-long management with exogenous steroids: this can be challenging, because no drug suitably mimics the diurnal pattern of cortisol, and objective variables measuring the quality of replacement therapy are lacking. Fine-tuning of replacement therapy leaves only a narrow margin for improvement: under-replacement can result in severe impairment of well-being and incipient crisis, whereas even subtle, chronic over-replacement has the potential to contribute to excess morbidity, including obesity, osteoporosis, hypertension, and impaired glucose tolerance. Therefore, better treatment solutions are urgently needed (Bornstein et al., 2016).

The ability to generate donor-specific and functional adrenocortical-like cells would facilitate: (1) the next generation of cell-based treatments for AI; (2) the modeling of adrenal-specific diseases; and (3) the testing of personalized interventions on cells derived from patients.

Cells with an adrenocortical-like phenotype have never been obtained in a patient-specific manner; moreover, previous attempts have resulted in cells with very limited steroidogenic potential (Crawford et al., 1997) or have used lines of embryonic

 © 2018 The Authors.
This is an open access article under the CC BY license (http://creativecommons.org/licenses/by/4.0/).

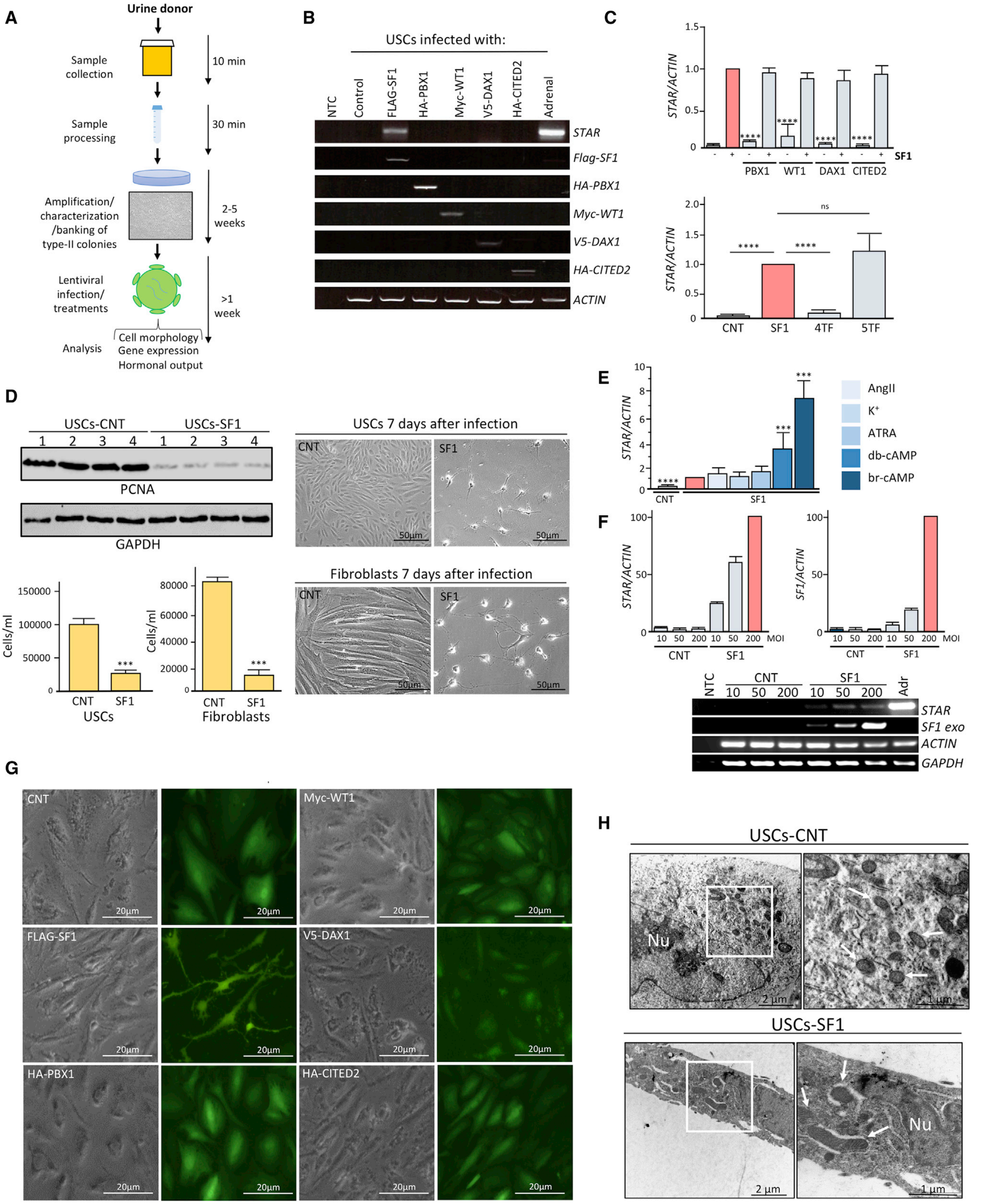

(legend on next page)

stem cells (Jadhav and Jameson, 2011; Yazawa et al., 2011), mesenchymal stem cells (Gondo et al., 2004, 2008; Mazilu and McCabe, 2011a; Tanaka et al., 2007; Wei et al., 2012; Yazawa et al., 2006, 2009, 2010), and induced pluripotent stem cells (iPSCs) (Sonoyama et al., 2012). The prerequisite of all these studies was the forced expression of steroidogenic factor-1 (SF1), a master regulator of adrenogonadal development and function encoded by nuclear receptor subfamily 5, group A, member 1 (*NR5A1*). SF1 is a true effector of cell fate as it initiates a genetic program driving embryonic mesenchymal cells toward a steroidogenic phenotype/lineage (Schimmer and White, 2010), and SF1 mutations can result in adrenal insufficiency (Achermann et al., 2001). Other than SF1, additional transcription factors (TFs), such as wilms tumor 1 (WT1), CBP/p300-interacting transactivator 2 (CITED2), pre-B cell leukemia transcription factor 1 (PBX1), and dosage-sensitive sex reversal, adrenal hypoplasia critical region, on chromosome X, gene 1 (DAX1) have been shown to be key determinants of adrenal cortex development (Yates et al., 2013). Moreover, multiple pathways have been implicated in the fine-tuned regulation of adrenocortical development, zonation, and self-renewal (Lerario et al., 2017).

Here, we show an efficient protocol to reprogram, through stable expression of SF1 and activation of the protein kinase A (PKA) pathway and in the presence of luteinizing-hormone-releasing hormone (LHRH), easily accessible sources of cells from humans (blood, skin, and urine), resulting in human induced steroidogenic cells (hiSCs). These hiSCs expressed steroidogenic enzymes and secreted cortisol in a stimulus-dependent manner. hiSCs could be efficiently exploited to study donor-specific disease pathobiology and to test interventions, such as restoration of eucortisolism in hiSCs from CAH patients. Moreover, as a first step to assess whether hiSCs can be a viable option for the development of cell-based treatments for AI, we performed pilot *in vivo* experiments proving the viability of hiSCs after transplantation into the adrenal glands and under the kidney capsule of mice. These experiments pave the way for further testing of hiSCs in suitable rodent models of AI, such as double adrenalectomised rats (Balyura et al., 2015; Ruiz-Babot et al., 2015).

RESULTS

Establishment of Human Primary Cultures from Different Cell Sources

Primary cultures of human urine-derived stem cells (USCs), late-outgrowth endothelial progenitor cells (L-EPCs), and fibroblasts were initially established from healthy donors (Figure S1). Because L-EPCs are phenotypically indistinguishable from bone-marrow-derived endothelial cells (BMECs) (Yoder et al., 2012), the latter were also used in our experiments.

Generation of hiSCs by Direct Lineage Conversion

Lentiviral vectors encoding SF1 and other TFs (PBX1, DAX1, WT1, and CITED2) were used to infect human primary cells. The vectors co-express GFP bicistronically and contain a mammalian resistance cassette, which was used for selection (Figure S2A). Cells were transduced according to the schematic in Figure 1A and as reported in the Experimental Procedures. The expression of the steroidogenic acute regulatory protein (*STAR*) was used as a readout for initial experiments; STAR mediates the transfer of cholesterol from the outer to the inner mitochondrial membrane, where it is cleaved to pregnenolone and is therefore indispensable and rate limiting for the synthesis of steroids. Transduction of SF1, indeed, induced the expression of *STAR* (Figure 1B).

Other TFs are involved in adrenocortical development and self-renewal, chiefly PBX1, DAX1, WT1, and CITED2 (Yates et al., 2013). RT-PCR analyses showed that *PBX1*, *DAX1*, and *CITED2*, but not *SF1,* were expressed at the mRNA level in the four cell types before reprogramming, whereas *WT1* was expressed in L-EPCs and BMECs (Figure S2B). Unlike SF1, forced expression of the other TFs either alone (Figure 1B) or in combination did not induce *STAR* expression and neither did they enhance the effect of SF1 alone (Figure 1C). Lentiviral delivery of SF1 did not significantly alter the endogenous expression levels of *PBX1*, *WT1*, *DAX1*, and *CITED2*; however, it reduced the expression of WT1 in L-EPCs (Figure S2B). These data demonstrate that SF1 alone was able to induce *STAR* expression in human cells. Interestingly, on forced expression of SF1, cells (irrespective of the source) had a lower proliferation rate, as

Figure 1. Conversion of Human Urine-Derived Stem Cells into Steroidogenic Cells

(A) Schematic illustrating our strategy for urine collection, processing, and reprogramming. Urine-derived cells (USCs) were cultured in specific media, and type-II colonies amplified and characterized through flow cytometry. Then they were either banked or expanded for experiments. USCs were infected at passage two with either a lentivirus encoding a transcription factor (TF) within an IRES-GFP vector, a combination of TFs, or mock infected (MOI = 200). Cells were treated with 8-br-cAMP (100 μM) unless stated otherwise and kept in culture for at least eight days before analyses.

(B) RT-PCR showing *STAR* expression on forced expression of each TF. The expression of exogenous *SF1*, *PBX1*, *WT1*, *DAX1*, and *CITED2* was assessed by RT-PCR using primers encompassing the coding- and vector- specific regions. Human adrenal cDNA was used as a positive control for endogenous *STAR* expression and, along with non-template control (NTC), as a negative control for exogenous TF expression.

(C) qRT-PCR analyses of *STAR* expression on forced expression of SF1 with each TF (upper panel) and of SF1 with or without a combination of TFs (lower panel).

(D) Western blot analyses of PCNA and GAPDH expression in hiSCs and mock-reprogrammed USCs from four independent donors eight days after reprogramming (top left panels); cell counting (bottom left panels) and representative images (right panels) of hiSCs obtained from USCs and fibroblasts versus mock-reprogrammed cells. Scale bars, 50 μm.

(E) qRT-PCR analyses of *STAR* expression on forced expression of SF1 with or without the indicated treatments, started the day after infection for seven days. CNT, cells infected with empty control vector.

(F) qRT-PCR (upper panel) and RT-PCR (lower panels) analyses of *STAR* and *SF1* expression after reprogramming USCs at different MOI of SF1 or empty control lentiviral vector (CNT).

(G) Morphological changes on SF1 overexpression in USCs eight days post-infection. Scale bars, 20 μm.

(H) Electron microscopy images of USCs and USCs eight days after reprogramming. Arrows point to mitochondria. Nu, nucleus.

Scale bars, 2 μm (left panels) and 1 μm (right panels). Data in (C)–(F) are represented as mean ± SEM, n ≥ 3. See also Figures S1 and S2.

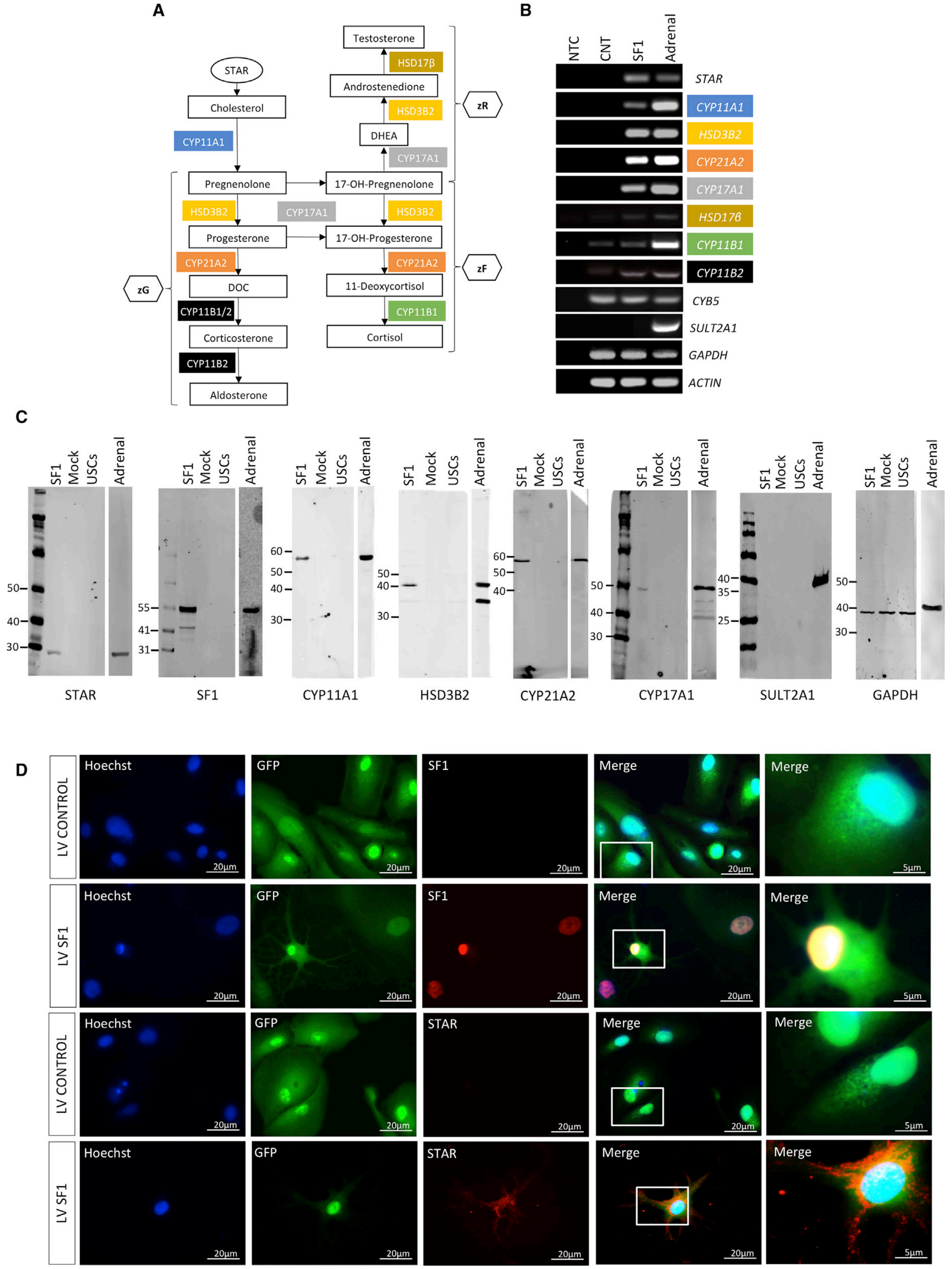

(legend on next page)

assessed by the expression of proliferating cell nuclear antigen (PCNA) and direct cell counting (Figure 1D), and became proliferation arrested three to five days after infection. Cells with this seemingly terminal differentiation phenotype have been maintained for at least two months in culture without loss of viability.

Several treatments were then tested to assess which, if any, would consistently induce an incremental change in the expression of *STAR*. Activators of PKA, such as dibutyryl cyclic AMP (db-cAMP) or 8-bromo-cyclic AMP (8-br-cAMP), were the most potent inducers of *STAR* expression, whereas angiotensin II (AngII), potassium (K^+), and all-trans retinoic acid (ATRA) had no effect (Figure 1E). Based on these results, 8-br-cAMP (100 μM) was selected to be included in the reprogramming protocol on day two after SF1 transduction.

We next evaluated the dose of SF1 delivered after lentiviral infection and its effect on *STAR* expression: SF1-expressing cells, but not controls, increased *STAR* levels in a multiplicity of infection (MOI)-dependent manner (Figure 1F). Based on these results, an MOI of 200 was used in subsequent experiments.

Morphologically, all cell types similarly underwent a dramatic change of shape 48–72 hr post-transduction, acquiring an irregular stellate morphology with concomitant reduction of the cytoplasmic volume (Figure 1G; Figure S2D; Movie S1). Despite the neuronal-like morphology, absence of mRNA expression of the neuroectoderm marker paired box gene 6 (*PAX6*) (Zhang et al., 2010) and the dopaminergic neuronal marker tyrosine hydroxylase (*TH*) (Lewis et al., 1993) ruled out that these cells could be neuronal-like functionally (Figure S2C).

Electron microscopy images showed larger mitochondria with a densely packed inner mitochondrial membrane in USCs-hiSCs in comparison with controls (Figure 1H). In addition, reprogrammed cells had an increased expression of the mitochondrial import receptor subunit translocase of outer mitochondrial membrane 20 (TOM20) compared with controls, as assessed by western blotting (Figure S2E). Finally, qRT-PCR analyses showed that reprogrammed cells also had enhanced expression of mitochondrial ribosomal RNA 12S (Figure S2F). Together, these data suggest that reprogrammed cells potentially are endowed with higher metabolic activity typical of steroidogenic cells.

hiSCs Possess Gene Expression Patterns and Functions Specific for Steroidogenic Cells

After cholesterol transfer, steroid hormones are produced after a cascade involving steroidogenic enzymes and intermediate metabolites (Figure 2A). We next assessed the expression of steroidogenic enzymes by RT-PCR eight days after reprogramming USCs. As shown in Figure 2B, we detected *de novo* expression or upregulation of all steroidogenic enzymes analyzed in hiSCs in comparison to cells infected with control lentiviruses. We found expression of cytochrome B5 (*CYB5*) in both control and reprogrammed cells, while we could not detect expression of sulfotransferase 2A1 (*SULT2A1*), which is in agreement with the absence of DHEA-S secretion (see below). Fibroblasts, L-EPCs, and BMECs showed an identical pattern of steroidogenic enzyme induction (Figure S3A). Concomitant expression of SF1, PBX1, WT1, DAX1, and CITED2 (five TFs) enhanced *CYP17A1* and *HSD3B2* expression, whereas they decreased *CYP21A2* expression and did not change *CYP11A1* expression. Four TFs without SF1 had a negligible effect on the expression of steroidogenic enzymes (Figure S3B). Moreover, the expression of steroidogenic enzymes was enhanced by 8-br-cAMP (Figure S3C) as was the expression of *STAR*. Reprogrammed cells did not express the testis marker doublesex and mab-3-related transcription factor 1 (*DMRT1*) and expressed the ovary marker estrogen receptor alpha (*ESR1*) at very low levels (*ESR1* is also expressed in the adrenal gland, although at a lower level compared with the ovaries) (Figure S3D). Moreover, luteinizing hormone receptor (*LHR*) was undetectable in control USCs and was expressed at a very low level in reprogrammed cells compared with the adrenal gland (Figure S3D). These data may suggest a preponderance of adrenocortical-like cells versus a gonadal-like phenotype in reprogrammed cells.

Analyses of hiSCs at the protein level (where specific antibodies were available) confirmed *de novo* expression of STAR, exogenous SF1, steroidogenic enzymes, as well as the absence of SULT2A1, both by western blot (Figure 2C) and immunocytochemistry (Figure 2D; Figure S3E). Differentiation of several independent USC colonies from the same donor or from different donors (either healthy or with congenital adrenal disease) showed similar reprogramming efficiencies as assessed by the expression of STAR protein (Figure S3F), *STAR* mRNA expression, as well as expression of steroidogenic enzymes (data not shown).

We next assessed whether urine-derived hiSCs were hormone producing by analyzing cell supernatants of cells eight days after reprogramming using liquid chromatography-tandem mass spectrometry (LC-MS/MS). As shown in Figure 3A, a very significant increase or *de novo* production of steroid hormones and precursors was observed in the medium in hiSCs versus control cells. Dehydroepiandrosterone sulfate (DHEA-S) was not detected, which is in keeping with the absence of SULT2A1 (Figures 2B and 2C). hiSCs had a lower and, despite several interventions, irreversible (Figure S4) cortisol:cortisone ratio compared with human serum or medium from adrenocortical carcinoma H295R cells (Vogeser et al., 2001; Xing et al., 2011).

Overall, these results showed that hiSCs secrete a full repertoire of adrenocortical hormones, irrespective of the cell source,

Figure 2. Gene Expression Profile of Reprogrammed USCs

(A) Steroidogenic pathway in the adrenal cortex leading to the production of cortisol and aldosterone.

(B) RT-PCR expression analyses of *STAR*, steroidogenic enzymes, *SULT2A1*, *GAPDH*, and *ACTIN* in cells infected with SF1 or control (CNT) lentiviruses after eight days. Human adrenal cDNA was used as a positive control. All cells were treated with 8-br-cAMP. NTC, no template control.

(C) Western blot analyses of STAR, SF1, steroidogenic enzymes, SULT2A1, and GAPDH expression in USCs and in USCs infected with SF1 or control lentiviruses (mock) after eight days. Human adrenal lysate was used as a positive control. All cells were treated with 8-br-cAMP.

(D) Immunostaining of SF1 (upper panels) and STAR (lower panels) in mock-reprogrammed and reprogrammed USCs. Scale bars, 20 μm; insets, 5 μm.

Data in (B) are represented as mean ± SEM, n ≥ 3. See also Figure S3.

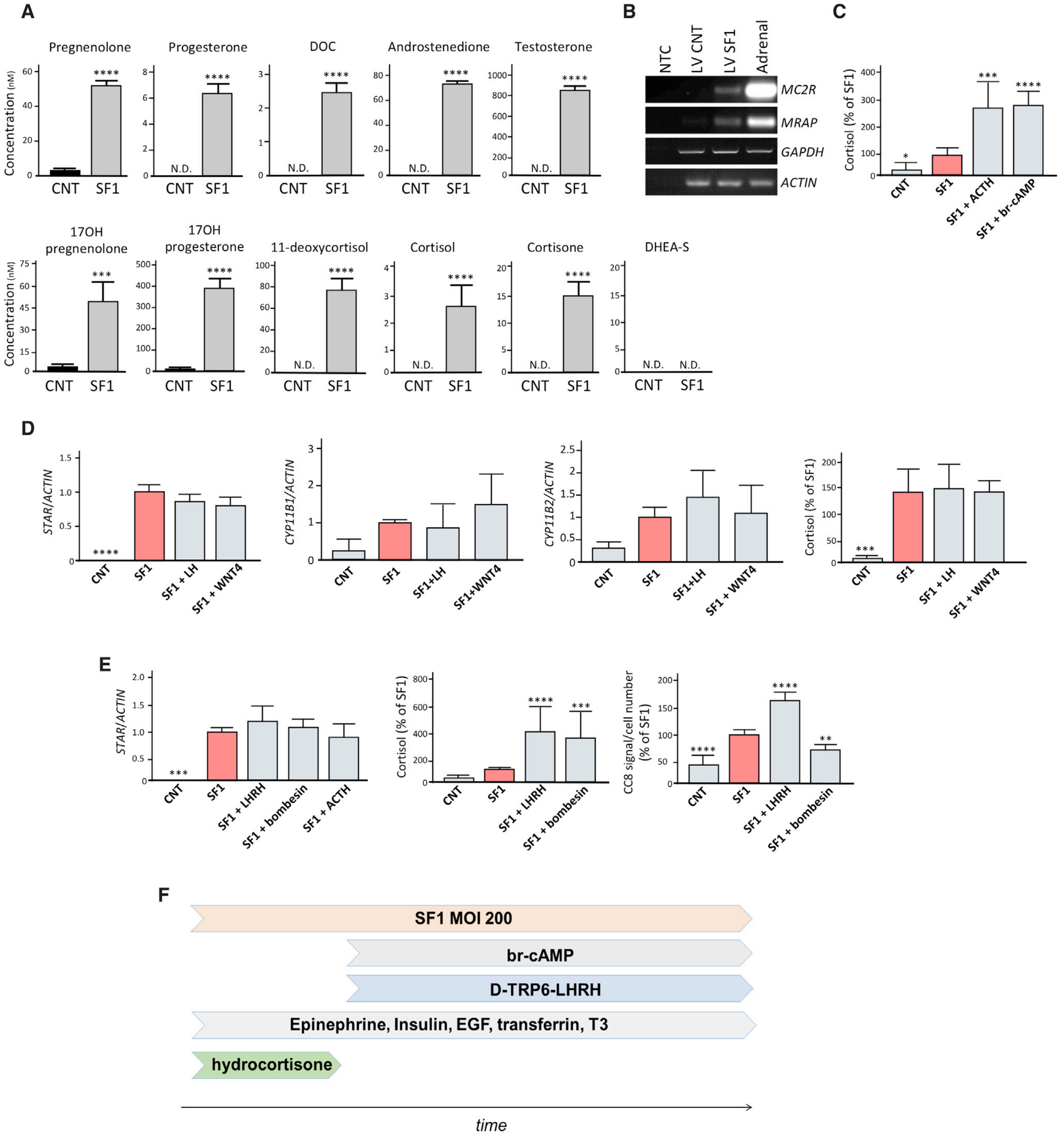

Figure 3. Hormone Production in Reprogrammed USCs

(A) LC-MS/MS analyses of steroid in the media of reprogrammed versus control USCs after eight days. N.D., non-detected.

(B) RT-PCR showing *MC2R* and *MRAP* expression in controls and reprogrammed USCs after eight days.

(C) Cortisol production in control and hiSCs treated with 1 μM ACTH or 100 μM 8-br-cAMP for eight days.

(D) qRT-PCR analyses of *STAR*, *CYP11B1*, and *CYP11B2* expression in controls and reprogrammed USCs treated with LH and WNT4 for eight days. Cortisol secretion is reported in the right panel.

(E) qRT-PCR analyses of *STAR* expression in USCs treated with LHRH, bombesin, and ACTH. Effect of [D-Trp6]-LHRH and bombesin on cortisol production (left) and in cell viability using CC8 assay.

(F) Schematic illustrating the final protocol employed to generate hiSCs.

Data in (A) and (C)–(E) are represented as mean ± SEM, n ≥ 3. See also Figure S4.

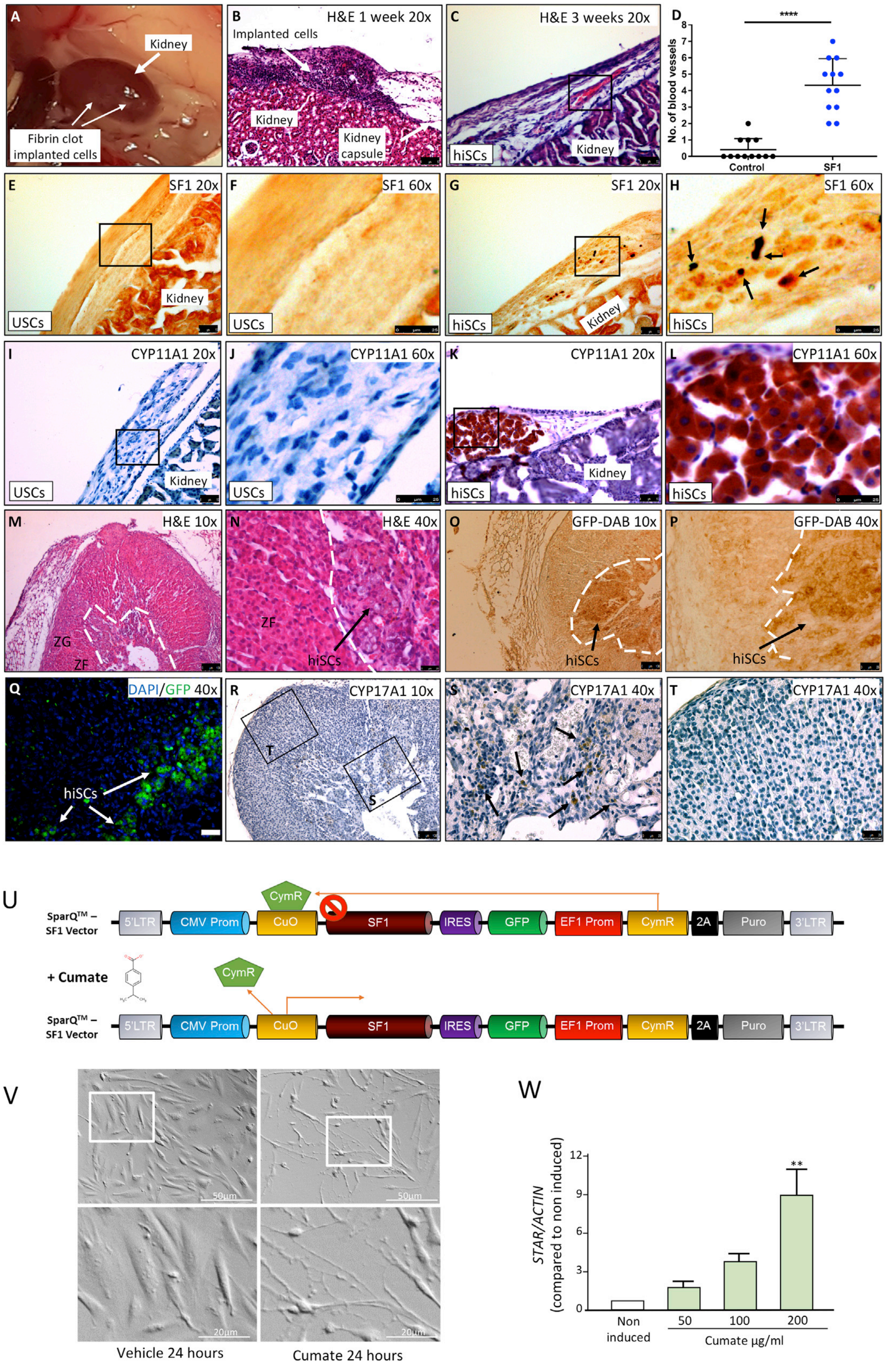

(legend on next page)

as hiSCs from fibroblasts and L-EPCs had a similar steroid profile (data not shown).

ACTH is the main stimulator of cortisol release in adult adrenal glands through binding to its receptor MC2R, leading to an activation of PKA signaling (Ruggiero and Lalli, 2016). Gene expression analyses for *MC2R* and its accessory protein *MRAP* detected both transcripts in hiSCs; these transcripts were absent in controls (Figure 3B). Cortisol was undetectable in primary cultures before reprogramming or in mock-reprogrammed cells and was produced at low levels in untreated hiSCs; however, a significant and comparable increase was detected after stimulation with ACTH or 8-br-cAMP (Figure 3C). These results demonstrated the functionality of hiSCs in regards to their responsiveness to external stimuli, both physiological (ACTH) and pharmacological (8-br-cAMP).

Recently, β-catenin activation has been shown to promote a zona glomerulosa phenotype; moreover, the action of β-catenin was counteracted by PKA activation, which instead promoted a zona fasciculata phenotype (Drelon et al., 2016). To assess whether the activation of β-catenin had any effect on the expression of zonal-specific markers, reprogrammed cells were treated with recombinant WNT4 (alongside recombinant LH). We found no significant changes in the expression of *CYP11B1*, *CYP11B2*, and *STAR* or on cortisol secretion (Figure 3D). Finally, it has been reported that the LHRH analog [D-Trp6]-LHRH (Balyura et al., 2015) and bombesin (Malendowicz et al., 1995) acutely stimulate adrenal glucocorticoid release. Both LHRH and bombesin, while not affecting *STAR* mRNA levels (Figure 3E), significantly increased cortisol production in hiSCs; however, only LHRH significantly increased the metabolic activity and lifespan of hiSCs in culture (Figure 3E).

Therefore, in our final reprogramming protocol to generate hiSCs, schematized in Figure 3F, [D-Trp6]-LHRH was included at a concentration of 1 μM starting from day two after SF1 infection.

hiSCs Are Viable When Transplanted into the Mouse Adrenal Gland or Kidney Capsule

To test cell viability *in vivo*, hiSCs from USCs were implanted in mice using three different procedures, schematized in Figure S5A: first, via ectopic implantation under the kidney capsule as free cells with fibrinogen/thrombin mixture; second, as orthotopic (intra-adrenal) implants with fibrinogen/thrombin mixture; and third, via ectopic implantation under the kidney capsule of cells embedded into alginate. No signs of necrosis or apoptosis were observed when cells were directly implanted under the kidney capsule and analyzed after one and three weeks (Figures 4A–4C). In implanted cells, nuclear SF1 staining and expression of steroidogenic enzymes, such as CYP11A1, were observed in hiSCs, but not in control cells (Figures 4E–4L). Interestingly, at three weeks post-transplantation, blood vessels could be detected in hiSC, but not control, xenografts (Figure 4C, quantification in Figure 4D), suggesting neovascularization.

To assess differentiation *in vivo*, cells were implanted orthotopically 24 hr post-infection, at a time when steroidogenic enzymes are not expressed at either the mRNA or the protein level (data not shown); 8-br-cAMP and LHRH were also omitted. Histological analyses of adrenal gland explants after one week, showed no signs of fibrosis or inflammation (Figures 4M and 4N). To localize transplanted cells in the context of host adrenocortical cells, sections were immunostained with a GFP antibody (Figures 4O–4Q); the steroidogenic potential and *in vivo* differentiation of hiSCs was further assessed by their expression of CYP17A1, an enzyme that is epigenetically silenced in adult mice (Missaghian et al., 2009); we observed CYP17A1 staining in transplanted cells, but not in mouse adrenocortical cells (Figures 4R–4T).

hiSCs embedded in alginate and cultured *in vitro* had the same gene expression profile and hormonal secretion as monolayer hiSCs (not shown); however, histological analyses of explants (after one week and three weeks) showed features of anuclear necrosis in most transplanted cells with evident signs of karyolysis (nuclear fading) and karyorrhexis (nuclear fragmentation) (Figure S5B). The same was observed in alginate slabs embedded with control cells (data not shown). These experiments demonstrate the viability of hiSCs in two out of three experimental settings and pave the way for transplantation experiments on a larger scale to assess hiSCs function in murine models of AI, in which a much greater number of reprogrammed cells are needed. However, hiSCs generated from all cell sources

Figure 4. Transplantation of hiSCs into Mouse Kidney and Adrenal Gland

(A) hiSCs implanted directly under the kidney capsule as a fibrin clot.

(B–D) H&E analyses of cells directly implanted under the kidney capsule as a fibrin clot. Blood vessels were observed to develop in explants after three weeks when hiSCs were implanted directly under the kidney capsule as a fibrin clot (C, count in D).

(E–H) Expression of SF1 in controls (E and F) and hiSCs (G and H) implanted directly under the kidney capsule.

(I–L) Expression of CYP11A1 in controls (I and J) and hiSCs (K and L) implanted directly under the kidney capsule.

(M and N) H&E analyses of a mouse adrenal gland transplanted for one week with USCs infected with SF1 24 hr earlier at lower (M) and higher (N) magnification. hiSCs can be observed at the cortex/medulla boundary (arrow in N).

(G–T) Cells transplanted into the adrenal can be visualized by the expression of GFP with immunohistochemistry (O and P), GFP indirect immunofluorescence (Q), or by their expression of CYP17A1 via immunohistochemistry (R–T). Arrows in (O)–(Q) and (S) point to transplanted cells. CYP17A1 is absent in mouse adrenal cortex (R and T). ZG, zona glomerulosa; ZF, zona fasciculata. Scale bars: 50 μm in (B), (C), (E), (G), (I), and (K); 25 μm in (F), (H), (J), (L), (N), (P), (Q), (S), (T); and 100 μm (M), (O), (R).

(U–W) Development of an inducible system aimed at generating unlimited amounts of hiSCs. (U) Schematic of the cumate vector generated to infect USCs. In repressed configuration, CymR repressor strongly binds to the cumate operator site (CuO), downstream of the CMV5 promoter. When cumate is present, CymR is released, which enables transgene expression. (V) USCs were infected with the cumate vector, selected with puromycin, and then treated with cumate or vehicle. Cells underwent similar morphologic changes to those observed in Figure 1G. Scale bars, 50 μm; inset, 20 μm. (W) qRT-PCR analyses of *STAR* expression after seven days of treatment with increasing concentration of cumate, showing induction dose dependently. Data in (W) are represented as mean ± SEM, n = 3.

See also Figure S5.

Table 1. Congenital Adrenal Hyperplasia Patients Enrolled in this Study

Patient/Donor Identification	Gene Affected	DNA	Protein
Patient #1	*CYP21A2*	c.515T > A	p.(Ile172Asn)
Patient #2	*CYP21A2*	c.955C > T	p.(Gln319Stop)
Patient #3	*STAR*	c.666delC	p.(Thr223Leufs*98)
Patient #4	*HSD3B2*	NA	NA
Patient #5	*CYP11A1*	c.940G > A	p.(Glu314Lys)
		c.990G > A	

Patient #1 presents a simple virilizing form of CAH due to one of the most common mutations in CYP21A2 (p.I172N). Patient #2 harbors a premature stop codon in *CYP21A2* at glutamine 319 (p.Q319*), which has been previously described as pathogenic. Patient #3 harbors a frameshift mutation in the STAR protein (p. Thr223fs), leading to disruption of the C-terminal START domain responsible for cholesterol binding and promotion of the translocation of cholesterol to the mitochondrial inner membrane. Patient #4 was diagnosed with HSD3B2 deficiency biochemically. Patient #5 is compound heterozygous for two putative splicing mutations in the *CYP11A1* gene, which result in exon 5 skipping and a subsequent frameshift (L.A.M., unpublished data).

were proliferation arrested (Figure 1D), and although we have been unable to find factors promoting proliferation concomitantly with the maintenance of a functional steroidogenic phenotype, a cumate-inducible system was successfully developed, allowing us to generate expandable populations of hiSCs (Figures 4U–4W).

hiSCs from CAH Patients Show Impaired Steroidogenesis, and Their Hypocortisolism Can Be Reversed through Restoration of Enzymatic Function

Currently available immortalized cell lines (mainly obtained from adrenocortical carcinomas) express certain steroidogenic enzymes at low levels, produce an incomplete steroid profile, and have genetic backgrounds specific for the donors they were derived from. Although animal models of CAH have been generated (Bornstein et al., 1999; Mullins et al., 2009), it has not been possible to design disease models involving human steroidogenic cells. For this reason, we evaluated the steroid profile of hiSCs obtained from patients with CAH (Table 1). CAH due to 21-hydroxylase deficiency (21-OH) is a common autosomal recessive disorder caused by defects in the *CYP21A2* gene. Patient #1 harbored one of the most common mutations (p.I172N), resulting in a simple virilizing form of CAH and a residual activity of the enzyme of 1%–10% (Hsu et al., 1996). LC-MS/MS steroid analyses of parallel differentiations of two independent USCs colonies obtained from patient #1 showed an accumulation of metabolites upstream of the 21-OH enzyme, including 17 hydroxyprogesterone, 17 hydroxypregnenolone, and adrenal androgens and a reduction of those downstream, including DOC and cortisol, as compared with healthy donor controls (Figure 5A). Lentiviral delivery of wild-type *CYP21A2* significantly increased cortisol levels in a dose-dependent manner concomitantly to a reduction of 17-hydroxyprogesterone, 17-hydroxypregnenolone, and testosterone (patient #1 in Figure 5B). Furthermore, rescue of cortisol hypo-secretion was successfully achieved in hiSCs established from other CAH patients with diverse genetic mutations (Table 1; Figure 5C). These results demonstrated that hiSCs derived from patients' urine are also excellent experimental models for monogenic congenital adrenal disorders and are amenable to personalized interventions for treatment.

DISCUSSION

Donor- and disease-specific steroidogenic cells as surrogates for disease modeling have been lacking up to now. More significantly, their use could be exploited for the development of cell-based treatment modalities for AI.

We have shown that the use of a single cell fate regulator (SF1/*NR5A1*), in conjunction with PKA and LHRH signaling, can stably reprogram human adult skin-, blood-, and urine-derived cells into hiSCs. Forced expression of other key TFs involved in adrenogonadal differentiation, alone or in combination, was not sufficient to induce hiSCs, nor did their expression with SF1 enhance reprogramming. However, given that there is endogenous expression of DAX1, PBX1, CITED2, and WT1 in non-reprogrammed cells, it is entirely possible that they participate in hiSC induction along with SF1, although the higher dosages delivered by our constructs did not improve reprogramming. Activation of the WNT pathway through WNT4 is associated with zona glomerulosa differentiation, which is prevented by PKA activation (Drelon et al., 2016). Treatment of hiSCs with recombinant WNT4 did not result in changes of zonal-specific markers nor cortisol secretion; it is possible that, in our experimental setting, forced-expression of SF1 bypasses key differentiation events occurring physiologically at the capsule/subcapsular region during the normal self-renewal/zonal specification of the gland.

Fibroblasts are the preferred cell substrate for reprogramming, but more recently, alternative cell types have also been used; USCs are highly expandable, have self-renewal capacity, paracrine properties, and multi-differentiation potential (Bharadwaj et al., 2013; Guan et al., 2014) and have been used as substrates for iPSC generation (Zhou et al., 2012). USC isolation is easy and efficient; we were able to establish cultures with 75% efficacy for one sample and 95% for a second sample, compared with 30% efficacy for L-EPCs (100% for fibroblasts). USCs, L-EPCs, and fibroblasts are all mesoderm derived, however, only USCs are thought to originate from the intermediate mesoderm, a precursor of the adrenocortical and nephritic primordium, which make them an ideal substrate to generate mesodermal tissues, such as adrenogonadal-like cells (Mazilu and McCabe, 2011b). Given the importance of SF1 dosage during adrenal versus gonadal specification (Bland et al., 2004; Val et al., 2007), further development of inducible and tunable systems to modulate the expression of SF1 would likely facilitate a more specific adrenocortical or gonadal induction during reprogramming. Gene expression analyses of adrenocortical and gonadal markers already suggested that our protocol might favor a more adrenocortical phenotype (Figure S3D).

The functionality of hiSCs makes them an unmatched tool to obtain surrogate adrenocortical cells for *in vitro* disease modeling. With this in mind, we have derived hiSCs from patients

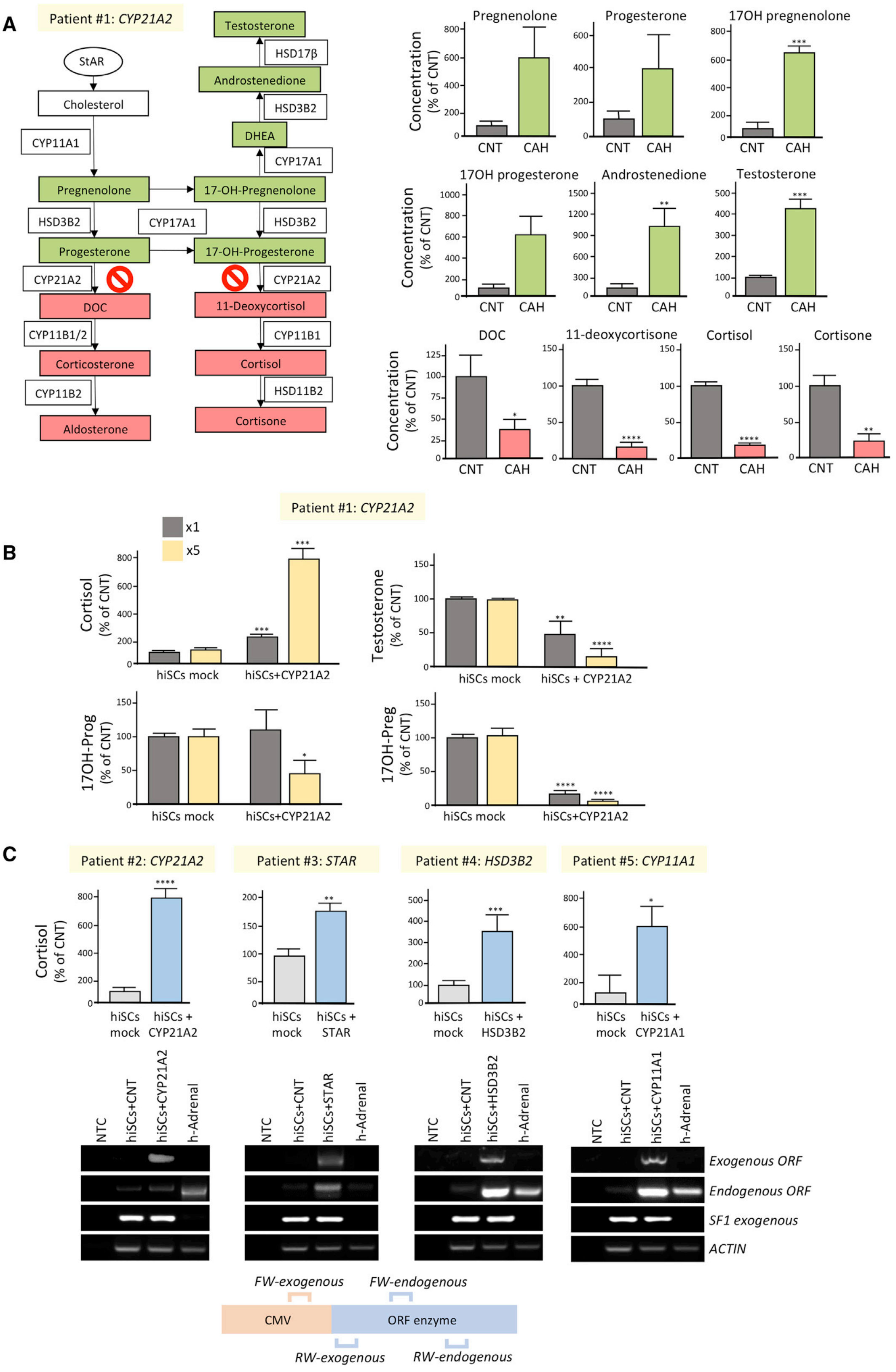

(legend on next page)

with CAH, showing an altered steroid profile. Importantly, the decrease in cortisol production in hiSCs derived from CAH patients was rescued on expression of the exogenous native forms, irrespective of the defective steroidogenic enzyme.

USCs-hiSCs were shown to be viable *in vivo* when transplanted into mouse adrenal gland tissue and successfully differentiate toward an adrenal-like lineage within the adrenal tissue itself, as cells were implanted 24 hr post-infection when steroidogenic enzymes are not expressed. Whether the surrounding adrenal tissue favors differentiation remains to be explored, as well as the long-term viability/functionality and fate of transplanted cells. hiSCs implanted under the mouse kidney capsule maintained the expression of steroidogenic enzymes and showed well-vascularized tissue at the site of transplantation after three weeks, suggesting viability of those cells *in vivo*. Remarkably, vascularization was not observed in control implants; this can be explained either by the intrinsic ability of SF1 to regulate adrenocortical vascular remodeling through the expression of angiopoietin-2 (Ferraz-de-Souza et al., 2011) or by the very significant upregulation of the potent pro-angiogenic tetraspartin CD9 in hiSCs detected in a proteomic array in all cell sources (Figure S6). CD9, also expressed in the normal adrenal, but with unknown function (Nakamura et al., 2001), exerts its angiogenic action via exosomes, where it is abundantly expressed (Andreu and Yáñez-Mó, 2014).

Sodium alginate has been used as a 3D scaffold for cell immobilization/delivery in tissue engineering (Bidarra et al., 2014) and might confer some immunoprotection (Llacua et al., 2016). However, hiSC viability was compromised on implantation; control cells were also negatively affected, suggesting cell-specific toxicity of alginate after grafting in our system (both hiSCs and control cells embedded in alginate were viable and functional *in vitro*).

Overall, these pilot transplantation experiments showed that viability of hiSCs is affected by the transplantation protocol used. Longer-term experiments with a yet-to-be determined amount of cells would allow for careful assessment of: (1) the number of cells needed to be able to detect cortisol (undetectable in our pilot experiments); (2) the time needed to become fully functional; (3) their viability long term; and (4) their rescuing potential in animal models of AI.

Novel approaches to treating AI are needed for a condition in which mortality rates continue to be 2-fold higher than the background population despite steroid replacement (Bergthorsdottir et al., 2006). Although newer steroid formulations (e.g., Plenadren and Chronocort) are being developed to mimic circadian rhythms more precisely, replacement remains inherently limited in its responsiveness to physiological need. This study paves the way for the development of alternative approaches to treat AI. For example, hiSCs could be implanted intra-adrenally or encapsulated using immunoisolating chambers, possibly after gene-editing in case of mono/oligogenic disorders, as previously used in beta-cell transplantation (Desai and Shea, 2017). To this end, USCs have recently been successfully gene edited in a model of human muscle disease (Kim et al., 2016). This will allow a curative cell replacement therapy for patients with AI.

EXPERIMENTAL PROCEDURES

Further details and an outline of resources used in this work can be found in Supplemental Experimental Procedures.

Materials

All antibodies, plasmids, and primers used are listed in Supplemental Experimental Procedures.

Cell Culture

Primary cultures of USCs, fibroblasts, and L-EPCs were isolated as previously described (Martin-Ramirez et al., 2012; Poliandri et al., 2017; Zhou et al., 2012). BMECs were a kind gift of Dr. Egle Solito (Schweitzer et al., 1997; Solito et al., 2000), and HEK293T lines were obtained from ATCC. SF1-inducible cell lines were generated using a SparQ cumate switch pCDH-CuO-MCS-IRES-GFP-EF1-CymR-T2A-Puro All-in-one inducible lentivector (Cambridge Bioscience, Ltd.) expressing SF1. The detailed protocols for the isolation of all cell types are provided in Supplemental Experimental Procedures.

Lentiviral Production and Cell Reprogramming

Lentiviral particles were obtained as previously described (Cribbs et al., 2013) with minor modifications. 60,000 cells per well of a six-well plate were infected with lentiviral particles at a MOI of 200 with 8 μg/mL of polybrene (Millipore, TR-1003-G). The medium was replaced after 12 hr, and treatments with different molecules were started after two days. Cells were cultured for an additional 5–10 days before analysis. Drug concentrations are detailed in Supplemental Experimental Procedures.

Gene Expression Analysis

See Supplemental Experimental Procedures for details.

Immunoassays

Immunoblotting, immunocytochemistry, and immunohistochemistry were performed as described previously (Guasti et al., 2011; Rodríguez-Asiain et al., 2011). See Supplemental Experimental Procedures for details.

Hormone Quantification

Hormone quantification was performed using an ELISA Kit (Abcam, ab154996) according to the manufacturer's instructions and LC-MS/MS (Taylor et al., 2017). See Supplemental Experimental Procedures for details.

Mitochondrial Morphology Studies

Electron microscopy was performed by the Nanovision Centre, Queen Mary University of London, using standardized procedures.

Figure 5. Characterization of Urine-Derived hiSCs Established from Patients with CAH

(A) Comparison of steroidogenic profile of hiSCs established from patient #1 with *CYP21A2* mutation (CAH) versus healthy donors (CNT). The diagram on the left shows the steroidogenic pathway with increased metabolites in patient #1 highlighted in green and decreased ones in red.

(B) Comparison of cortisol, 17-hydroxyprogesterone, 17-hydroxypregnenolone, and testosterone levels of hiSCs derived from patient #1 with or without restoration of the wild-type form 21-OH. Cells were infected with two increasing amounts of lentiviral particles (×1 and ×5).

(C) Comparison of cortisol levels of hiSCs derived from patients (#2–#5) with several forms of CAH with or without restoration of the wild-type form of the corresponding steroidogenic enzymes. RT-PCR analyses using primers encompassing coding- and vector-specific regions confirmed the expression of the exogenous enzymes (lower panels). See also Table 1. Data are represented as mean ± SEM, n ≥ 3.

Animal Experiments

All animal experiments were performed in strict accordance with protocols approved by the ethical board of Landesdirektion Sachsen, Germany (protocol no. DD24-5131/354/28). Female 8-week-old C57BL/6 mice were obtained from Charles River Laboratory. Two million cells were transplanted under the kidney capsule and 5×10^5 cells concentrated in a total volume of 10 μL were pipetted directly into the adrenal gland.

See Supplemental Experimental Procedures for details.

Collection of Urine Samples from Donors

This study has been performed under the ethical approval of the National Health Service, Research Ethic Committee (NHS REC; reference: 13/LO/0224). All patients involved were previously informed, and consent forms were obtained before analysis of samples.

Protein Array of Human Reprogrammed Steroidogenic Cells

Protein array was performed by Sciomics, GmbH (Germany) using control and eight-day reprogrammed hiSCs. See Supplemental Experimental Procedures for details.

Data Analysis

Figures and tables were generated using Microsoft Excel, ImageJ, and Adobe Photoshop.

Statistical Analysis

Statistical analysis was performed using GraphPad Prism 7, and statistical significance was determined using one-way ANOVA followed by Dunnet's multiple comparison test correction. A Student's t test was performed when only two means were compared. Significance: not significant (ns) $p > 0.05$; $*p < 0.05$; $**p < 0.01$; $***p < 0.001$; $****p < 0.0001$.

DATA AND SOFTWARE AVAILABILITY

The accession number for the protein array data reported in this article is Mendeley Data: https://doi.org/10.17632/x9rn9kcr3b.2.

SUPPLEMENTAL INFORMATION

Supplemental Information includes Supplemental Experimental Procedures, six figures, and one movie and can be found with this article online at https://doi.org/10.1016/j.celrep.2018.01.003.

ACKNOWLEDGMENTS

We are extremely grateful to Dr. Alex da Costa, Dr. Sam O'Toole, Ms. Chedna Varsani for sample collection, Dr. David Poitz for his support during *in vivo* experiments, Dr. Ariel Poliandri and Dr. Duncan Miller for providing fibroblasts, and Lisa Romano for microscopy support. This work was supported by the Biotechnology and Biological Sciences Research Council (BBSRC New Investigator BB/L002671/1, to L. Guasti), the International Fund for research on Congenital Adrenal Hyperplasia (IFCAH, to L. Guasti), the Rosetrees Trust (M335, to L. Guasti), Barts and the London Charity (BTLC 417-2235 to L. Guasti), the Society for Endocrinology (to G.R.B.), the European Union Marie Curie COFUND (to L. Guasti and G.R.-B.), and DAAD (to G.R.-B.). I.H. was supported by a Medical Research Council- Queen Mary University Of London (MRC-QMUL) Ph.D. studentship. M.B., U.S., C.Z.G., and S.R.B. were supported by the Kings College London (KCL) and Technische Universität Dresden (TUD) (SFB/transregio 205 and IRTG 2251, SFB/transregio 127).

AUTHOR CONTRIBUTIONS

L. Guasti supervised the study. L. Guasti and G.R.-B. conceived and performed the experiments, wrote the manuscript with input from all authors, and secured funding. M.B. and U.S. performed the *in vivo* transplantation experiments. I.H. performed immunohistochemistry. S.J.A. provided technical support with gene expression analyses and ELISA. D.R.T., L. Ghataore, and N.F.T. analyzed samples using mass spectrometry. H.L.S., M.R.D., E.F.J., W.M.D., U.S., L.A.M., and G.S.C. provided patient referrals and data. P.J.K., L.A.M., C.G.Z., and S.R.B. provided expertise and feedback throughout the project.

DECLARATION OF INTERESTS

The authors declare no competing interests.

Received: September 7, 2017
Revised: November 28, 2017
Accepted: December 29, 2017
Published: January 30, 2018

REFERENCES

Achermann, J.C., Meeks, J.J., and Jameson, J.L. (2001). Phenotypic spectrum of mutations in DAX-1 and SF-1. Mol. Cell. Endocrinol. *185*, 17–25.

Andreu, Z., and Yáñez-Mó, M. (2014). Tetraspanins in extracellular vesicle formation and function. Front. Immunol. *5*, 442.

Balyura, M., Gelfgat, E., Ehrhart-Bornstein, M., Ludwig, B., Gendler, Z., Barkai, U., Zimerman, B., Rotem, A., Block, N.L., Schally, A.V., and Bornstein, S.R. (2015). Transplantation of bovine adrenocortical cells encapsulated in alginate. Proc. Natl. Acad. Sci. USA *112*, 2527–2532.

Bergthorsdottir, R., Leonsson-Zachrisson, M., Odén, A., and Johannsson, G. (2006). Premature mortality in patients with Addison's disease: a population-based study. J. Clin. Endocrinol. Metab. *91*, 4849–4853.

Bharadwaj, S., Liu, G., Shi, Y., Wu, R., Yang, B., He, T., Fan, Y., Lu, X., Zhou, X., Liu, H., et al. (2013). Multipotential differentiation of human urine-derived stem cells: potential for therapeutic applications in urology. Stem Cells *31*, 1840–1856.

Bidarra, S.J., Barrias, C.C., and Granja, P.L. (2014). Injectable alginate hydrogels for cell delivery in tissue engineering. Acta Biomater. *10*, 1646–1662.

Bland, M.L., Fowkes, R.C., and Ingraham, H.A. (2004). Differential requirement for steroidogenic factor-1 gene dosage in adrenal development versus endocrine function. Mol. Endocrinol. *18*, 941–952.

Bornstein, S.R., Tajima, T., Eisenhofer, G., Haidan, A., and Aguilera, G. (1999). Adrenomedullary function is severely impaired in 21-hydroxylase-deficient mice. FASEB J. *13*, 1185–1194.

Bornstein, S.R., Allolio, B., Arlt, W., Barthel, A., Don-Wauchope, A., Hammer, G.D., Husebye, E.S., Merke, D.P., Murad, M.H., Stratakis, C.A., and Torpy, D.J. (2016). Diagnosis and treatment of primary adrenal insufficiency: an endocrine society clinical practice guideline. J. Clin. Endocrinol. Metab. *101*, 364–389.

Crawford, P.A., Sadovsky, Y., and Milbrandt, J. (1997). Nuclear receptor steroidogenic factor 1 directs embryonic stem cells toward the steroidogenic lineage. Mol. Cell. Biol. *17*, 3997–4006.

Cribbs, A.P., Kennedy, A., Gregory, B., and Brennan, F.M. (2013). Simplified production and concentration of lentiviral vectors to achieve high transduction in primary human T cells. BMC Biotechnol. *13*, 98.

Desai, T., and Shea, L.D. (2017). Advances in islet encapsulation technologies. Nat. Rev. Drug Discov. *16*, 338–350.

Drelon, C., Berthon, A., Sahut-Barnola, I., Mathieu, M., Dumontet, T., Rodriguez, S., Batisse-Lignier, M., Tabbal, H., Tauveron, I., Lefrançois-Martinez, A.-M., et al. (2016). PKA inhibits WNT signalling in adrenal cortex zonation and prevents malignant tumour development. Nat. Commun. *7*, 12751.

Ferraz-de-Souza, B., Lin, L., Shah, S., Jina, N., Hubank, M., Dattani, M.T., and Achermann, J.C. (2011). ChIP-on-chip analysis reveals angiopoietin 2 (Ang2, ANGPT2) as a novel target of steroidogenic factor-1 (SF-1, NR5A1) in the human adrenal gland. FASEB J. *25*, 1166–1175.

Gondo, S., Yanase, T., Okabe, T., Tanaka, T., Morinaga, H., Nomura, M., Goto, K., and Nawata, H. (2004). SF-1/Ad4BP transforms primary long-term cultured bone marrow cells into ACTH-responsive steroidogenic cells. Genes Cells *9*, 1239–1247.

Gondo, S., Okabe, T., Tanaka, T., Morinaga, H., Nomura, M., Takayanagi, R., Nawata, H., and Yanase, T. (2008). Adipose tissue-derived and bone marrow-derived mesenchymal cells develop into different lineage of steroidogenic cells by forced expression of steroidogenic factor 1. Endocrinology *149*, 4717–4725.

Guan, J.J., Niu, X., Gong, F.X., Hu, B., Guo, S.C., Lou, Y.L., Zhang, C.Q., Deng, Z.F., and Wang, Y. (2014). Biological characteristics of human-urine-derived stem cells: potential for cell-based therapy in neurology. Tissue Eng. Part A *20*, 1794–1806.

Guasti, L., Paul, A., Laufer, E., and King, P. (2011). Localization of Sonic hedgehog secreting and receiving cells in the developing and adult rat adrenal cortex. Mol. Cell. Endocrinol. *336*, 117–122.

Hsu, L.C., Hsu, N.C., Guzova, J.A., Guzov, V.M., Chang, S.F., and Chung, B.C. (1996). The common I172N mutation causes conformational change of cytochrome P450c21 revealed by systematic mutation, kinetic, and structural studies. J. Biol. Chem. *271*, 3306–3310.

Jadhav, U., and Jameson, J.L. (2011). Steroidogenic factor-1 (SF-1)-driven differentiation of murine embryonic stem (ES) cells into a gonadal lineage. Endocrinology *152*, 2870–2882.

Kim, E.Y., Page, P., Dellefave-Castillo, L.M., McNally, E.M., and Wyatt, E.J. (2016). Direct reprogramming of urine-derived cells with inducible MyoD for modeling human muscle disease. Skelet. Muscle *6*, 32.

Lerario, A.M., Finco, I., LaPensee, C., and Hammer, G.D. (2017). Molecular mechanisms of stem/progenitor cell maintenance in the adrenal cortex. Front. Endocrinol. (Lausanne) *8*, 52.

Lewis, D.A., Melchitzky, D.S., and Haycock, J.W. (1993). Four isoforms of tyrosine hydroxylase are expressed in human brain. Neuroscience *54*, 477–492.

Llacua, A., de Haan, B.J., Smink, S.A., and de Vos, P. (2016). Extracellular matrix components supporting human islet function in alginate-based immunoprotective microcapsules for treatment of diabetes. J. Biomed. Mater. Res. A *104*, 1788–1796.

Malendowicz, L.K., Nussdorfer, G.G., Miskowiak, B., and Majchrzak, M. (1995). Effects of bombesin on the morphology and function of the rat adrenal cortex: comparison of the acute and chronic responses. Histol. Histopathol. *10*, 11–15.

Martin-Ramirez, J., Hofman, M., van den Biggelaar, M., Hebbel, R.P., and Voorberg, J. (2012). Establishment of outgrowth endothelial cells from peripheral blood. Nat. Protoc. *7*, 1709–1715.

Mazilu, J.K., and McCabe, E.R.B. (2011a). Moving toward personalized cell-based interventions for adrenal cortical disorders: part 2–Human diseases and tissue engineering. Mol. Genet. Metab. *104*, 80–88.

Mazilu, J.K., and McCabe, E.R.B. (2011b). Moving toward personalized cell-based interventions for adrenal cortical disorders: part 1–Adrenal development and function, and roles of transcription factors and signaling proteins. Mol. Genet. Metab. *104*, 72–79.

Merke, D.P., and Bornstein, S.R. (2005). Congenital adrenal hyperplasia. Lancet *365*, 2125–2136.

Missaghian, E., Kempná, P., Dick, B., Hirsch, A., Alikhani-Koupaei, R., Jégou, B., Mullis, P.E., Frey, B.M., and Flück, C.E. (2009). Role of DNA methylation in the tissue-specific expression of the CYP17A1 gene for steroidogenesis in rodents. J. Endocrinol. *202*, 99–109.

Mullins, L.J., Peter, A., Wrobel, N., McNeilly, J.R., McNeilly, A.S., Al-Dujaili, E.A.S., Brownstein, D.G., Mullins, J.J., and Kenyon, C.J. (2009). Cyp11b1 null mouse, a model of congenital adrenal hyperplasia. J. Biol. Chem. *284*, 3925–3934.

Nakamura, Y., Handa, K., Iwamoto, R., Tsukamoto, T., Takahasi, M., and Mekada, E. (2001). Immunohistochemical distribution of CD9, heparin binding epidermal growth factor-like growth factor, and integrin α3β1 in normal human tissues. J. Histochem. Cytochem. *49*, 439–444.

Poliandri, A., Miller, D., Howard, S., Nobles, M., Ruiz-Babot, G., Harmer, S., Tinker, A., McKay, T., Guasti, L., and Dunkel, L. (2017). Generation of kisspeptin-responsive GnRH neurons from human pluripotent stem cells. Mol. Cell. Endocrinol. *447*, 12–22.

Rodríguez-Asiain, A., Ruiz-Babot, G., Romero, W., Cubí, R., Erazo, T., Biondi, R.M., Bayascas, J.R., Aguilera, J., Gómez, N., Gil, C., et al. (2011). Brain specific kinase-1 BRSK1/SAD-B associates with lipid rafts: modulation of kinase activity by lipid environment. Biochim. Biophys. Acta *1811*, 1124–1135.

Ruggiero, C., and Lalli, E. (2016). Impact of ACTH Signaling on Transcriptional Regulation of Steroidogenic Genes. Front. Endocrinol. (Lausanne) *7*, 24.

Ruiz-Babot, G., Hadjidemetriou, I., King, P.J., and Guasti, L. (2015). New directions for the treatment of adrenal insufficiency. Front. Endocrinol. (Lausanne) *6*, 70.

Schimmer, B.P., and White, P.C. (2010). Minireview: steroidogenic factor 1: its roles in differentiation, development, and disease. Mol. Endocrinol. *24*, 1322–1337.

Schweitzer, K.M., Vicart, P., Delouis, C., Paulin, D., Dräger, A.M., Langenhuijsen, M.M., and Weksler, B.B. (1997). Characterization of a newly established human bone marrow endothelial cell line: distinct adhesive properties for hematopoietic progenitors compared with human umbilical vein endothelial cells. Lab. Invest. *76*, 25–36.

Solito, E., Romero, I.A., Marullo, S., Russo-Marie, F., and Weksler, B.B. (2000). Annexin 1 binds to U937 monocytic cells and inhibits their adhesion to microvascular endothelium: involvement of the α 4 β 1 integrin. J. Immunol. *165*, 1573–1581.

Sonoyama, T., Sone, M., Honda, K., Taura, D., Kojima, K., Inuzuka, M., Kanamoto, N., Tamura, N., and Nakao, K. (2012). Differentiation of human embryonic stem cells and human induced pluripotent stem cells into steroid-producing cells. Endocrinology *153*, 4336–4345.

Tanaka, T., Gondo, S., Okabe, T., Ohe, K., Shirohzu, H., Morinaga, H., Nomura, M., Tani, K., Takayanagi, R., Nawata, H., and Yanase, T. (2007). Steroidogenic factor 1/adrenal 4 binding protein transforms human bone marrow mesenchymal cells into steroidogenic cells. J. Mol. Endocrinol. *39*, 343–350.

Taylor, D.R., Ghataore, L., Couchman, L., Vincent, R.P., Whitelaw, B., Lewis, D., Diaz-Cano, S., Galata, G., Schulte, K.-M., Aylwin, S., and Taylor, N.F. (2017). A 13-steroid serum panel based on LC-MS/MS: use in detection of adrenocortical carcinoma. Clin. Chem. *63*, 1836–1846.

Val, P., Martinez-Barbera, J.-P., and Swain, A. (2007). Adrenal development is initiated by Cited2 and Wt1 through modulation of Sf-1 dosage. Development *134*, 2349–2358.

Vogeser, M., Zachoval, R., and Jacob, K. (2001). Serum cortisol/cortisone ratio after Synacthen stimulation. Clin. Biochem. *34*, 421–425.

Wei, X., Peng, G., Zheng, S., and Wu, X. (2012). Differentiation of umbilical cord mesenchymal stem cells into steroidogenic cells in comparison to bone marrow mesenchymal stem cells. Cell Prolif. *45*, 101–110.

Xing, Y., Edwards, M.A., Ahlem, C., Kennedy, M., Cohen, A., Gomez-Sanchez, C.E., and Rainey, W.E. (2011). The effects of ACTH on steroid metabolomic profiles in human adrenal cells. J. Endocrinol. *209*, 327–335.

Yates, R., Katugampola, H., Cavlan, D., Cogger, K., Meimaridou, E., Hughes, C., Metherell, L., Guasti, L., and King, P. (2013). Adrenocortical Development, Maintenance, and Disease (Elsevier Inc.).

Yazawa, T., Mizutani, T., Yamada, K., Kawata, H., Sekiguchi, T., Yoshino, M., Kajitani, T., Shou, Z., Umezawa, A., and Miyamoto, K. (2006). Differentiation of adult stem cells derived from bone marrow stroma into Leydig or adrenocortical cells. Endocrinology *147*, 4104–4111.

Yazawa, T., Inanoka, Y., Mizutani, T., Kuribayashi, M., Umezawa, A., and Miyamoto, K. (2009). Liver receptor homolog-1 regulates the transcription of steroidogenic enzymes and induces the differentiation of mesenchymal stem cells into steroidogenic cells. Endocrinology *150*, 3885–3893.

Yazawa, T., Inaoka, Y., Okada, R., Mizutani, T., Yamazaki, Y., Usami, Y., Kuribayashi, M., Orisaka, M., Umezawa, A., and Miyamoto, K. (2010). PPAR-gamma coactivator-1alpha regulates progesterone production in ovarian granulosa cells with SF-1 and LRH-1. Mol. Endocrinol. *24*, 485–496.

Yazawa, T., Kawabe, S., Inaoka, Y., Okada, R., Mizutani, T., Imamichi, Y., Ju, Y., Yamazaki, Y., Usami, Y., Kuribayashi, M., et al. (2011). Differentiation of mesenchymal stem cells and embryonic stem cells into steroidogenic cells using steroidogenic factor-1 and liver receptor homolog-1. Mol. Cell. Endocrinol. *336*, 127–132.

Yoder, M.C., Blanco, R., Gerhardt, H., Lampugnani, M.G., Thurston, G., Daly, C., Yoder, M.C., and Li, Y. (2012). Human endothelial progenitor cells. 1–14.

Zhang, X., Huang, C.T., Chen, J., Pankratz, M.T., Xi, J., Li, J., Yang, Y., Lavaute, T.M., Li, X.-J., Ayala, M., et al. (2010). Pax6 is a human neuroectoderm cell fate determinant. Cell Stem Cell *7*, 90–100.

Zhou, T., Benda, C., Dunzinger, S., Huang, Y., Ho, J.C., Yang, J., Wang, Y., Zhang, Y., Zhuang, Q., Li, Y., et al. (2012). Generation of human induced pluripotent stem cells from urine samples. Nat. Protoc. *7*, 2080–2089.

Cell Reports, Volume 22

Supplemental Information

Modeling Congenital Adrenal Hyperplasia and Testing Interventions for Adrenal Insufficiency Using Donor-Specific Reprogrammed Cells

Gerard Ruiz-Babot, Mariya Balyura, Irene Hadjidemetriou, Sharon J. Ajodha, David R. Taylor, Lea Ghataore, Norman F. Taylor, Undine Schubert, Christian G. Ziegler, Helen L. Storr, Maralyn R. Druce, Evelien F. Gevers, William M. Drake, Umasuthan Srirangalingam, Gerard S. Conway, Peter J. King, Louise A. Metherell, Stefan R. Bornstein, and Leonardo Guasti

Figure S1 (related to Figure 1)

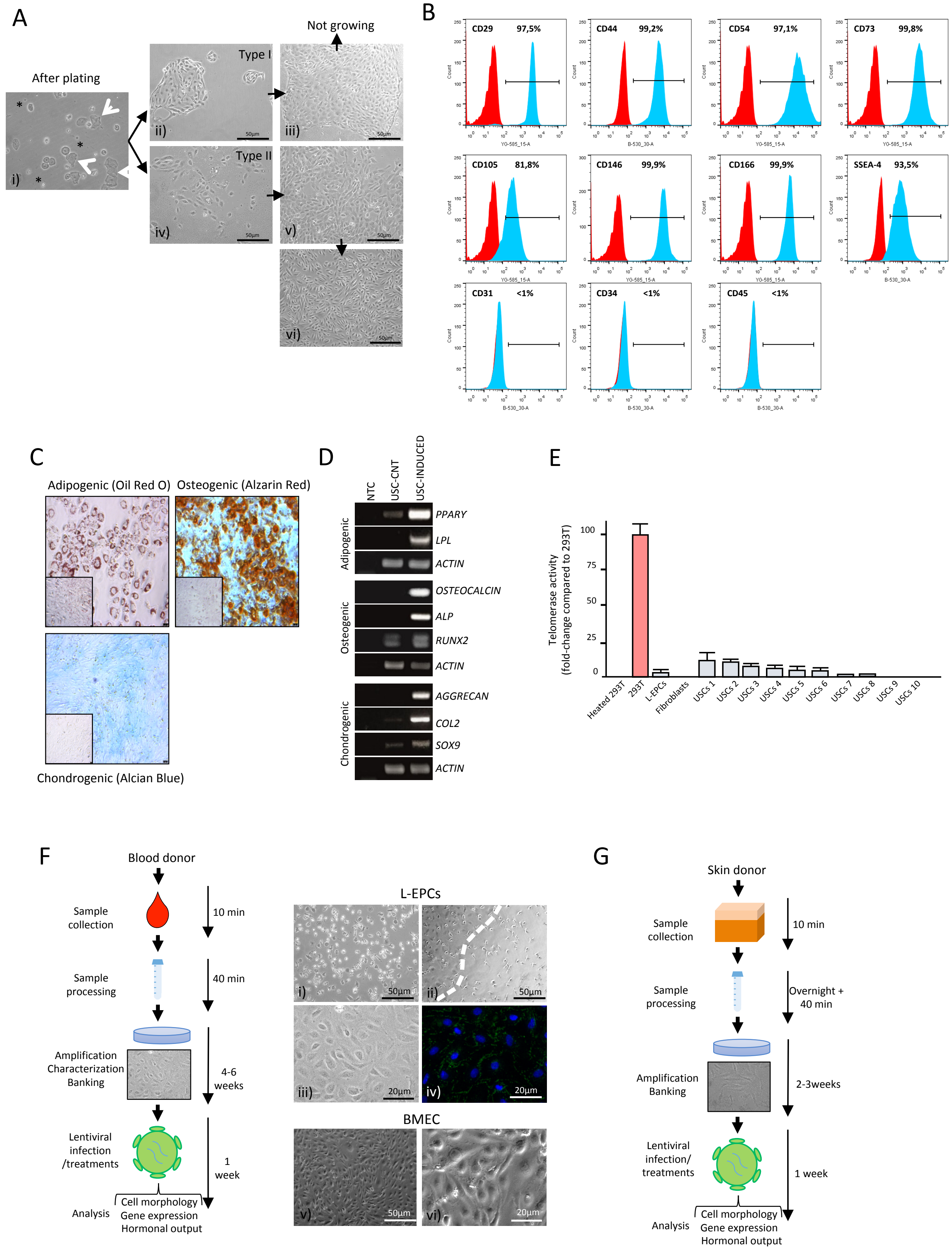

Figure Suppl. 1. Establishment and characterization of human primary cells from donors.
A) Isolation of urine-derived stem cells (USCs). Two main urine-derived cell populations generated large colonies within a few days in culture: colonies with defined edges, which were proliferation-arrested past passage 1 (named type-I colonies), and those characterized by cells with a rice-grain morphology, which were viable for several passages and were bankable (named type-II colonies, representing USCs[1]). **i)** Cells after plating. Arrows indicate squamous cells and asterisks blood cells. **ii-iii)** Morphology of a type-I colony. **iv-v)** Morphology of a type-II colony (USCs). **vi)** Type-II colony at confluency showing the typical rice-grain morphology. Scale bars = 50 µm. **B)** The expression of cell surface marker in USCs at passage 2 was assessed by flow cytometry. USCs expressed mesenchymal (CD29, CD44, CD54, CD73, CD105, CD146, CD166), pluripotent (SSEA-4), but not hematopoietic (CD31, CD34, CD45) stem cell markers. The peaks in red represent the signal relative to the isotype controls and those in light blue the CD specific signal. **C)** USCs had the potential to differentiate to adipogenic, chondrogenic and osteogenic lineages using specific induction media. Confluent USCs were treated with adipogenic, chondrogenic and osteogenic media for 21 days and then stained with Oil Red O, Alcian Blue and Alizarin Red, respectively. Parallel cultures from the same non-induced colony are shown in the insets. **D)** RT-PCR expression analyses of osteogenic, adipogenic, and chondrogenic lineage markers. *PPARg*, peroxisome proliferator-activated receptor gamma; *LPL*, lipoprotein lipase; *ALP*, alkaline phosphatase; *RUNX2*, runt-related transcription factor 2; *COL2*, collagen 2; *SOX9*, Sry-related HMG box-9. **E)** Measurement of telomerase activity. Eight out of ten individual USC clones expressed detectable levels of telomerase. **F)** Schematic illustrating our strategy for blood collection, processing and reprogramming. L-EPC colonies displayed the characteristic cobblestone morphology[2] and appeared after 3-5 weeks of culturing mononuclear cells. **i)** Mononuclear cells after plating. **ii)** Edge of a L-EPCs with single mononuclear cells around. **iii)** L-EPCs at confluency. **iv)** L-EPCs grew as highly proliferating and tightly attached cells expressing Zona Occludens-1. **v** and **vii)** Bone marrow-derived endothelial cells, BMEC[3,4]. Scale bars i, ii and v = 50 µm; iii, iv and vi = 20 µm. **G)** Schematic illustrating our strategy for skin collection, processing and reprogramming of fibroblasts. Data in **E** are represented as mean ± SEM.

Figure S2 (related to Figure 1)

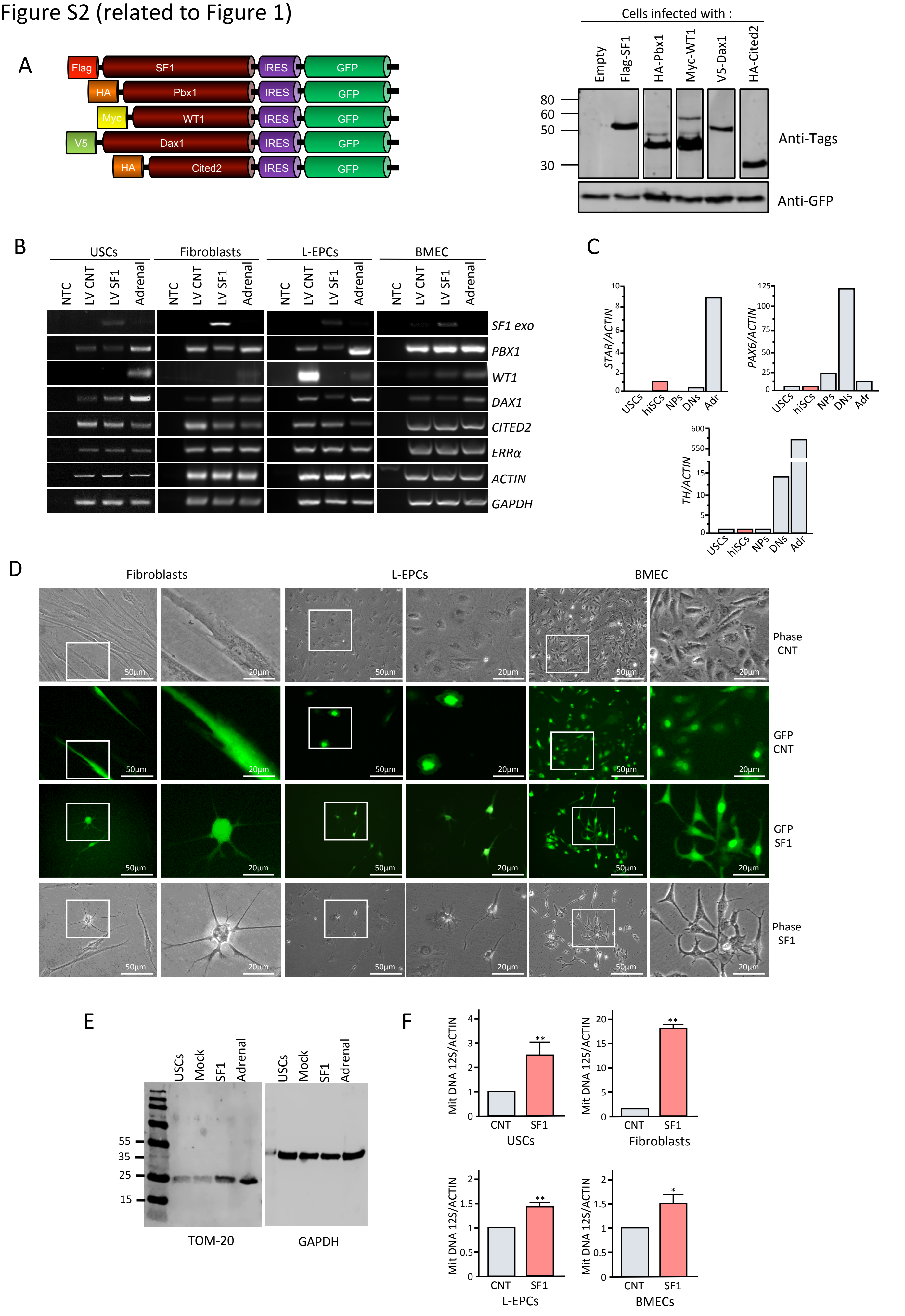

Figure Suppl. 2. Reprogramming, morphologic changes and gene expression in human cells.
A) Schematic representation of the lentiviral vectors used to force the expression of tagged transcription factors Flag-SF1, HA-PBX1, Myc-WT1, V5-DAX1 and HA-CITED2. Their expression in infected cells was assessed by western blot using anti-tag antibodies, and by using anti-GFP antibody as a loading control (right panels). **B)** Expression of exogenous *SF1* and endogenous *PBX1*, *WT1*, *DAX1*, *CITED2*, *ERRa*, *ACTIN* and *GAPDH* in reprogrammed (SF1), mock-reprogrammed (CNT) USCs, fibroblasts, L-EPCs, BMEC and human adrenal. NTC, no template control. **C)** RT-qPCR analyses of *STAR*, *PAX6* and *TH* expression in USCs, USCs-hiSCs (8 days differentiation), neuronal precursors (NPs), neurons differentiated *in vitro* from NPs for 12 days (DNs) and human adrenal. **D)** Morphological changes upon SF1 overexpression and br-cAMP treatment in fibroblasts, L-EPCs and BMEC 8 days post-infection. Scale bars = 50 µm, for inset = 20 µm. **E**) Expression of TOM20 protein in control and reprogrammed cells. **F**) Expression of mitochondrial DNA 12S in control and reprogrammed cells. Data in F are represented as mean ± SEM.

Figure S3 (related to Figure 2)

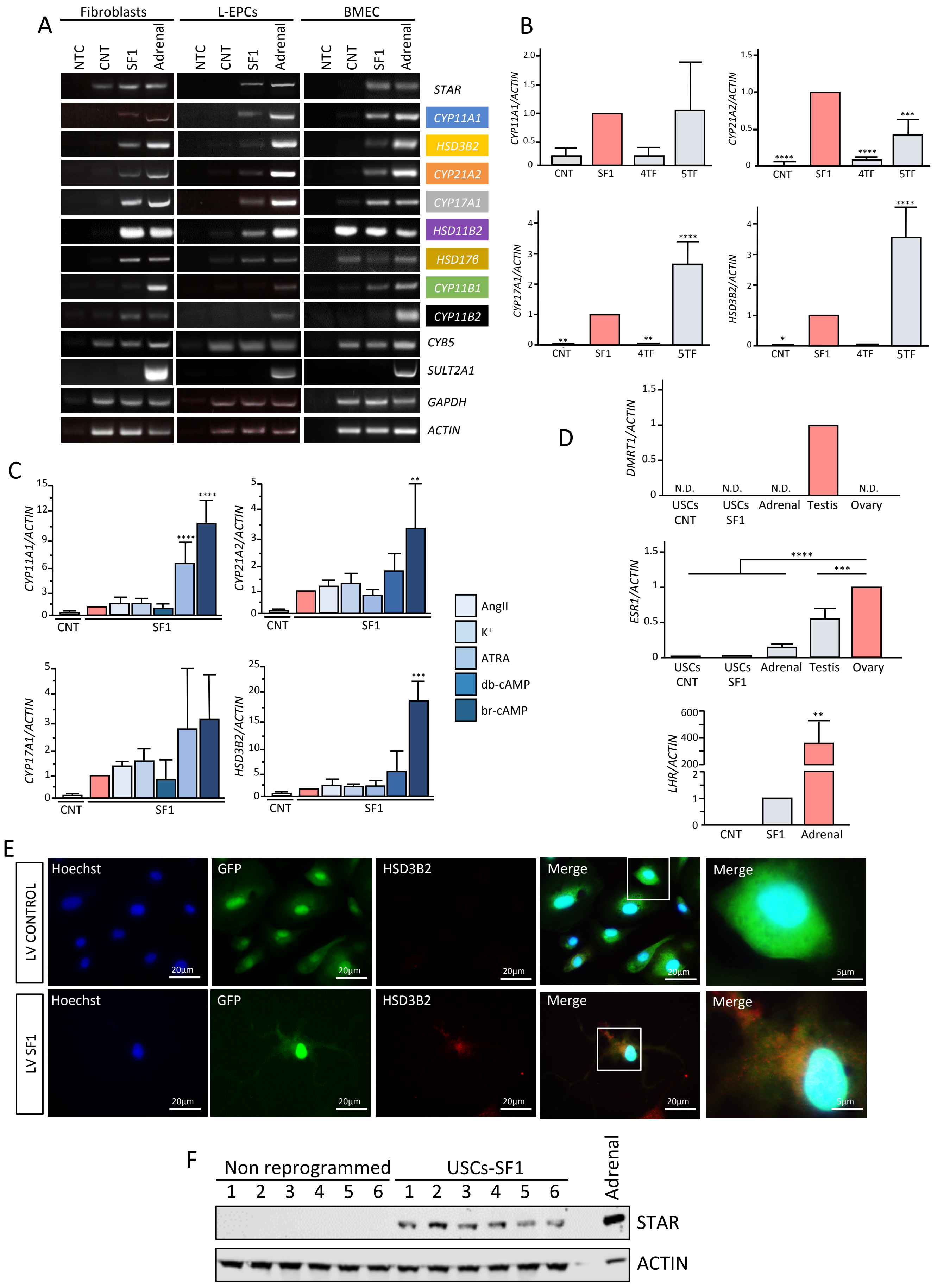

Figure Suppl. 3. Reprogramming and gene expression in human cells.
A) RT-PCR expression analyses of *STAR*, steroidogenic enzymes, *SULT2A1*, *GAPDH* and *ACTIN* in cells infected with SF1 or control (CNT) lentiviruses after 8 days. Human adrenal cDNA was used as a positive control. All cells were treated with br-cAMP. NTC, no template control, Adr, human adrenal. **B)** RT-qPCR analyses of the expression of *CYP11A1*, *CYP21A2*, *CYP17A1* and *HSD3B2* in cells infected with control lentivirus, lentivirus encoding SF1, 4TF (PBX1, WT1, DAX1 and CITED2) and 5 TF (4TF+SF1). **C)** RT-qPCR analyses of the expression of *CYP11A1*, *CYP21A2*, *CYP17A1* and *HSD3B2* in cells infected with control lentivirus and lentivirus encoding SF1, followed by treatments with AngII, KCl, ATRA, db-cAMP and br-cAMP for 8 days. **D)** RT-qPCR analyses of the expression of *DMRT1* (top panel), *ESR1* (middle panel) in cells infected with control lentivirus, lentivirus encoding SF1, as well as in human adrenal, testes and ovary. The bottom panel shows RT-qPCR analyses of the expression of *LHR* in cells infected with control lentivirus, lentivirus encoding SF1, and in human adrenal. **E)** HSD3B2 immunocytochemistry in control USCs and USCs infected with SF1 after 8 days *in vitro*. Scale bars = 20 µm, for inset = 5 µm **F)** Western blot analyses of STAR protein levels in 6 independent donors 8 days after reprogramming. Data in **B, C** and **D** are represented as mean ± SEM.

Figure S4 (related to Figure 3)

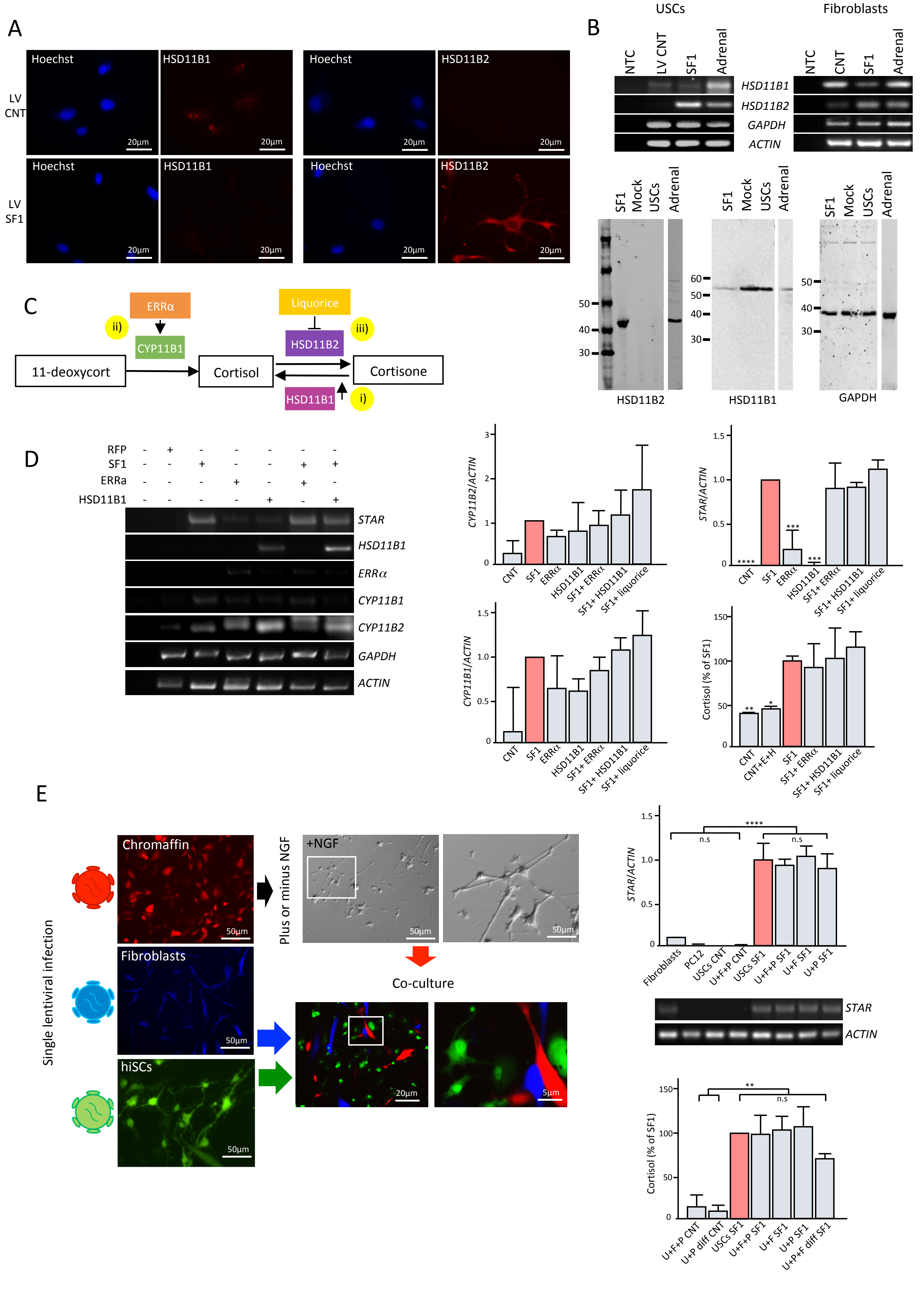

Figure Suppl. 4. Modulation of steroidogenic pathways involved in cortisol production.
The low cortisol/cortisone ratio observed in hiSCs could be due to an enhanced expression of the enzyme hydroxysteroid 11-β dehydrogenase 2 (HSD11B2), responsible for the conversion of cortisol to cortisone, or a downregulation of hydroxysteroid 11-β dehydrogenase 1 (HSD11B1), which catalyzes the reverse reaction in hiSCs compared to controls. **A)** Immunocytochemistry of HSD11B1 and HSD11B2 in control and SF1-infected cells 8 days post-infection. Scale bars = 20 µm. **B)** HSD11B1 and HSD11B2 gene (upper panels) and protein (lower panel) expression in in control and SF1-infected cells 8 days post-infection. Human adrenal cDNA and lysate were used as positive controls for RT-PCR and western blot, respectively. Overall, data from **A** and **B** showed a strong upregulation of HSD11B2 and a downregulation of HSD11B1 at the mRNA and protein levels. **C)** Schematic of strategies tested to revert the low cortisol/cortisone ratio in reprogrammed cells: i) forced-expression of HSD11B1, ii) forced-expression of Estrogen-related receptor alpha (ERRα), a TF known to increase CYP11B1 levels[5]; iii) inhibition of HSD11B2 with liquorice, a compound extracted from the root of the plant *Glycyrrhiza glabra*[6]. Moreover, co-cultures of hiSCs with fibroblasts and differentiated chromaffin cells were established, as data support the idea that chromaffin cells increase basal steroidogenic activity when co-cultured with adrenocortical cells[7]. **D)** RT-qPCR analyses of *STAR*, *CYP11B1* and *CYP11B2* expression levels, as well as cortisol measurements in urine-derived hiSCs overexpressing various combinations of SF1, HSD11B1 and ERRα or treated with liquorice for 8 days. **E)** mCherry-expressing PC12 cells were differentiated with nerve growth factor (NGF) and co-cultured with blue fluorescent protein-expressing human fibroblasts and GFP-expressing hiSCs (1:1:1 ratio) for 8 days before RT-PCR and RT-qPCR analyses of *STAR* expression and cortisol measurements. U, USCs; F, fibroblasts; P, PC12 chromaffin cells with (Diff.) or without NGF pre-treatment. Scale bars, left panels = 50 µm; bright field panel = 50 µm, inset = 20 µm; bottom right panel = 20 µm, inset = 5 µm. Data are represented as mean ± SEM.

Figure S5 (related to Figure 4)

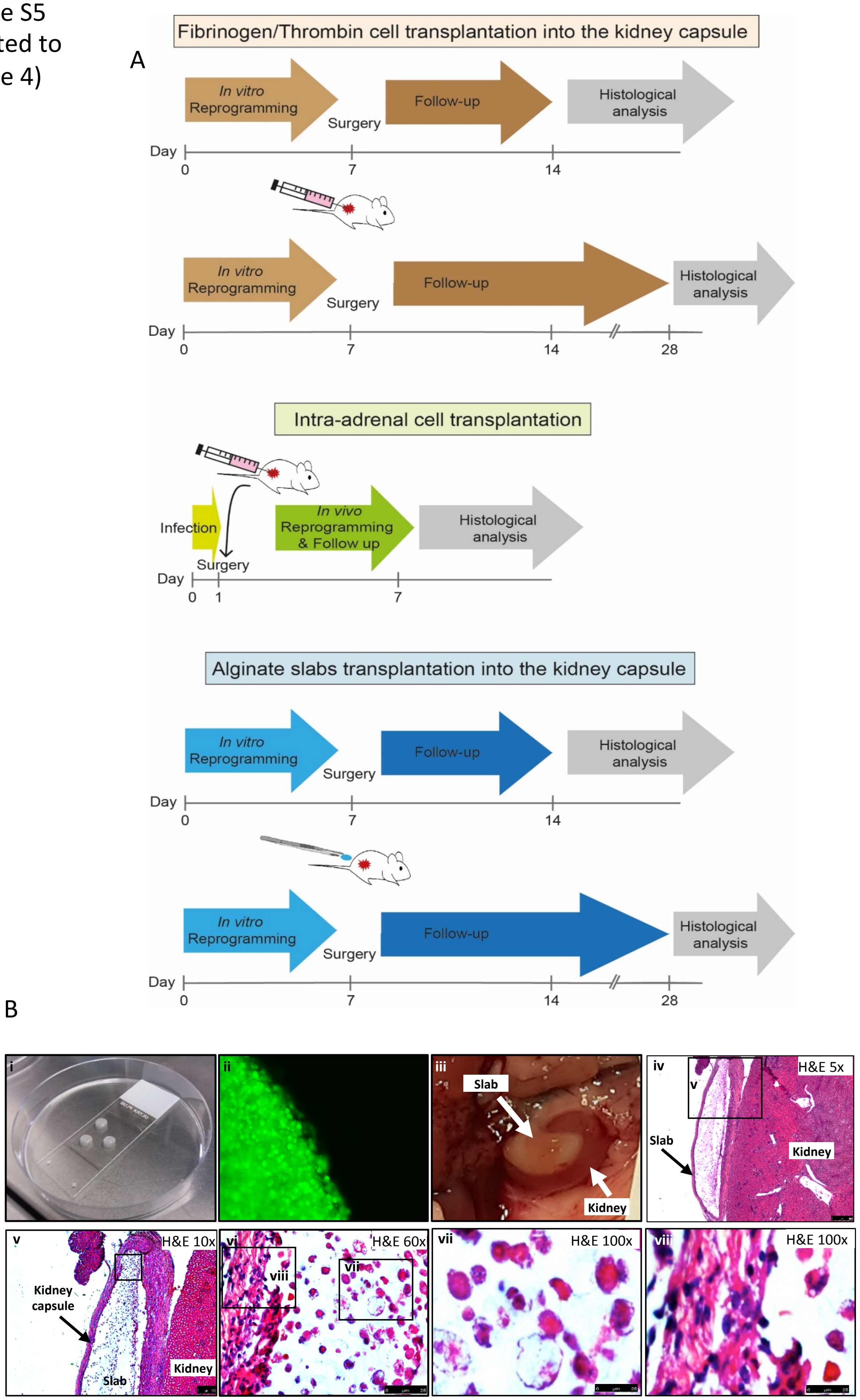

Figure Suppl. 5. A) Schematics of the *in vivo* transplantation experiments. Alginate slabs were implanted into C57BL/6 mice, while SCID mice were used for cells implanted into the kidney capsule and intra-adrenal. **B)** i) An example of alginate preparation with $1 \cdot 10^6$ hiSCs cells embedded within each slab; ii) An alginate slab containing GFP-positive hiSCs observed under fluorescence microscopy; iii) An alginate slab implanted under the kidney capsule; iii-viii) H&E analyses of alginate slabs sections after 1 week, with vi-vii showing histological features of cell death, not observed in adjacent structures such as the kidney capsule (viii). Scale bars: d = 500 µm; e = 100 µm; vi-viii = 25 µm.

Figure S6 (related to Figure 4)

A

Gene	Protein	Fold change	p-Val	Uniprot-Entry
CD9	CD9	2.6	$1.8x10^{-14}$	P21926
IF2B3	IGF2BP3	2.04	$2.3x10^{-11}$	O00425
SC16A	SEC16A	1.95	$2.0x10^{-10}$	O15027
TNR21	TNFRSF21	1.59	$1.7x10^{-06}$	
SYSC	SARS	1.59	$1.2x10^{-05}$	P49591
UN93B	UNC93B1	1.57	$1.8x10^{-06}$	Q9H1C4
DKK3	DKK3	1.51	$1.1x10^{-07}$	Q0UBP4
NUB1	NUB1	1.49	$2.7x10^{-07}$	Q9Y5A7
PO5F1	Oct4	1.49	$4.5x10^{-05}$	Q01860
SPR2A	SPRR2A	1.47	$1.0x10^{-07}$	P35326
CTGF	CTGF	1.46	$2.0x10^{-06}$	P29279
CXB1	GJB1	1.46	$6.1x10^{-08}$	P08034
TRI22	TRIM22	1.44	$1.1x10^{-05}$	Q81YM9
NAP1	C9orf156	1.43	$6.7x10^{-06}$	Q9BU70
NCOR2	NCOR2	1.42	$1.2x10^{-07}$	Q9Y618
LTOR1	LAMTOR1	1.42	$1.6x10^{-05}$	Q6IAA8
UBE2T	UBE2T	0.69	$1.9x10^{-05}$	Q9NPD8
EGNL	EGNL	0.67	$1.3x10^{-06}$	P17813

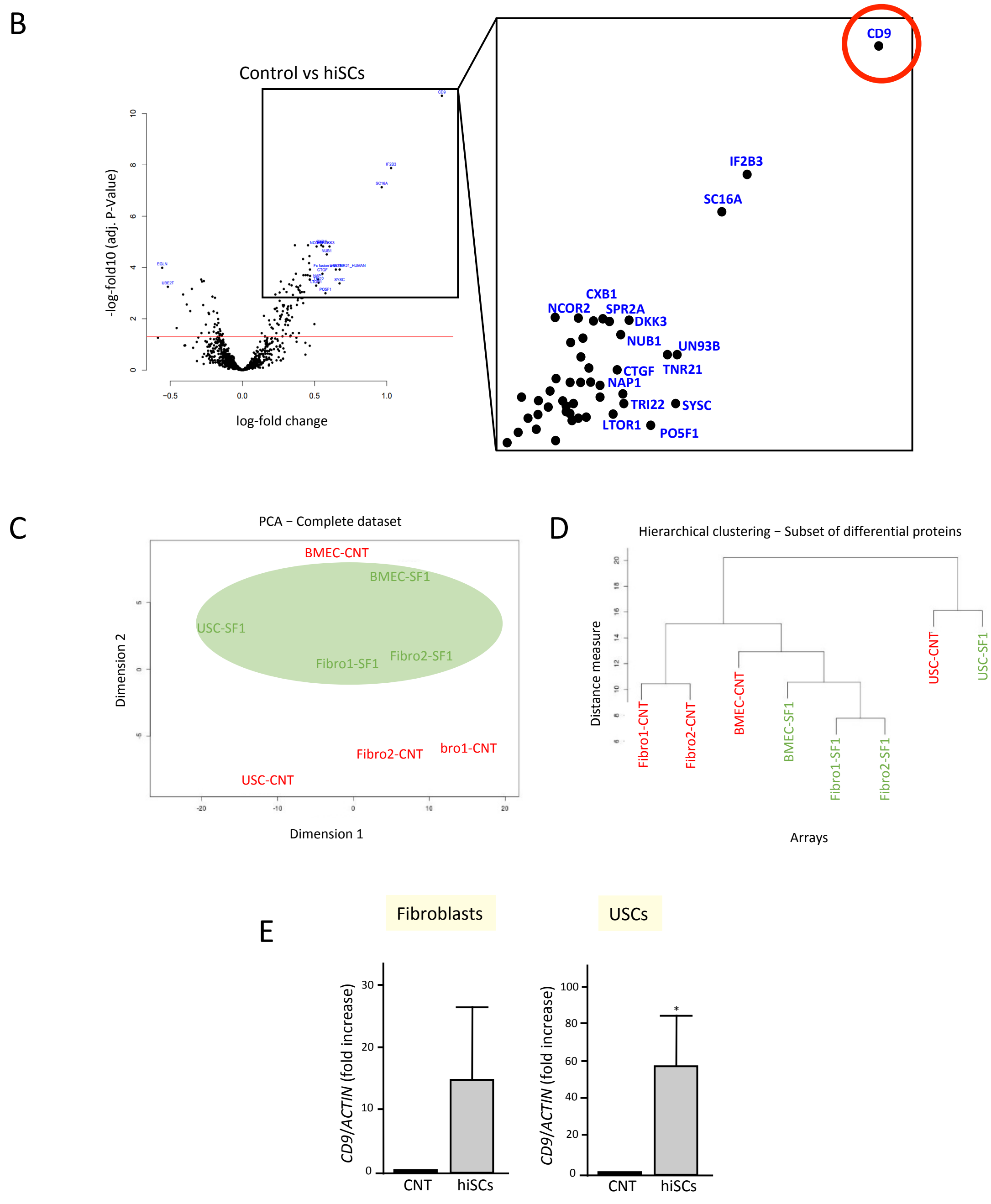

Figure Suppl. 6. Protein array of hiSC vs mock-reprogrammed cells.
A) Proteins with differential abundance in hiSCs and control cells. Proteins with a positive logFC value had a higher abundance in hiSCs, proteins with a negative value in control cells. Fold change, p values and Uniprot Identifiers are listed. **B)** Volcano plot representation showing the p values (adjusted for multiple testing) and corresponding log-fold changes. A significance level of adj. $p = 0.05$ is indicated as a horizontal line. The protein profiling of different human hiSCs showed upregulation of those involved in pathways controlling cellular reprograming such as protein transport and synthesis (IF2B3, SC16A,SYSC), cytoskeletal remodelling regulators (CD9, NUB1), embryonic reprogramming factors (DKK3,PO5F1), transcriptional regulators and chromatin remodelling proteins (NAP1, NCOR2). Interestingly, we detected a significant upregulation in hiSCs of LTOR1, a protein involved in cholesterol homeostasis. **C)** Principal-Component-Analyses (PCA) of the protein samples based on complete array data. **D)** Hierarchical clustering of the samples using complete array data. **E)** RT-qPCR validation of *CD9* up-regulation in USCs and fibroblasts before (control) and after (hiSC) reprogramming. Fold increase of *CD9* normalized by *ACTIN* levels. Data in **E** are represented as mean ± SEM.

Bibliography

1. Zhou, T. *et al.* Generation of human induced pluripotent stem cells from urine samples. *Nat Protoc* **7,** 2080–2089 (2012).
2. Geti, I. *et al.* A Practical and Efficient Cellular Substrate for the Generation of Induced Pluripotent Stem Cells from Adults: Blood-Derived Endothelial Progenitor Cells. *Stem Cells Transl. Med.* **1,** 855–865 (2012).
3. Schweitzer, K. M. *et al.* Characterization of a newly established human bone marrow endothelial cell line: distinct adhesive properties for hematopoietic progenitors compared with human umbilical vein endothelial cells. *Lab. Invest.* **76,** 25–36 (1997).
4. Solito, E., Romero, I. A., Marullo, S., Russo-Marie, F. & Weksler, B. B. Annexin 1 Binds to U937 Monocytic Cells and Inhibits Their Adhesion to Microvascular Endothelium: Involvement of the α4β1 Integrin. *J. Immunol.* **165,** (2000).
5. Li-Chuan Chenga, Tun-Wen Paib, L.-A. L. Regulation of human CYP11B1 and CYP11B2 promoters by transposable elements and conserved cis elements. *Steroids* **77,** 100–109 (2010).
6. Hammer, F. & Stewart, P. M. Cortisol metabolism in hypertension. *Best Pract. Res. Clin. Endocrinol. Metab.* **20,** 337–353 (2006).
7. Haidan, A. *et al.* Basal Steroidogenic Activity of Adrenocortical Cells Is Increased 10-Fold by Coculture with Chromaffin Cells [1]. *Endocrinology* **139,** 772–780 (1998).

Supplemental experimental procedures

Materials

Antibodies

Antibody	Host	Company	Code	Dilution	Application
StAR	Mouse	Abcam	Ab58013	1:2.000, 1:200	WB, IHC
SF1	Mouse	Invitrogen	434200	1:2.000	WB
HSD11B1	Rabbit	Abcam	Ab39364	1:2.000	WB, IF
HSD11B2	Mouse	Santa Cruz	Sc365529	1:2.000	WB, IF
HSD3B2	Rabbit	Avivasysbio	QC14296	1:2.000	WB
CYP11A1 (D8F4F)	Rabbit	Cell Signaling	sc-393592	1:1.000, 1:500	WB, IHC
CYP17A1	Rabbit (serum)	Gift from Prof Alan Conley, UC Davis		1:2.000	WB
GAPDH	Mouse	Santa Cruz	G-9	1:10.000	WB
ZO-1	Mouse	BD	610966	1:200	IF
Flag	Mouse	Sigma	F1804	1:5.000	WB
HA	Mouse	Sigma	H3663	1:5.000	WB
Myc	Mouse	Sigma	M4439	1:5.000	WB
V5	Mouse	Sigma	V8012	1:5.000	WB
TOM20	Rabbit	Santa Cruz	Sc-11415	1:500	WB
SULT2A1	Rabbit	abcam	Ab38416	1:500	WB
SF1 (A-1)	Mouse	Santa Cruz	sc-393592	1:200	IHC
GFP	Chicken	abcam	ab13970	1:200	IHC
Secondary RDye® 800 Mouse IgG	Goat	LICOR	925-32210	1:10.000	WB
Secondary RDye® 800 Rabbit IgG	Goat	LICOR	925-32211	1:10.000	WB
Secondary RDye® 800 Chicken IgG	Donkey	LICOR	925-32218	1:10.000	WB
anti-Rabbit IgG (H+L) AlexaFluor 568	Goat	Life technologies	A-11011	1:250	IF
anti-Mouse IgG (H+L) AlexaFluor 568	Goat	Life technologies	A-11004	1:250	IF
anti-Chicken IgY (H+L) AlexaFluor 488	Goat	Life technologies	A-11039	1:250	IF

Constructs: Human cDNAs encoding transcription factors SF1 (AB307718.1), Pbx1 (BT006705.1), WT1 (BC032861.2), Dax1 (BC011564.1) and Cited2 (AF109161.1) were subcloned into tagged vectors and after subcloned into pHIV-EGFP vector. pHIV-EGFP was a gift from Bryan Welm & Zena Werb (Addgene plasmid # 21373) (Welm et al., 2008). pHIV-EGFP-ERRα (NM_004451.4) was subcloned from pCMV flag ERRα. HSD11B1-FLAG was a gift from Moses Chao (Addgene plasmid # 24096) (Jeanneteau et al., 2008). pLV-Azurite and pLV-mCherry were a gift from Pantelis Tsoulfas (Addgene plasmid # 36086 and #36084, respectively). Cloning details are shown below:

**T. factor**	**Initial vector**	**Donor vector**	**Restriction sites used**	**Final plasmid**	**Primers used**
SF1	pCDNA4-hSF1	pT-Flag	EcoRI/BamHI	pFlag-SF1	FW: ggaattcATGGACTATTCGTACGAC
					RW: cgggatccTCAAGTCTGCTTGGCTTG
Pbx1	pAd/CMV/PBX1-IRES-nEBFP2	pCMVHA	EcoRI/XhoI	pCMVHA-Pbx1	FW: ggaattcggATGGACGAGCAGCCCAGG
					RW: ccgctcgagCTACTGTATCCTCCTGTC
WT1	pAd/WT1-IRES-nAmCyanto	pKMyc	XbaI/NheI	pKMyc-WT1	FW: gctctagaATGGAGAAGGGTTACAGC
					RW: ctagctagcTCAAAGCGCCAGCTGGAG
Dax1	pAd/CMV/DAX1-IRES-nEGFP	pENTR4-V5-2 (w234-1)	BamHI/XbaI	pENTR4-V5-Dax1	FW: cgggatccATGGCGGGCGAGAACCAC
					RW: gctctagaTTATATCTTTGTACAGAG
Cited2	SB52	pCMVHA	EcoRI/XhoI	pCMVHA-Cited2	FW: ggaattcggATGGCAGACCATATGATG
					RW: ccgctcgagTCAACAGCTCACTCTGCT
Flag-SF1	pFlag-SF1	pHIV-EGFP	XbaI/BamHI	pHIV-EGFP-Flag-SF1	FW: gctctagaAGCACCATGGATTACAAA
					RW: cgggatccTCAAGTCTGCTTGGCTTG
HA-Pbx1	pCMVHA-Pbx1	pHIV-EGFP	XbaI/BamHI	pHIV-EGFP-HA-Pbx1	FW: gctctagaACCATGTACCCATACGAT
					RW: cgggatccCTACTGTATCCTCCTGTC
Myc-WT1	pKMyc-WT1	pHIV-EGFP	XmaI/BamHI	pHIV-EGFP-Myc-WT1	FW: cgttaacATGGAACAGAAACTCATC
					RW: tcccccgggTCAAAGCGCCAGCTGGAG
V5-Dax1	pENTR4-V5-Dax1	pHIV-EGFP	HpaI/XbaI	pHIV-EGFP-V5-Dax1	FW: cgttaacTCCACCATGGAAGGTAAG
					RW: gctctagaTTATATCTTTGTACAGAG
HA-Cited2	pENTR4-V5-Dax1	pHIV-EGFP	XbaI/BamHI	pHIV-EGFP-HA-Cited2	FW: gctctagaACCATGTACCCATACGAT
					RW: cgggatccTCAACAGCTCACTCTGCT
Flag-ERRα	pCMV flag ERR alpha	pHIV-EGFP	XbaI/XbaI	pHIV-EGFP-Flag-ERRα	FW: gctctagaaccATGGATTACAAGGATGAC
					RW: ctgctctagaTCAGTCCATCATGGCCTC

pT-FLAG was a gift from Yegor Vassetzky (Addgene plasmid # 31385) (Dmitriev and Vassetzky, 2008)

pAd/CMV/PBX1-IRES-nEBFP2 was a gift from Edward McCabe (Addgene plasmid # 29755)

pAd/WT1-IRES-nAmCyan was a gift from Edward McCabe (Addgene plasmid # 29756)

pKMyc was a gift from Ian Macara (Addgene plasmid # 19400) (Joberty et al., 2000)

pAd/CMV/DAX1-IRES-nEGFP was a gift from Edward McCabe (Addgene plasmid # 29752)

pENTR4-V5-2 (w234-1) was a gift from Eric Campeau (Addgene plasmid # 17426) (Campeau et al., 2009)

SB52 was a gift from Shoumo Bhattacharya (Addgene plasmid # 21487) (Bhattacharya et al., 1999)

pCMV flag ERR alpha was a gift from Toren Finkel (Addgene plasmid # 10975) (Ichida et al., 2002)

Primers: PCR and qPCR primers (Sigma Aldrich)

Gene	Primer FW	Primer RW
Flag-SF1	AGCACCATGGATTACAAA	AGAAGCCCTTGCAGCTCTC
HA-Pbx1	AGAAGCCCTTGCAGCTCTC	GCCTGCGCCTCATCCAAACT
Myc-WT1	CAGAAACTCATCTCTGAAGAG	AGCTGTCGGTGGGGGTGTGGC
V5-Dax1	CTATCCCTAACCCTCTCCTCG	AGCGCCACGTTCCGCCCGCCC
HA-Cited2	AGAAGCCCTTGCAGCTCTC	TGTTGCCCGCGCCGTAGTGTA
STAR	AAGAGGGCTGGAAGAAGGAG	TCTCCTTGACATTGGGGTTC
CYP11A1	AGACCTGGAAGGACCATGTG	TCCTCGAAGGACATCTTGCT
HSD3B2	GCCTGTTGGTGGAAGAGAAG	GCAGGCTCTTTTCAGGAATG
CYP21A2	TGGACGTGATTCCCTTTCTC	CACCCCTTGGAGCATGTAGT
CYP17A1	GTGGAGACCACCACCTCTGT	CAGCAGGAGGAGACGGTTAC
CYP11B1	GGCAGAGGCAGAGATGCTG	TCTTGGGTTAGTGTCTCCACCTG
CYP11B2	GGCAGAGGCAGAGATGCTG	CTTGAGTTAGTGTCTCCACCAGGA
HSD17β	GTCCACTTGAGCCTGATCG	GCGAAAGACTTGCTTGCTGT
HSD11B1	AAGCAGAGCAATGGAAGCAT	GAAGAACCCATCCAAAGCAA
HSD11B2	TCATCACCGGCTGTGACTC	GGGGCTGTTCAACTCCAATA
MC2R	AGTTCCTGCTTCAGAGCTG	CTTGCTGTGTTGTTGATG
MRAP	GCCTCTGCCCCATACTACAG	CGGACCAGGACATGTAGAGC
Actin	AGAGCTACGAGCTGCCTGAC	AGCACTGTGTTGGCGTACAG
GAPDH	TGCACCACCAACTGCTTAG	GGATGCAGGGATGATGTTC
PPARγ	TCTGGCCCACCAACTTTGGG	CTTCACAAGCATGAACTCCA
LPL	GAGATTTCTCTGTATGGCACC	CTGCAAATGAGACACTTTCTC
Osteocalcin	ACACTCCTCGCCCTATTG	GATGTGGTCAGCCAACTC
ALP	CACGGGCACCATGAAGGAAAAG	TGGCGCAGGGGCACAGGAGACT
Runx2 [a]	GCACAGACAGAAGCTTGAT	CCCAGTTCTGAAGCACCT

Aggrecan	TACTCTGGGTTTTCGTGACTC	CGATGCCTTTCACCACGACTT
Col-2	GCCTGGTGTCATGGGTTT	GTCCCTTCTCACCAGCTTTG
Sox-9	GTACCCGCACTTGCACAAC	TCTCGCTCTCGTTCAGAAGTC
SF1 endog	GAGAGCCAGAGCTGCAAGAT	CTTGTACATCGGCCCAAACT
ERRα endog	CTATGGTGTGGCATCCTGTG	GCACTCCCTCCTTGAGCAT
Pbx1 endog	CAGATGCAGCTCAAGCAGAG	CTCTTTGGCTTCCTCACTGG
WT1 endog	CAGGCCAGGATGTTTCCTAA	AATGAGTGGTTGGGGAACTG
Dax1 endog	CCAAGCCATCAAGTGCTTTC	ATTTGCTGAGTTCCCCACTG
Cited2 endog	CAAAAACGGAAGGACTGGAA	TGTATGTGCTCGCCCATTAG
Pax6	GCCAGCAACACAGCTAGTCA	TGTGAGGGCTGTGTCTGTC
TH	CCGTGCTAAACCTGCTCTTC	ATGGTGGATTTTGGCTTCAA
12S Mito	GCTCGCCAGAACACTACGAG	CAGGGTTTGCTGAAGATGGCG
ESR1	TGGAGATCTTCGACATGCTG	TCCAGAGACTTCAGGGTGCT
DMRT1	GTCATGAGGCACGGGTTACT	TGGAGATCTTCGACATGCTG
CYB5	TTCAGAAGCACAACCACAGC	AACTTCTTCCCCACCAGGAT
SULT2A1	TGGTTTGACCACATTCATGG	GGGCCACTGTGAAGTGATTT
LHR	CAGCCACTGCTGTGCTTTTA	CACTCTCAGCAAGCATGGAA

[a] These primers detect both A and B isoforms.

Cell culture

Urine-derived stem cells (USCs)

Urine was collected in sterile containers and processed as soon as possible under a sterile tissue culture cabinet. Time from urine collection to processing varied from 1 min to 30 mins. Urine was transferred into sterile 50-ml tubes and centrifuged at 400g for 10 min at room temperature and the supernatant was aspirated. Pellets were gently resuspended and washed with Phosphate Buffered Saline (PBS, Sigma) supplemented with 100 U ml $^{-1}$ of penicillin (Sigma), 100 μg ml $^{-1}$ of streptomycin (P/S) (Sigma) and 500 ng ml $^{-1}$ of amphotericin B (Sigma). Samples were centrifuged at 200 g for 10 min at room temperature and resuspended in 12 ml of primary medium (DMEM/high glucose and Ham's F12 nutrient mix (1:1), supplemented with 10% (vol/vol) Foetal Bovine Serum and P/S (Sigma) supplemented with the REGM SingleQuot kit supplements (CC-4127, Lonza) and 2.5 μg ml $^{-1}$ amphotericin B (Sigma) and then 1 ml was transferred into each well of a 12-well plate. 1 ml of

primary medium was added to the culture daily for the next 3 days and then medium was changed to proliferation medium (REBM medium (Lonza, CC-3191) supplemented with 10% (vol/vol) FBS, 1% P/S and the REGM SingleQuot kit supplements. Cells were cultured in a designated humidified 37°C incubator with 5% CO_2. Appearance of colonies was checked daily from day 4. Type-II colonies were passaged into T75 flasks and then processed for storage in liquid nitrogen or further amplified for CD-classification through flow cytometry or experiments.

Fibroblasts

Fibroblast isolation was performed as described previously (Poliandri et al., 2017). Briefly, a 4 mm punch biopsy from arm was obtained with a biopsy punch (Stiefel, SmithKline Beecham Ltd, Slough, UK) placed immediately into 15 ml-falcon containing isolation medium (DMEM supplemented with 10% (vol/vol) FBS and 1% P/S). Skin sample was transferred in a 10-dish with digestion media (DMEM/high glucose, 20%FBS (vol/vol), 0.25% collagenase type-I, 0.05% DNAse-I and 1% P/S –all from Sigma-), chopped into 1 square mm cubes and placed at 37°C tissue culture incubator overnight in a 15 ml-falcon tube with digestion media. Samples were centrifuged and pellet resuspended in 5 ml of isolation medium before plating in gelatin coated T25 flasks. Cells were kept in human fibroblast media (DMEM/High glucose with sodium pyruvate and L-glutamine, 20% FBS (vol/vol) and 1% P/S).

Late outgrowth endothelial progenitor cells (L-EPCs)

L-EPCs were isolated as described previously (Martin-Ramirez et al., 2012). Briefly, 25-50 ml of blood were diluted 1:1 in PBS in a 50 ml tube. 10 ml of diluted blood was placed on top of 4 ml of Ficoll (GE Healthcare) in 15 ml falcon tubes and centrifuged at 1000 g for 20 min without brake. The buffy coat formed was collected, washed with PBS and centrifuged at 540 g for 7 min with brake. Supernatant was discarded and pellet resuspended in 5 ml of culture medium (EGM-2 Bullekit, Lonza, CC-3162).

Bone marrow endothelial cells (BMEC)

BMEC were a kind gift of Dr Egle Solito (Schweitzer et al., 1997; Solito et al., 2000), Queen Mary University, London.

HEK293T

HEK293T were cultured with DMEM, 10% FBS and 1% P/S.

Lentiviral production

Lentiviral particles were prepared using 90% confluent HEK293T cells. 1 hour before transfection medium was replaced to serum-free DMEM. Cells were transfected with pHIV-EGFP vector (with the ORF of interest) together with the packaging vectors pCMVdR8.2 and pMD2.G using polyethileneamine reagent (PEI, Warrington, USA). 2 hours after transfection medium was replaced to DMEM, 10%FBS (vol/vol), 1% P/S. Medium was collect after 24 and 48 hours, filtered and ultracentrifuged at 50.000 g for 1 hour at 16°C. The resulting pellet was resupended in PBS, aliquoted and immediately stored at -80°C.

Precision LentiORF SF1 (NR5A1) lentiviral particles were purchased from Dharmacon (vector pLOC-SF1). The vector encoding SF1 is driven by the CMV promoter. pLOC vector also contains a nuclear localized TurboGFP™ (Evrogen™, Moscow, Russia) and a blasticidin S resistance for selection.

Cell reprogramming

60.000 cells/well of a 6-well plate were infected with lentiviral particles at a MOI=200 with 8μg/ml of polybrene (Millipore, TR-1003-G). Medium was replaced after 12 hours and treatments with different molecules were added after 2 days. Cells were cultured for additional 5 to 10 days prior to analysis. Concentrations used to treat cells are as follows: 8-bromo-cyclic AMP (8-br-cAMP), 100 μM (Sigma, B5386); db-cAMP, 200μM (Sigma, D0260); ACTH, 1μM (Sigma A0423); Angiotensin II human, 10 μM (Sigma, A9525); Retinoic acid (ATRA), 10 μM (Sigma, R2625); KCl, 16 mM (Sigma, P9541); Licorice (Sigma, G2137); Bombesin, 0,5 μg/ml (Bachem, H-2155); WNT4, 100 ng/ml (R&D Systems, 6076-WN-005), LH alpha/beta heterodimer, 100 ng/ml (R&D Systems, 8899-LH-010), D-TRP6-LHRH, 1μM. Triptorelin (D-TRP6-LHRH) was a kind gift of Prof. Andrew Schally.

Gene expression analysis

RNA was extracted from human adrenals (adjacent to Conn's) and RNA from ovary and testis was purchased from Amsbio (CR560139) and Takara (636533), respectively.

Messenger RNA was purified with RNeasy Mini Kit (Qiagen, 74106) using the RNase-Free DNase Set (Qiagen, 79254) to eliminate gDNA contamination. 10-500 ng of mRNA were incubated with 60ng/μl

of random primers 5 min at 70°C followed by incubation with 500 μM dNTPs, 40 U of RNAse inhibitor, 200 U of M-MuLV RT and 1X M-MuLV buffer for 10 min at 25°C, 90 min at 42°C and 15 min at 70°C to generate cDNA. All products were purchased from NEB. Samples were diluted to a final concentration of 2 ng/ml and 2 ng of cDNA were used for qPCR experiments.

Standard PCR experiments were performed using Taq DNA polymerase (NEB, M0273) and SYBR FAST Universal Kit (KAPA Biosystems, KK4602) for qPCR experiments.

qRT-PCR was performed on a Stratagene Mx3000P thermocycler using KAPA SYBR fast Universal Kit (KAPA Biosystems, KK4602) with 500 nM forward and reverse primers. Data were analyzed using MxPro software (Stratagene, Stockport, UK). Relative quantification analysis was performed following the $2^{-\Delta\Delta CT}$ method (Livak and Schmittgen, 2001) and data was normalized to Actin expression.

Immunocytochemistry

Control USCs or USCs differentiated for 8 days were fixed using 4% paraformaldehyde in PBS for 20 min on ice. Permeabilization was performed using PBS containing 0.02% saponin for 7 min and 10 mM glycine containing 0.01% saponin for 15 min. Cells were blocked for 1 h with PBS containing 0.01% saponin, 10 mM glycine and 5% (w/v) BSA before incubation with primary antibody (dilution 1:300) in buffer A (PBS containing 0.01% saponin and 1% BSA) overnight. Proteins were incubated 45 min with the corresponding Alexa Fluor® secondary antibodies (Thermo Fisher Scientific) diluted 1:400 in buffer A and nuclei were stained using Hoechst 33259 (Invitrogen). Images were obtained with a fluorescence microscope Zeiss Axio Vert.A1 equipped with a AxioCam MRm camera.

Immunohistochemistry

Specimens from in vivo transplantation experiments were either embedded in paraffin or in Optimal Cutting Temperature (OCT) compound.

Paraffin embedding: Mouse adrenals/kidneys were fixed in 4% PFA (Acros Organics, 416780010) overnight at 4°C and dehydrated in a series of ethanol washes, 50%, 70%, 90% and 100% for 1 hour each on a rotating plate. After two incubations with Xylene (Fischer Scientific, X/0250/17) for 5 and 10 minutes adrenals were placed in a container with melted paraffin (VWR, 361077E) overnight at 56°C and finally placed in embedding cassettes (VWR, 18000-244) filled with melted paraffin. Paraffin blocks were cut at 6-8μm using a rotary microtome (Thermo scientific, 902100A) and sections

transferred on superfrost plus glass slides (VWR, 48311-703) covered with water. Sections were left on the slides on a hotplate (Thermoscientific, E181SL) at 56°C for 30-60 minutes. Once sections were flat, excess water was removed and sections were allowed to dry at room temperature.

Sucrose-cryopreservation and OCT-embedding: Mouse adrenals/kidneys were fixed in 4% PFA overnight at 4°C, followed by PBS wash for 1 hour. The following day specimens were incubated in filtered 30% sucrose solution (Fisher Scientific, S/8560/60) overnight. Finally they were transferred in a container filled with liquid OCT (VWR, 361603E), orientated and placed on dry ice until OCT solidified. OCT embedded samples were cut at 14-18μm using a cryostat (Leica GM1510S) and placed on superfrost plus glass slides. Sections were incubated at room temperature overnight and then stored at -80°C.

Fluorescent Immunohistochemistry on fresh frozen sections: Sections were fixed in 4% PFA for 15 minutes on ice, blocked with 10% normal goat serum (Sigma-Aldrich, G9023) in PBS-0.1% Triton for 1 hour and incubated overnight with anti-GFP antibody diluted in PBS at room temperature. Slides were washed and then incubated with secondary antibody and DAPI (1:1000) in PBS-Triton and mounted with glass cover slips using PBS:Glycerol (Sigma-Aldrich, G5516) solution at a ratio of 1:3. Slides were visualized using a Leica DM5500B automated upright microscope and stored at 4°C.

Chromogenic Immunohistochemistry with DAB on paraffin sections: Sections were deparaffinised in Xylene incubations, washed in 100% Ethanol and reacted with 3% H2O2 (Sigma Aldrich, 21,676-3) diluted in Methanol (Fischer Scientific, M/4000/PC17) for 30 minutes at room temperature to block endogenous peroxidase activity. Sections were rehydrated in decreasing concentrations of Ethanol (100%, 90%, 70% and 50%) for 10 minutes each, followed by incubation in water for 10 minutes and washes in PBS-0.1% Triton. After blocking and overnight incubation with primary antibody as above, slides were incubated with biotinylated secondary antibody for 2 hours at room temperature followed by 1 hour incubation with Avidin-Biotin Complex (ABC) (Vector labs, PK-6100). Slides were developed with DAB (Vector labs, SK-4105) and the reaction was stopped by placing slides in water. Slides were dehydrated in increasing concentrations of ethanol (50%, 70%, 90% and 100%), xylene

and mounted using Vectamount mounting medium (Vector labs, H-5000). Slides were visualized using a Leica DM5500B automated upright microscope equipped with a DFX365 FX camera.

Hematoxylin and Eosin staining: Sections were incubated with Hematoxylin Solution Gill No.3 (Sigma, GHS332) solution for 2 minutes, washed and incubated in 1% acid alcohol (1% Hydrochloric acid (Fisher Scientific, A481-212) in 70% Ethanol) for 1 minute. Sections were dipped in 0.2% ammonia solution (Sigma-Aldrich, 320145) diluted in distilled water, washed and incubated in 80% Ethanol for 1 minute followed by Eosin (National diagnostics, HS-402) incubation for 15 seconds. Sections were further dehydrated by incubating them in 95% Ethanol and 100% Ethanol for 1 minute each followed by 2 Xylene incubations for 3 minutes each. Following staining and dehydration steps, sections were mounted with Vectamount (Vector, H-5000).

Western Blot

Immunoblot analysis was performed as described previously (Rodríguez-Asiain et al., 2011). Briefly, SDS-PAGE was used to size-separate proteins followed by transfer to nitrocellulose membranes (Whatman, UK). After blocking membranes with PBS containing 0,1% Tween-20 and 5% skimmed milk, primary antibody was added over-night. Proteins were detected using secondary fluorescent antibodies (LICOR) and visualized using LICOR Odyssey Scanner 2.2.

Hormone quantification

USCs cell supernatant was collected 8 days after reprogramming. Quantification of metabolites was achieved by mass spectrometry. Cortisol was also measured using cortisol ELISA Kit (Abcam, ab154996) according to the manufacturer's instructions.

Calibrator, Internal Quality Control and Internal Standard solutions: Individual stock solutions for all analytes and internal standards (Is) were prepared in methanol (each 1000 mg/L). These were then used to prepare combined working solutions containing all analytes for calibration and internal quality control (IQC) purposes. To make these, appropriate volumes of each stock solution were added to a glass tube and then dried down under nitrogen at 60°C. The steroids were then reconstituted in methanol to create calibrator and IQC working solutions each containing: DHEAS (2000 μg/mL),

cortisol (200 µg/mL), 17-hydroxypregnenolone (160 µg/mL) 17-hydroxyprogesterone (120 µg/mL), androstenedione (80 µg/mL), pregnenolone, corticosterone, 11-deoxycortisol, 21-deoxycortisol, cortisone (each at 40 µg/mL), testosterone (8 µg/mL) and 11-deoxycorticosterone (4 µg/mL). The working solutions were each further diluted in methanol to create three further working solutions as follows: 3+20 (v/v), 1+39 (v/v) and 1:199 (v/v). All four working solutions were then used to make appropriate volumes of calibration standard/IQC solution by dilution in DMEM. After thorough mixing and equilibration (24 h, 2–8 °C), calibrators and IQC solutions were portioned (250 µL) in 1.5 mL microcentrifuge tubes (Eppendorf, Stevenage, UK) and stored at -20 °C until required. A combined IS sub-stock solution was prepared in methanol containing deuterated steroids at the following concentrations: DHEAS-D2 (25000 µg/mL), cortisol-D4 (2500 µg/mL), 17-hydroxypregnenolone-D3 (2000 µg/mL) 17-hydroxyprogesterone-D8 (1500 µg/mL), androstenedione-D7 (1000 µg/mL), pregenenolone-D4, corticosterone-D8, 11-deoxycortisol-D2, 21-deoxycortisol-D8, cortisone-D2 (each at 500 µg/mL), testosterone-D3 (100 µg/mL) and 11-deoxycorticosterone-D8 (50 µg/mL). The IS working solution was freshly prepared before each batch by dilution of 50 µL of the IS sub-stock stock solution in acetonitrile to 20 mL in a volumetric flask.

Specimen processing: Portions of frozen calibrators, IQC solutions and unknown media samples were thawed and mixed at room temperature by inversion before analysis. Aliquots (250 µL) of unknown media samples were then transferred into 1.5 mL micro-centrifuge tubes. Subsequently, 250 µL of IS working solution and 500 µL of ice-cold acetonitrile was added to each sample. Tubes were capped and thoroughly vortex-mixed for 30 seconds. Precipitated protein was then pelleted by centrifugation (12,000 rpm, 10 min) and the remaining supernatant transferred to a 10 mL glass tube containing 300 µL of deionised water (dH2O). Ethyl acetate (1 mL) was added and the tube contents vortex-mixed (5 min). Following a brief centrifugation (1,000 rpm, 1 min) to clarify aqueous and organic layers, the top organic layer was removed to a clean tube using a glass pipette. The extract was then evaporated to dryness under nitrogen at 60°C, re-constituted in 200 µL of a 65+35 (v/v) mixture of dH_2O:methanol and transferred to a glass insert autosampler vial.

LC-MS/MS procedure: Extracts were injected (100 µL) onto the LC column at a flow rate of 0.40 mL/min. Mobile phases were (A) dH2O containing 0.1% (v/v) formic acid and (B) methanol

containing 0.1% (v/v) formic acid. The column was maintained at 40°C. LC instrument control was performed using Aria MX (Version 1.1, ThermoFisher Scientific).

MS/MS was carried out in positive mode using atmospheric pressure chemical ionisation (APCI; needle discharge current 5 μA; temperatures: vaporiser 500 ºC; capillary 400 ºC; auxiliary and sheath gases 20 and 5 (arbitrary units) respectively. Data were collected in high-resolution (0.40 m/z full width at half maximum - FWHM), selected reaction monitoring (SRM) mode, with two m/z transitions per analyte and one m/z transition for each internal standard. MS instrument control and data acquisition were performed using Xcalibur (version 2.2 SP1.48, ThermoFisher Scientific). Post-analysis processing was carried out using LC QuanTM (version 2.6, ThermoFisher Scientific). For assay calibration, peak area ratios (analyte quantifier to IS) were used to construct calibration graphs, with lines fitted by linear regression. The intercepts were not forced through zero, and line weighting was applied (1/concentration). Deuterated ISs were used for all steroids in the developed method.

Flow cytometry

$5x10^5$ USCs at passage 2 were harvested and resuspended in 100 μl of PBS containing 1,5% BSA, 5% FBS and 0,5% Na-Azide and were incubated for 30 min on ice with the following antibodies purchased from R&D biosystems:

**Antibody**	**Specie**	**Dilution**	**Code**
Anti-CD29	Mouse	1:10	561795
Anti-CD44	Mouse	1:10	560977
Anti-CD54	Mouse	1:10	560971
Anti-CD73	Mouse	1:10	561014
Anti-CD105	Mouse	1:40	560839
Anti-CD146	Mouse	1:10	561013
Anti-CD166	Mouse	1:10	560903
Anti-SSEA-4	Mouse	1:10	560126
Anti-CD31	Mouse	1:10	560984
Anti-CD34	Mouse	1:10	560942
Anti-CD45	Mouse	1:10	560976
FITC-Isotype CNT	Mouse	1:10	555748
PE-Isotype CNT	Mouse	1:10	551436

Samples were analyzed in LSR Fortessa Flow Cytometer (BD Biosciences).

Differentiation of USCs into mesenchymal lineages and analysis.

USCs at passage 2 were grown in proliferation medium until confluent. 48 hours later medium was changed to adipogenic (DMEM 10% FBS, 10 ng/ml insulin, 500 mM 3-isobutyl-1-methylxanthine, 1 mM dexamethasone, 1 mM Rosiglitazone), chondrogenic (DMEM 10% FBS, 0.1 mM dexamethasone, 10 ng/ml transforming growth factor (Tgf) β1 (R&D Systems), insulin-transferrin-selenium (ITS) (Life Technologies), and 50 mg/ml ascorbate) and osteogenic (DMEM 10% FBS, 0.1 mM dexamethasone, 100 mg/ml ascorbate , 10 mM β-glycerophosphate) media for 21 days, while controls were kept in proliferation media. Medium was changed twice a week. Cells were stained with Oil Red O (adipogenic lineage), Alcian Blue (chondrogenic linage) and Alizarin Red (osteogenic medium) as reported previously (Guasti et al., 2012). Images were taken using Zeiss Axio Vert.A1 inverted microscope equipped with an Axiocam 105 camera.

Telomerase activity assay

$2x10^4$ L-EPCs, fibroblasts and USCs at passage 2 from different donors were harvested and assayed using a Telo TAAGG ELISA kit (Roche, 11854666910) according to the manufacturer's instructions. HEK293T cells were used as a positive control of the assay.

Animal experiments

Ethics Statement: All animal experiments were performed in strict accordance with animal protocol, approved by the ethical board of Landesdirektion Sachsen, Germany (protocol N: DD24-5131/354/28).

Mice: Female 8 weeks old C57BL/6 (http://www.criver.com/files/pdfs/rms/c57bl6/rm_rm_d_c57bl6n_mouse.aspx) and SCID (http://www.criver.com/products-services/basic-research/find-a-model/fox-chase-scid-mouse) mice were obtained from Charles River Laboratory. Mice were maintained under 12:12 h light/dark cycle and fed ad libitum. After the arrival the mice were given 3-5 day acclimatization period before beginning of the experiments.

Mice were anesthetized by ketamine/xylazine (100mg/kg Ketamine und 10 mg/kg xylazine).

For cell transplantation under the kidney capsule of SCID mice a longitudinal incision was made with fine scissors in the abdominal skin. A 3-mm incision was made in the kidney capsule, which was

carefully separated from the kidney cortex, and cells in alginate or fibrin clot were inserted into the formed pouch. The same procedure with repeated with the second kidney.

Cell encapsulation in alginate: Cells were mixed with 25 µl of 3 % UP-MVG alginate (Novamatrix), dissolved in Custodiol-HTK solution (H.S. Pharma) and polymerized in 70 mM SrCl2 for 15 min. The thickness of the alginate/cell slab was 550 µm. Just before the transplantation, each slab was cut in halves. One half was transplanted under each kidney capsule of C57BL/6 mice.

Cell transplantation in fibrin clot. Thrombin and Fibrinogen were obtained in Sigma-Aldrich. Cells were mixed with 10 µl of Thrombin (10 u/ml). 10 µl of Fibrinogen (10 mg/ml) was added to the cell/Thrombin mixture and immediately placed in the pouch under kidney capsule of SCID mice.

Intraadrenal transplantation. A short longitudinal incision was made on the left lateral body wall to open the retroperitoneal space. The left adrenal was exposed, and $5*10^5$ cells concentrated in a total volume of 10 µL were pipetted directly into the adrenal through a capillary tip.

Animals were monitored for up to 3 weeks. Then they were euthanized by cervical dislocation, kidneys or adrenals were removed and collected for immunohistochemical analysis.

Collection of urine samples from donors

This study has been performed under the ethical approval NHS REC form reference: 13/LO/0224. All patients involved were previously informed and consent forms were obtained prior to analysis of samples. For patient #2 clinical and biochemical diagnosis of congenital adrenal hyperplasia due to a presumptive 3B hydroxysteroid dehydrogenase mutation was made on the basis of: a raised serum DHEAS; failure of a clinical response to ovarian suppression for hirsutism and urine gas chromatography demonstrating raised excretion rates of 3 beta-hydroxy-5-ene steroids i.e. dehydroepiandrosterone and low cortisol metabolite output - tetrahydrocortiosone (THE) and tetrahydrocortisol (THF).

Sequencing

PCR products were purified using QIAquick PCR Purification Kit (Qiagen) and Sanger sequencing was performed by GATC Biotech (Germany). Sequences were analyzed using Chromas 2.6.2 software.

Cell counting assay.

Cell Counting Kit-8 (Sigma) was used to determine the viability of cells in culture according to the manufacturer's instructions.

Establishment of inducible cell lines

SparQ cumate switch pCDH-CuO-MCS-IRES-GFP-EF1-CymR-T2A-Puro All-in-one inducible lentivector (QM812B-1) was purchased from Cambridge Bioscience Ltd, SF1 was subcloned using primers FW ctagctagcaacATGGACTATTCGTACGAC (NheI) and RW gcatcgttcgaaTCAAGTCTGCTTGGCTTG (BstBI) and lentiviral particles were generated as described previously. USCs P2 were infected with lentiviral particles (MOI 50) and stable cell lines were established after two weeks in REBM medium containing 1 mg/ml puromycin. Different concentrations of cumate solution 1000x (QM100A-1, Cambridge Bioscience Ltd) were added to the medium before analysis.

Protein array of human reprogrammed steroidogenic cells

Protein array was performed by Sciomics GmbH (Germany). USCs, fibroblasts and BMEC at P2 were reprogrammed to steroid-producing cells for 8 days.

Protein extraction. Samples were extracted with scioExtract buffer (Sciomics) using the extraction SOPs and the bulk protein concentration was determined by BCA assay.

Label reaction. Samples were labelled at an adjusted protein concentration of 1 mg/mL for one hour with scioDye1 and scioDye 2. After one hour, the reaction was stopped by the addition of hydroxylamine. Excess dye was removed 30 min later and the buffer exchanged to PBS. Samples were analysed in a dual-colour approach using a reference-based design on ten scioDiscover antibody microarrays (Sciomics) targeting 900 different proteins with more than 1,000 antibodies. Each antibody is represented on the array in four replicates. The arrays were blocked with scioBlock (Sciomics) on a Hybstation 4800 (Tecan, Austria) and afterwards the samples were incubated competitively using a dual-colour approach. After incubation for three hours, the slides were thoroughly washed with 1x PBSTT, rinsed with 0.1x PBS as well as with water, and subsequently dried with nitrogen.

Data acquisition and analysis. Slide scanning was conducted using a Powerscanner (Tecan, Austria) with identical instrument laser power and adjusted PMT settings. Spot segmentation was performed with GenePix Pro 6.0 (Molecular Devices, Union City, CA, USA). Acquired raw data were analysed

using the linear models for microarray data (LIMMA) package of R-Bioconductor after uploading the median signal intensities.

For normalisation, a specialised invariant Lowess method was applied. For analysis of the samples a one-factorial linear model was fitted with LIMMA resulting in a two-sided t-test or F-test based on moderated statistics. All presented p-values were adjusted for multiple testing by controlling the false discovery rate according to Benjamini and Hochberg. Proteins were defined as differential for an adjusted p value < 0.05 and -0.5 < logFC < 0.5. For cluster analysis log ratios of signal intensities of sample and reference were used (M-value). Differences in protein expression between different samples or sample groups are presented as log fold changes (logFC) calculated for the basis 2. In a study comparing samples versus control a logFC = 1 means that the sample group had on average a 21 = 2 fold higher signal as the control group. logFC = -1 stands for 2-1 = 1/2 of the signal in the sample as compared to the control group.

Resource Table

REAGENT or RESOURCE	SOURCE	IDENTIFIER
**Antibodies**		
StAR	Abcam	Ab58013
SF1	Invitrogen	434200
HSD11B1	Abcam	Ab39364
HSD11B2	Santa Cruz	Sc365529
HSD3B2	Avivasysbio	QC14296
CYP11A1 (D8F4F)	Cell Signaling	sc-393592
CYP17A1	Gift from Prof Alan Conley, UC Davis, USA	
GAPDH	Santa Cruz	G-9
ZO-1	BD	610966
Flag	Sigma	F1804
HA	Sigma	H3663
Myc	Sigma	M4439
V5	Sigma	V8012
SF1 (A-1)	Santa Cruz	sc-393592
GFP	abcam	ab13970
Secondary RDye® 800 Mouse IgG	LICOR	925-32210
Secondary RDye® 800 Rabbit IgG	LICOR	925-32211
Secondary RDye® 800 Chicken IgG	LICOR	925-32218
anti-Rabbit IgG (H+L) AlexaFluor 568	Life technologies	A-11011
anti-Mouse IgG (H+L) AlexaFluor 568	Life technologies	A-11004
anti-Chicken IgY (H+L) AlexaFluor 488	Life technologies	A-11039
Anti-CD29	R&D biosystems	561795
Anti-CD44	R&D biosystems	560977

Anti-CD54	R&D biosystems	560971
Anti-CD73	R&D biosystems	561014
Anti-CD105	R&D biosystems	560839
Anti-CD146	R&D biosystems	561013
Anti-CD166	R&D biosystems	560903
Anti-SSEA-4	R&D biosystems	560126
Anti-CD31	R&D biosystems	560984
Anti-CD34	R&D biosystems	560942
Anti-CD45	R&D biosystems	560976
FITC-Isotype Control	R&D biosystems	555748
PE-Isotype Control	R&D biosystems	551436
**Bacterial and Virus Strains**		
Precision LentiORF SF1 (NR5A1) lentiviral particles	Dharmacon	OHS5900
JM109 Competent Cells	Promega	L2005
**Chemicals, Peptides, and Recombinant Proteins**		
Phosphate Buffered Saline	Sigma Aldrich	79382-50TAB
Penicillin/streptomycin (P/S)	Sigma Aldrich	P4333
Amphotericin B	Sigma Aldrich	A2942
Polyethileneamine reagent (PEI)	Polysciences, Inc	23966
8-bromo-cyclic AMP	Sigma Aldrich	B5386
db-cAMP	Sigma Aldrich	D0260
ACTH	Sigma Aldrich	A0423
Angiotensin II human	Sigma Aldrich	A9525
Retinoic acid (ATRA)	Sigma Aldrich	R2625
Licorice	Sigma Aldrich	G2137
Bombesin	Bachem	H-2155
D-TRP6-LHRH	Gift from Prof. Andrew Schally, University of Miami, USA	
Polybrene	Millipore	TR-1003-G
Hoechst 33259	Invitrogen	H3569
Paraformaldehyde	Acros Organics	416780010
Paraffin	VWR	361077E
Liquid OCT	VWR	361603E
DAB	Vector labs	SK-4105
Avidin-Biotin Complex (ABC)	Vector labs	PK-6100
Vectamount mounting medium	Vector labs	H-5000
Hematoxylin Solution Gill No.3	Sigma Aldrich	GHS332
0.2% ammonia solution	Sigma Aldrich	320145
Eosin	National diagnostics	HS-402
MitoTracker Red CMXRos	Thermo Scientific	M7512
UP-MVG alginate	Novamatrix	4200106
Thrombin	Sigma Aldrich	T4648
Fibrinogen	Sigma Aldrich	F3879
Cumate solution 1000x	Cambridge Bioscience	QM100A-1
Rosiglitazone	Molekula	48650295
Transforming Growth Factor-β1 human	Sigma Aldrich	T7039
Insulin-transferrin-selenium (ITS)	Thermo Fisher	41400045
**Critical Commercial Assays**		
RNeasy Mini Kit	Qiagen	74106
RNase-Free DNase Set	Qiagen	79254
KAPA SYBR fast Universal Kit	KAPA Biosystems	KK4602

Cortisol ELISA Kit	Abcam	ab154996
Telo TAAGG ELISA kit	Roche	11854666910
QIAquick PCR Purification Kit	Qiagen	28104
Cell Counting Kit-8	Sigma Aldrich	96992
**Experimental Models: Cell Lines**		
Human embryonic kidney: HEK293T	ATCC	CRL-3216
**Experimental Models: Organisms/Strains**		
Mouse: C57BL/6NCrl	Charles River Laboratory	027
Mouse: Fox Chase SCID Mouse CB17/lcr-Prkdc^{scid}/lcrlcoCrl	Charles River Laboratory	236
**Oligonucleotides**		
Primers for PCR and qPCR, see Constructs and Primers	This paper	N/A
Primers for molecular cloning, see Constructs and Primers	This paper	N/A
**Recombinant DNA**		
pCDH-CuO-MCS-IRES-GFP-EF1-CymR-T2A-Puro All-in-one inducible IRES lentivector	Cambridge Bioscience	QM812B-1
All constructs newly generated, see Constructs and Primers	This paper	N/A
pHIV-EGFP	Welm et al. 2008	Addgene Plasmid # 21373
HSD11B1-FLAG	Jeanneteau et al. 2008	Addgene Plasmid # 24096
pLV-Azurite	Dull et al., 1998	Addgene plasmid # 36086
pLV-mCherry	Dull et al., 1998	Addgene plasmid # 36084
pT-FLAG	Dimitriev and Vassetzky, 2008	Addgene plasmid # 31385
pAd/CMV/PBX1-IRES-nEBFP2	N/A	Addgene plasmid # 29755
pAd/WT1-IRES-nAmCyan	N/A	Addgene plasmid # 29756
pKMyc	Joberty et al. 2000	Addgene plasmid # 19400
pAd/CMV/DAX1-IRES-nEGFP	N/A	Addgene plasmid # 29752
pENTR4-V5-2 (w234-1)	Campeau et al. 2009	Addgene plasmid # 17426
SB52	Bhattacharya et al 1999	Addgene plasmid # 21487
pCMV flag ERR alpha	Ichida et al. 2002	Addgene plasmid # 10975

Bibliography

Bhattacharya, S., Michels, C.L., Leung, M.-K., Arany, Z.P., Kung, A.L., and Livingston, D.M. (1999). Functional role of p35srj, a novel p300/CBP binding protein, during transactivation by HIF-1. Genes Dev. *13*, 64–75.

Campeau, E., Ruhl, V.E., Rodier, F., Smith, C.L., Rahmberg, B.L., Fuss, J.O., Campisi, J., Yaswen, P., Cooper, P.K., and Kaufman, P.D. (2009). A versatile viral system for expression and depletion of proteins in mammalian cells. PLoS One *4*, e6529.

Dmitriev, P. V, and Vassetzky, Y.S. (2008). A set of vectors for introduction of antibiotic resistance genes by in vitro Cre-mediated recombination. BMC Res. Notes *1*, 135.

Guasti, L., Prasongchean, W., Kleftouris, G., Mukherjee, S., Thrasher, A.J., Bulstrode, N.W., and Ferretti, P. (2012). High plasticity of pediatric adipose tissue-derived stem cells: too much for selective skeletogenic differentiation? Stem Cells Transl. Med. *1*, 384–395.

Ichida, M., Nemoto, S., and Finkel, T. (2002). Identification of a specific molecular repressor of the

peroxisome proliferator-activated receptor gamma Coactivator-1 alpha (PGC-1alpha). J. Biol. Chem. *277*, 50991–50995.

Jeanneteau, F., Garabedian, M.J., and Chao, M. V (2008). Activation of Trk neurotrophin receptors by glucocorticoids provides a neuroprotective effect. Proc. Natl. Acad. Sci. U. S. A. *105*, 4862–4867.

Joberty, G., Petersen, C., Gao, L., and Macara, I.G. (2000). The cell-polarity protein Par6 links Par3 and atypical protein kinase C to Cdc42. Nat. Cell Biol. *2*, 531–539.

Livak, K.J., and Schmittgen, T.D. (2001). Analysis of Relative Gene Expression Data Using Real-Time Quantitative PCR and the 2−ΔΔCT Method. Methods *25*, 402–408.

Martin-Ramirez, J., Hofman, M., van den Biggelaar, M., Hebbel, R.P., and Voorberg, J. (2012). Establishment of outgrowth endothelial cells from peripheral blood. Nat. Protoc. *7*, 1709–1715.

Poliandri, A., Miller, D., Howard, S., Nobles, M., Ruiz-Babot, G., Harmer, S., Tinker, A., McKay, T., Guasti, L., and Dunkel, L. (2017). Generation of kisspeptin-responsive GnRH neurons from human pluripotent stem cells. Mol. Cell. Endocrinol. *447*, 12–22.

Rodríguez-Asiain, A., Ruiz-Babot, G., Romero, W., Cubí, R., Erazo, T., Biondi, R.M., Bayascas, J.R., Aguilera, J., Gómez, N., Gil, C., et al. (2011). Brain Specific Kinase-1 BRSK1/SAD-B associates with lipid rafts: modulation of kinase activity by lipid environment. Biochim. Biophys. Acta - Mol. Cell Biol. Lipids *1811*, 1124–1135.

Schweitzer, K.M., Vicart, P., Delouis, C., Paulin, D., Dräger, A.M., Langenhuijsen, M.M., and Weksler, B.B. (1997). Characterization of a newly established human bone marrow endothelial cell line: distinct adhesive properties for hematopoietic progenitors compared with human umbilical vein endothelial cells. Lab. Invest. *76*, 25–36.

Solito, E., Romero, I.A., Marullo, S., Russo-Marie, F., and Weksler, B.B. (2000). Annexin 1 Binds to U937 Monocytic Cells and Inhibits Their Adhesion to Microvascular Endothelium: Involvement of the α4β1 Integrin. J. Immunol. *165*.

Welm, B.E., Dijkgraaf, G.J.P., Bledau, A.S., Welm, A.L., and Werb, Z. (2008). Lentiviral Transduction of Mammary Stem Cells for Analysis of Gene Function during Development and Cancer. Cell Stem Cell *2*, 90–102.
